# Adaptive Evolution and the Birth of CTCF Binding Sites in the *Drosophila* Genome

**DOI:** 10.1371/journal.pbio.1001420

**Published:** 2012-11-06

**Authors:** Xiaochun Ni, Yong E. Zhang, Nicolas Nègre, Sidi Chen, Manyuan Long, Kevin P. White

**Affiliations:** 1 Institute for Genomics and Systems Biology, University of Chicago, Chicago, Illinois, United States of America; 2 Department of Ecology and Evolution, University of Chicago, Chicago, Illinois, United States of America; 3 Department of Human Genetics, University of Chicago, Chicago, Illinois, United States of America; Fred Hutchinson Cancer Research Center, United States of America

## Abstract

Changes in the physical interaction between *cis*-regulatory DNA sequences and proteins drive the evolution of gene expression. However, it has proven difficult to accurately quantify evolutionary rates of such binding change or to estimate the relative effects of selection and drift in shaping the binding evolution. Here we examine the genome-wide binding of CTCF in four species of *Drosophila* separated by between ∼2.5 and 25 million years. CTCF is a highly conserved protein known to be associated with insulator sequences in the genomes of human and *Drosophila*. Although the binding preference for CTCF is highly conserved, we find that CTCF binding itself is highly evolutionarily dynamic and has adaptively evolved. Between species, binding divergence increased linearly with evolutionary distance, and CTCF binding profiles are diverging rapidly at the rate of 2.22% per million years (Myr). At least 89 new CTCF binding sites have originated in the *Drosophila melanogaster* genome since the most recent common ancestor with *Drosophila simulans*. Comparing these data to genome sequence data from 37 different strains of *Drosophila melanogaster*, we detected signatures of selection in both newly gained and evolutionarily conserved binding sites. Newly evolved CTCF binding sites show a significantly stronger signature for positive selection than older sites. Comparative gene expression profiling revealed that expression divergence of genes adjacent to CTCF binding site is significantly associated with the gain and loss of CTCF binding. Further, the birth of new genes is associated with the birth of new CTCF binding sites. Our data indicate that binding of *Drosophila* CTCF protein has evolved under natural selection, and CTCF binding evolution has shaped both the evolution of gene expression and genome evolution during the birth of new genes.

## Abstract 2

Comparative ChIP-seq data reveal adaptive evolution of insulator protein CTCF binding in multiple *Drosophila* species.

## Author Summary

A large proportion of the diversity of living organisms results from differential regulation of gene transcription. Transcriptional regulation is thought to differ between species because of evolutionary changes in the physical interactions between regulatory DNA elements and DNA-binding proteins; these can generate variation in the spatial and temporal patterns of gene expression. The mechanisms by which these protein–DNA interactions evolve is therefore an important question in evolutionary biology. Does adaptive evolution play a role, or is the process dominated by neutral genetic drift? Insulator proteins are a special group of DNA-binding proteins—instead of directly serving to activate or repress genes, they can function to coordinate the interactions between other regulatory elements (such as enhancers and promoters). Additionally, insulator proteins can limit the spreading of chromatin condensation and help to demarcate the boundaries of regulatory domains in the genome. In spite of their critical role in genome regulation, little is known about the evolution of interactions between insulator proteins and DNA. Here, we use ChIP-seq to examine the distribution of binding sites for CTCF, a highly conserved insulator protein, in four closely related *Drosophila* species. We find that genome-wide binding profiles of CTCF are highly dynamic across evolutionary time, with frequent births of new CTCF-DNA interactions, and we demonstrate that this evolutionary process is driven by natural selection. By comparing these with RNA-seq data, we find that gain or loss of CTCF binding impacts the expression levels of nearby genes and correlates with structural evolution of the genome. Together these results suggest a potential mechanism of regulatory re-wiring through adaptive evolution of CTCF binding.

## Introduction

Gene regulation is a major driver in the generation of morphological diversity [1],[2]. Transcriptional regulators determine spatial and temporal patterns of mRNA level by binding to *cis-*regulatory DNA elements. Many previous studies have demonstrated that changes at the level of protein-DNA interactions can account for specific phenotypic differences observed in nature [1],[3]. Genome-wide studies have shown that binding of transcriptional regulators evolves substantially between different species [4]–[9]. Although in *Drosophila*, the binding profiles of some regulatory factors involved in embryonic development, such as the Twist protein, are relatively more conserved [5],[8]. Yet it remains an open question to what extent such protein–DNA binding evolution is adaptively and functionally significant or whether it reflects drift. In order to address this question, regulatory factors must be mapped in multiple related species and the results interpreted in the light of intraspecific and interspecific *cis*-regulatory DNA variation.

Insulator proteins participate in the marking of boundaries for genomic regulatory units by binding to DNA insulator elements [10]–[13]. These protein–DNA complexes are thought to function as barriers against the spread of heterochromatin or to regulate enhancer–promoter communications by preventing inappropriate interactions, although the precise molecular mechanism by which they act is not known [12],[14],[15]. Recent studies have suggested that insulator complexes may also participate in the global nuclear organization of active and inactive chromatin domains via mediating intra-/interchromosomal interactions [16]–[19]. The broad functions possessed by insulator proteins make them a key player in transcriptional regulation, and significant efforts have been made to elucidate where they interact with DNA in multiple species [13],[18],[20]–[24].

CTCF (CCCTC binding factor) is the only known DNA binding insulator protein conserved between human and fly [25]. In vertebrates, this 11 zinc-finger protein is shown to be crucial in processes of epigenetic imprinting [26],[27], X chromosome inactivation [28], and associated with various complex human diseases including cancer and diabetes [29]–[31]. Genome-wide studies revealed that CTCF widely associates with human chromosomes [20], and its binding profile is reported as individual and allele specific [32] with considerable variation between different cell lines [18]. In *Drosophila melanogaster*, as one of the five known insulator proteins, CTCF binds to the well-characterized insulator elements in the *Bithorax complex* region, which demarcate different *cis*-regulatory units corresponding to different parasegmental expression patterns of three important developmental genes: *Ubx*, *Abd-A*, and *Abd-B* [33]–[36]. Genome-wide chromatin immunoprecipitation (ChIP) studies performed in *Drosophila melanogaster* revealed a consensus motif similar to the human and vertebrate ones [13],[22],[23]. Limited cell-type-specific binding of CTCF was also observed in these studies [13],[22]. The fact that CTCF is a conserved protein with a major role in gene regulation and genome organization makes it an appealing candidate to evaluate how changes in the DNA sequence drive conservation, birth, and death of functional CTCF binding sites and the subsequent impact of these changes on gene regulation. Further, a very recent comparative study in multiple mammalian lineages shows that CTCF binding evolution in mammals is likely to be driven by retrotransposon expansions and that newly gained CTCF binding events are functional [9]. However, in *Drosophila*, it is not known whether CTCF binding evolution follows a similar pattern or if it is independent of transposable element (TE) activity.

To study the evolution of genome-wide CTCF protein binding in *Drosophila*, we carried out comparative ChIP-seq experiments in four closely related species: *D. melanogaster*, *D. simulans*, *D. yakuba*, and *D. pseudoobscura*. The three species *D. simulans*, *D. yakuba*, and *D. pseudoobscura* diverged from *D. melanogaster* about 2.5, 6, and 25 million years ago [37], respectively, providing the opportunity to observe binding dynamics in a context of increasing evolutionary distances.

## Results

### CTCF Binding Profiles in Different *Drosophila* Species

To map CTCF binding in the genomes of *D. melanogaster*, *D. simulans*, *D. yakuba*, and *D. pseudoobscura*, we used chromatin collected from white pre-pupae (WPP) at puparium formation, a developmental stage induced by rising titres of the metamorphosis hormone 20-hydroxyecdysone [38]. WPP has easy-to-distinguish morphology, and this stage lasts only about 20 minutes, thus allowing the collection of developmentally synchronized animals within and between species. For each species, we performed ChIP in triplicate with previously characterized CTCF antibodies (Figure S1, [23]) and obtained between 3 and 9 million uniquely mapped 36 bp sequence reads for each ChIP (ChIP-seq) and corresponding input samples (Table S1A).

**Figure 1:**
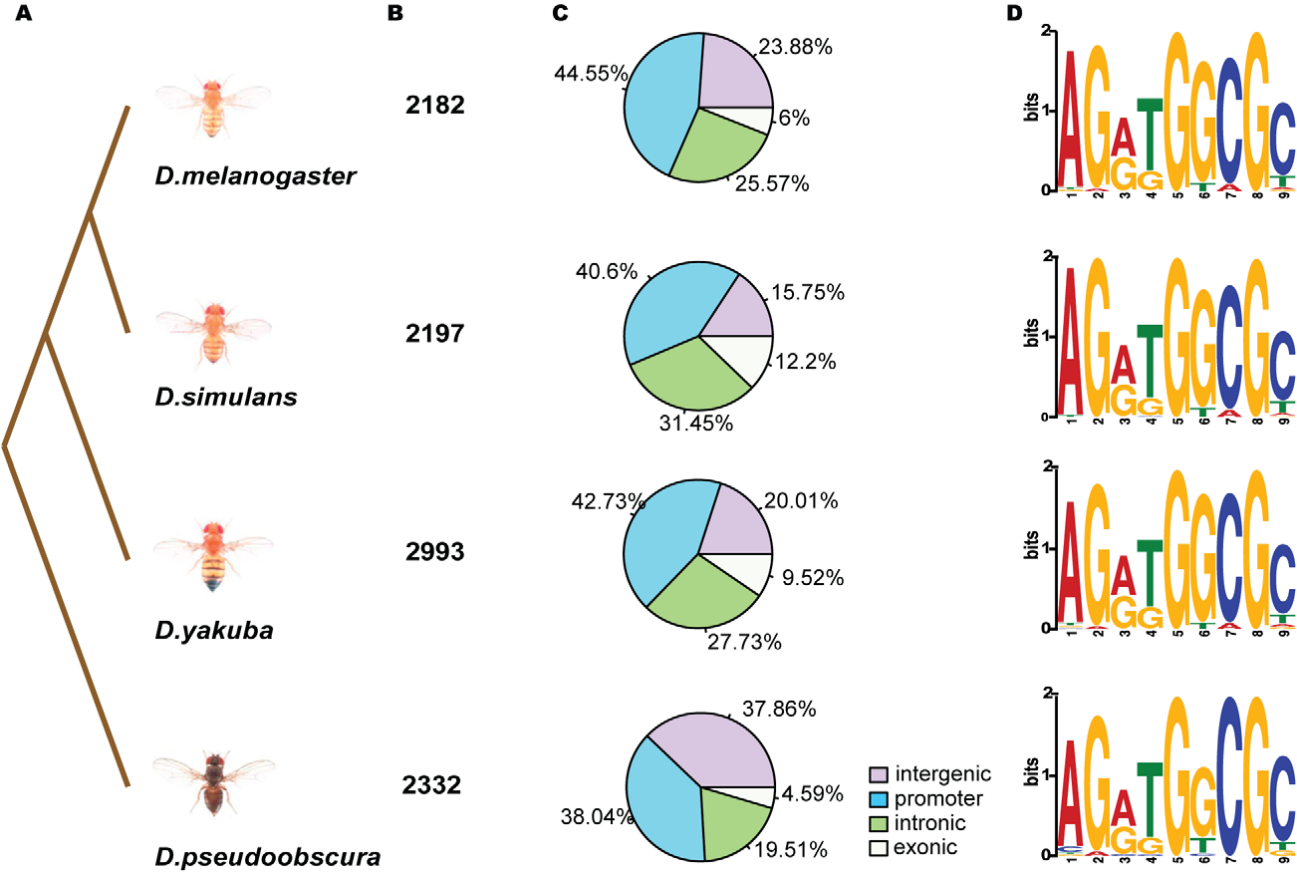
Conserved binding preference of CTCF. (A) Topological illustration of the phylogenetic relationships between the four *Drosophila* species in our study. (B) The number of CTCF binding peaks identified in ChIP-seq experiments in the four *Drosophila* species. (C) Genomic distribution of CTCF binding sites in the four *Drosophila* species. The percentages of CTCF binding sites distributed in different genomic locations are shown in the four pie charts: intergenic (>1 kb to nearest TSS, purple), promoter (<1 kb to nearest TSS, light blue), intronic (light green), and exonic (white). In all four species, >90% of the binding sites reside in the noncoding regions with highest percentages in promoter regions. (D) Species-specific binding motifs. The 9 bp core motif for each species is de novo generated by MEME using the top 2000 ChIP-seq-enriched CTCF binding site DNA sequences.

CTCF binding profile replicates within a species for the same strain were highly reproducible (median Spearman's rank correlation coefficients for peak regions between replicates within *D. melanogaster*, *D. simulans*, *D. yakuba*, and *D. pseudoobscura* are 0.89, 0.87, 0.84, and 0.71, respectively; average genome-wide Pearson's correlation coefficients are 0.91, 0.88, 0.91, and 0.81; Figure S4, Table S4) and recapitulated the well-characterized binding peaks previously identified within the *Bithorax complex* genomic region in *D. melanogaster* (Figure S2) [13],[23]. We modified the ChIP-seq analysis program QuEST [39] to apply to the triplicate data (Figure S3, also see Materials and Methods), and at a False Discovery Rate (FDR) <1%, our analyses yielded between 2,000 to 3,000 peaks in each of the four species (Figure 1B). With these sets of CTCF binding sites, we compared their genomic distributions as well as the enriched DNA sequence motifs for each species.

CTCF shows similar binding distributions in intergenic, promoter, intronic, and exonic sequences among the four species (Figure 1C) and in a pattern consistent with previous genomic mapping studies of CTCF in both fly and human [13],[20]. Importantly, the position weight matrixes of the consensus motifs for CTCF-bound sequences are virtually identical among the four species (Figure 1D). These motifs are also similar to the in vitro identified CTCF consensus motif [27] and to previously identified motifs from *Drosophila*, human, and other vertebrates [13],[20],[23]. CTCF protein evolution is highly constrained (Table S2, Figure S5), especially the 253 amino acid DNA binding domain (Figure S5), for which there are only 1, 4, and 38 amino acids diverged between *D. simulans*, *D. yakuba*, *D. pseudoobscura*, and *D. melanogaster*, respectively. We calculated the occurrences of each species-specific motif in the CTCF binding sites and obtained similar percentages among the four species at various thresholds (Table S3). These results confirm the conservation of CTCF binding motifs among *Drosophila* species and indicate that any evolutionary patterns we observe are most likely due to changes in the *cis*-regulatory target DNA sequences of CTCF.

### CTCF Binding Evolves Rapidly

**Figure 2:**
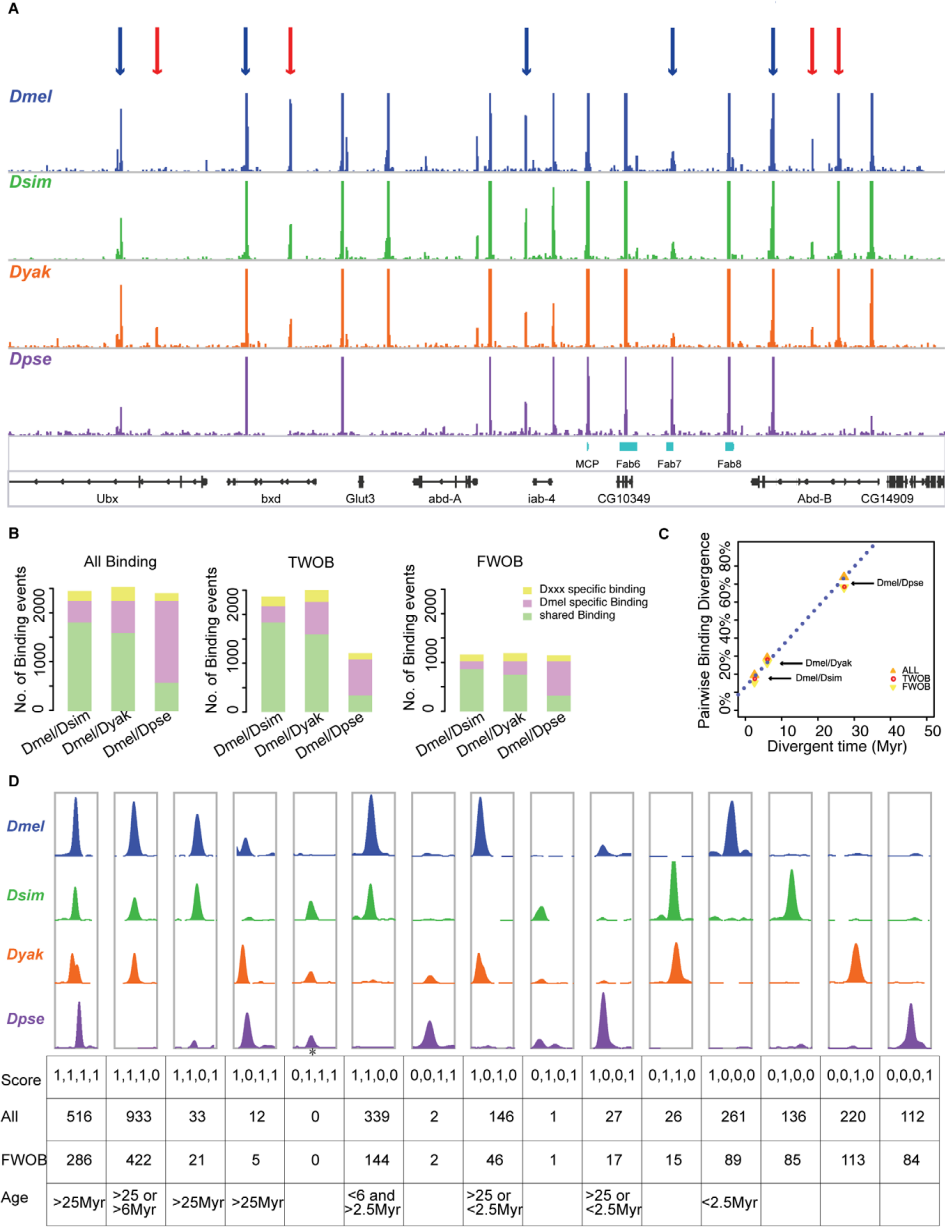
Diverged CTCF binding between *Drosophila* species. (A) Evolutionary dynamics of CTCF binding profiles at the *Bithorax complex* region. The four colored wiggle file tracks show the ChIP CDP enrichment scores estimated from our quantitative analysis pipeline for the four species: *D. melanogaster* (blue), *D. simulans* (green), *D. yakuba* (orange), and *D. pseudoobscura* (purple). The four tracks are at the same scale, with the height of each curve at each coordinate denoting the enrichment score values. In the top panel, the blue arrows point to examples of conserved binding events across the four species, and the red arrows point to examples of diverged binding events between species. The fifth track shows the boundaries of previously identified insulator elements (in sky blue). The last track shows the genes in the genomic region. (B) Number of conserved and diverged binding events. From left to right, the three bar plots show the number of *D. melanogaster*–specific (pink), shared (blue), and non–*D. melanogaster* (D.xxx, yellow) specific binding events between each of the species pairs (*D. melanogaster/D. simulans*, *D. melanogaster/D. yakuba*, and *D. melanogaster/D. pseudoobscura*) for all binding events possibly identified (All, left), Two-Way Orthologous Binding events (TWOB, middle), and Four-Way Orthologous Binding events (FWOB, right). TWOB is defined as a binding event identified in regions where the sequence identity between the two compared species is >50%. FWOB is defined as a binding event identified in regions where the sequence identity across all four species is >50%. (C) Linear increase of pair-wise binding divergence with species divergent time. The binding divergence is calculated as the percent of *D. melanogaster* binding events not shared with the non–*D. melanogaster* species in each pair-wise comparison. Different shaped and colored points represent different groups of binding events as indicated by the legend. The red dashed line depicts the fitted linear regression line of TWOB binding divergence with divergent time. (D) Evolutionary groups of CTCF binding events. Top panel, representative dynamic binding profiles in the four *Drosophila* species (*D. melanogaster*, blue; *D. simulans*, green; *D. yakuba*, orange; *D. pseudoobscura*, purple) illustrating examples of 15 mutually exclusive evolutionary groups of binding status. The height at each binding curve denotes the ChIP CDP enrichment score estimated from our analyses pipeline. For each evolutionary group, the *y*-axes of the four binding curves are at the same scale. The first row of the lower table shows the Boolean conservation score corresponding to the binding profiles, where 0 indicates absence of binding event and 1 indicates the presence of binding events. The second and third rows of the lower table summarize the number of all binding events (second row) and FWOB events (third row) falling into each evolutionary group. The last row of the lower table shows the inferred evolutionary age for different groups of *D. melanogaster* binding events using Parsimony methods. * As for the evolutionary group with boolean conservation score 0,1,1,1, there is no instance identified in our analyses, so the representative binding profile in the figure is generated by artificially modifying another binding profile to represent the specific category.

A straightforward way to assess binding conservation or divergence is to directly compare the boundaries of identified peak regions between each species (Table S7, also see Materials and Methods) with genome-wide alignment. However, by using independent analyses in each species, actual conserved binding sites are likely to be identified as diverged between species due to false negatives being scored as “diverged.” To avoid this problem and to quantitatively explore the evolutionary dynamics of CTCF binding profiles across species (Figure 2A), we developed a *D. melanogaster*–centric analysis approach to examine the between-species CTCF occupancy on orthologous DNA sequences in light of within-species binding variation (Figure S6, also see Materials and Methods). In brief, instead of directly comparing the binding region boundaries between each species, the approach we took translated uniquely mapped sequence reads in the non–*D. melanogaster* species into *D. melanogaster* genome (Table S1B), thus allowing quantitative modeling of within- and between-species read number data using an ANOVA-like linear categorical model to partition variances of local read number data in each binding region. We thus identified *D. melanogaster*–specific, non–*D. melanogaste*r–specific, and shared binding events for each paired species accordingly (Figure 2B, Table S5). Our method yields highly reliable conservation and divergence information of the *D. melanogaster* binding sites between each species since the False Positive Rate (identifying shared binding sites as *D. melanogaster* specific) of the linear categorical model is estimated to be 0.35% using simulated data (Materials and Methods) and the overall analysis pipeline error rate (False Positive Rate plus False Negative Rate) is estimated to be less than 2% using a “gold standard” data set (Materials and Methods). However, since different *Drosophila* genomes have different assembly and annotation quality, which are all based on and are poorer than the *D. melanogaster* genome, inevitably the translated read number data from some regions of the non–*D. melanogaster* species will be smaller than they would be in an ideal situation. As a result, there is generally reduced power in detecting non–*D. melanogaster*–specific binding events compared to *D. melanogaster*–specific and shared binding events (Figure 2B, Table S4).

Because loss of orthologous sequences among species is often driven by large-scale genome evolution instead of local nucleotide substitutions or small insertion-deletions (indels) [40], diverged CTCF binding events in regions with or without orthologous sequences have different biological interpretations. With a criterion of at least 50% sequence identity for orthology assignment, we identified binding events in orthologous regions between each species pair, which we refer to as two-way orthologous binding (TWOB). Similarly, we identified binding events in regions with orthologous sequence counterparts in all four species and refer to those as four-way orthologous binding (FWOB).

With the analysis pipeline described above, we identified 2,267 binding events for *D. melanogaster* (Figure 2B, Table S5). Since genome assembly imperfections and gaps among the non–*D. melanogaster* species lead to an underestimate of binding events in these genomes, we used the percentage of diverged binding events with respect to *D. melanogaster*, which has the best refined genome assembly map, to measure pair-wise binding divergence. Naturally, the rate of binding site evolution must be greater than the error rate (<2%) in order to be detected. For the different species pair-wise comparisons with *D. melanogaster*, approximately 20%, 30%, and 70% (19.67%, 29.11%, and 74.06% of all binding sites; 17.34%, 28.05%, and 68.37% of TWOBs; and 15.24%, 26.31%, and 68.06% of FWOBs) were identified as diverged from *D. simulans*, *D. yakuba*, and *D. pseudoobscura*, respectively (Figure 2B and 2C, Table S5). These values are not only consistent with each other but also highly comparable to divergence rates estimated with different parameters (Table S5), or with a subset of sites filtered for high input sequence coverage (“high sequence coverage sites”) to ensure that the binding evolution was not an artifact of low sequence coverage in one or more of the species (Table S6) or using alternative methods (Tables S7 and S8). When plotted against species divergence time, these values show a clear linear trend (Figure 2C). We fit a simple linear regression for the TWOBs, and we estimated the divergence rate of CTCF binding as 2.22% per Myr (Student's test, *p*<0.05, *R*-squared>0.99). This divergence rate is lower than synonymous substitution rates (6.34% per Myr, [40]) but substantially higher than the protein sequence divergence rate (1.19% per Myr, [40]) and non-synonymous nucleotide substitution rate (0.4% per Myr, [40]) in *Drosophila*, indicating that, although constrained, CTCF binding evolves relatively rapidly. This linear pattern of binding divergence remains stable when different peak calling stringencies were applied (Tables S5 and S6).

Estimates of binding divergence/conservation rates can depend on the choice of analysis methods, which have different associated false positive and false negative rates. In order to gain an unambiguous comparison of binding divergence between CTCF and other transcription factors in *Drosophila*, we also applied our analysis pipeline to previously published Twist comparative data [8] and reciprocally applied the He et al. method [8] to our data (Tables S8, S9, and S10). In both comparisons, we obtained a larger estimate of binding divergence in the CTCF data (Tables S8 and S10), indicating that CTCF binding is evolving faster than binding of the developmental regulatory transcription factor Twist.

### New CTCF Binding Sites Originate Frequently

By combining between-species binding appearance and absence results, we grouped CTCF binding events into 15 different evolutionary categories (Figure 2D). Regardless of evolutionary category, we found that CTCF binding events are distributed similarly between various genomic locations (Table S11), showing no biases in binding evolution according to genomic position. We next inferred the evolutionary age of each *D. melanogaster* binding site by assigning its origination on the *Drosophila* phylogeny with parsimony (Figure 2D, Table S12). Whether we examined all binding sites or considered only FWOBs, more than 60% (1,533/2,267 for all binding and 655/1,030 for FWOBs) of *D. melanogaster* binding sites originated after the split of the *melanogaster* group from *pseudoobscura* group, and thus less than 40% were inherited from the common ancestor of these two major clades (Figure 2D). Notably 89 FWOBs were newly gained specifically on the *D. melanogaster* branch (Figure 2D), leading to a conservative estimate of ∼36 binding gains per million years (89 binding events/2.5 Myr since last common ancestor of *D. melanogaster* and *D. simulans*). Interestingly, there are only 39 *D. melanogaster*–specific new genes identified [41],[42], resulting in an average of ∼16 gene gains per Myr. This result indicates that in *Drosophila* new regulatory elements bound by CTCF are evolving at a higher rate than new genes. In contrast to the large number of newly gained binding sites, there are no FWOBs identified as lost in *D. melanogaster* (Figure 2D). Although underdetection of non–*D. melanogaster* binding sites in our analysis could lead to failure in observing *D. melanogaster* lineage-specific loss, the asymmetric patterns of CTCF binding site gains are also observed along the *D. simulans* branch (85 gains and five losses, Figure 2D), the *D. yakuba* branch (113 gains and 21 losses, Figure 2D), or when taking only the three species in the *melanogaster* group into consideration (Figure S7) or when using only high-sequence coverage sites (Table S13), indicating that gain of binding is evolutionarily favored. This pattern is not likely due to ascertainment biases of highly conserved genomic regions associated with FWOBs, because we also observed large numbers of binding gains and small numbers of binding losses for binding events genome-wide (i.e., the pattern holds for sites that have not been filtered for sequence conservation) (Figure 2D, Figure S7, Table S13). Such a robust pattern suggests that positive selection may be driving the creation of new sites, which we sought to explore further.

### CTCF Binding Evolution Is Correlated with Sequence Evolution

**Figure 3:**
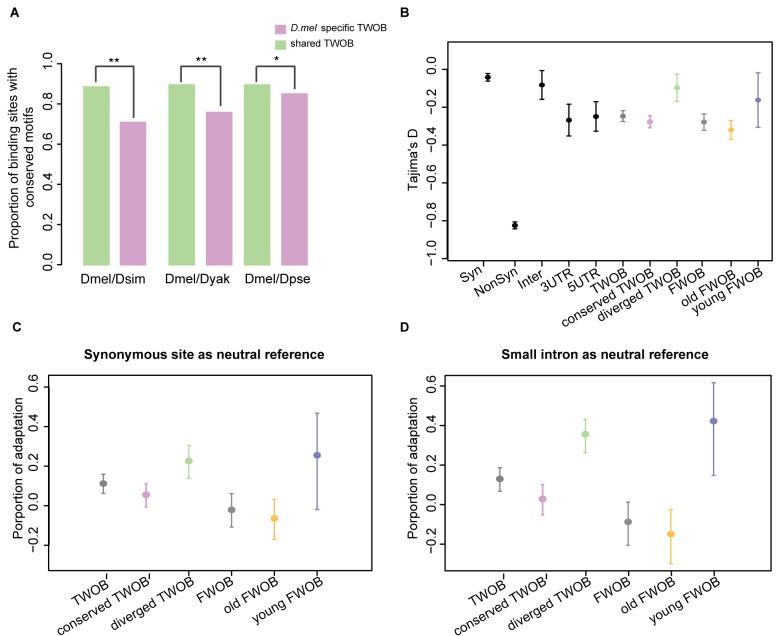
Selection on CTCF motif sites. (A) Proportion of binding sites with conserved motifs. The bar plots show proportions of *D. melanogaster*–specific (pink) and shared (green) binding sites that have conserved motifs between each species pair. A binding site is defined as having conserved motifs if there is at least one species-specific motif identified in the corresponding orthologous sequences. The *p* value cutoff for FIMO motif searching here is 0.005. For any species pair, the proportion of conserved (here shared) binding sites having conserved motifs is significantly higher than the diverged (here *D. melanogaster*–specific) binding sites. Significance levels: * *p*<0.05; ** *p*<0.01, two-sided Fisher's exact test. (B) Mean Tajima's D values for CTCF-motif sites. Tajima's D values were calculated using 37 *D. melanogaster* North American strains' polymorphism data for various groups of CTCF-motif sites, the synonymous and nonsynonymous sites of nearest genes, and randomly sampled 3′UTR, 5′UTR, and intergenic 9 bp sites. The center of each filled circle depicts the mean Tajima's D value for each group, with the error bar indicating 2 standard deviations. (C and D) Estimated shared proportion of adaptation with neutral reference to nearest gene synonymous sites (C) and a set of small introns (D). *D. yakuba* sequences were used as an out-group for estimating alpha values for different groups of CTCF-motif sites using an extension of the MK test framework. The filled colored circles depict the shared alpha value estimated within each group, with the error bar indicating the 95% confidence interval. Label abbreviations: Syn, synonymous sites of nearest genes of CTCF binding sites; Nonsyn, non-synonymous sites of nearest genes of CTCF binding sites; TWOB, CTCF-motif sites associated with two-way orthologous binding events between *D. melanogaster* and the out-group; conserved TWOB, CTCF-motif sites associated with conserved two-way orthologous binding events; diverged TWOB, CTCF-motif sites associated with *D. melanogaster*–specific two-way othologous binding events; FWOB binding, sites associated with four-way orthologous binding events; Young FWOB, sites associated with FWOBs, for which the age is estimated to be <2.5 Myr; old FWOB, sites associated with FWOBs, for which the age is estimated to be >6 Myr.

We investigated sequence divergence of *cis*-DNA elements associated with CTCF binding evolution, since CTCF protein and its binding preference are highly conserved (Figure 1C, Figure S6, Table S2). We examined the 201 bp elements comprised of the summit coordinate of each binding peak plus the two 100 bp flanking regions (i.e., CTCF-201 sites). We found that the median PhastCon scores [43] of the conserved *D. melanogaster* binding sites are significantly higher than those of the diverged sites (Figure S8A). A similar pattern was observed when we calculated the percentage of between-species sequence identity (Figure S8B), indicating that CTCF binding evolution is correlated with levels of sequence conservation. Because motifs are special sequence features associated with protein–DNA interactions, we next examined the relationships between motif evolution and CTCF binding evolution. For each species pair, we counted the number of species-specific motif occurrences in the corresponding orthologous sequences of each binding site. Binding sites that contained at least one motif in both sequences were defined to have conserved motifs. This is a crude way of defining motif conservation in binding sites, but nonetheless we still observed a significantly higher proportion of conserved binding sites that contain conserved motifs than diverged binding sites (two-sided Fisher's exact test, *p*<0.05, Figure 3A), confirming that *cis-*regulatory target sequences are correlated with CTCF binding evolution.

### CTCF Binding Evolution Is Shaped by Natural Selection

We next examined genomic DNA variation associated with CTCF binding events for signatures of selection. We first assessed whether purifying selection may play a role in shaping CTCF binding evolution. Purifying selection acting on polymorphic variants is expected to keep them at lower frequencies in a population, leading to a relatively higher number of segregating sites and therefore a more negatively skewed Tajima's D value than expected under neutrality [44]. We calculated Tajima's D using DNA polymorphism data from 37 *D. melanogaster* inbred lines (www.dpgp.org) for the core consensus binding motifs (CTCF-motif sites) identified within the *D. melanogaster* TWOBs and FWOBs. As expected, the distribution of Tajima's D values for nonsynonymous sites is negatively skewed compared to the synonymous sites (Wilcox rank sum test, *p*<2.2e-16; Figure 3B). This pattern also extends to the CTCF-motif sites when they are compared to synonymous sites in neighboring protein-coding genes (Wilcox rank sum test, *p*<0.0001; Figure 3B). Interestingly, the distribution of Tajima's D values for CTCF motifs within CTCF binding sites is comparable to 3′UTR and 5′UTR sequences, while it is significantly more negatively skewed than intergenic sequence (Wilcox rank sum test, *p*<0.02). Thus, CTCF binding appears to be subject to stronger purifying selection than synonymous and intergenic genomic sequences. To explore whether these trends varied depending on evolutionary conservation of binding, we separated the TWOB CTCF-motif sites into subgroups associated with conserved binding (conserved TWOB) and diverged binding (diverged TWOB). We observed a more negatively skewed Tajima's D distribution in the former group (Wilcox rank sum test, *p*<0.01; Figure 3B). Similar analyses of CTCF motifs within FWOB binding sites were carried out by designating binding events with evolutionary age <2.5 Myr as young FWOB and >6 Myr as old FWOB. Again a more negatively skewed Tajima's D distribution was observed in the old FWOB group (Wilcox rank sum test, *p* = 0.11; Figure 3B). We observed similar patterns of Tajima's D in the CTCF-201 sites (Figure S9) as well as in high-sequence coverage sites (Figure S10). These results indicate that the more conserved CTCF binding sites are subject to stronger purifying selection and therefore are more constrained than the less conserved sites, as one might expect.

Using the same polymorphism data and employing *D. yakuba* as an outgroup, we counted the number of fixed and polymorphic nucleotides within CTCF motifs present within different classes of binding sites. Overall, significant excesses of fixed nucleotide changes are observed in groups of CTCF binding sites (except FWOB and Old_FWOB groups) when compared to synonymous nucleotide changes at nearby genes (Chi-square test, *p*<0.001; Tables S14 and S15), indicating that positive selection has shaped CTCF binding evolution. By extending the McDonald-Kreitman test framework [45]–[47], we estimated α, the proportion of between-species divergence fixed by positive selection for each subgroup of sites (as described earlier for Tajima's D; Figure 3C). We found that the young FWOB sites show a significantly higher shared α value (0.25, *p*<0.0001) than the old FWOB sites (0.25 versus −0.0673; log likelihood ratio test for group comparison, *p*<0.05; Figure 3C), and a similar trend was observed between diverged TWOB (0.2237, *p*<0.0001) and conserved TWOB sites (0.2237 versus 0.0526; log likelihood ratio test for group comparison, *p*<0.05; Figure 3C). Since synonymous sites are usually constrained by codon usage [48], we also used a set of pre-characterized small introns that are believed to have evolved neutrally [49] as a neutral reference. Again we observed that the diverged TWOB (0.3598, *p*<0.0001) and young FWOB sites (0.4265, *p*<0.0001) shared significantly higher α values than their counterparts (log likelihood ratio test for group comparison, all *p*<0.001; Figure 3D). The same pattern remains when using *D. simulans* as an outgroup (Figure S12) or using CTCF-201 sites for the calculation (Figure S11). This trend of higher shared α from more diverged sites is also observed in the high-sequence coverage sites (Figure S13). These observations indicate that gain of new CTCF binding events are likely driven by positive selection. To further confirm the role of positive selection in the birth of new CTCF binding events, we carried out a multilocus HKA test ([50]; Materials and Methods). By comparing the young sites to the old sites as well as the neutral small introns, we observed significantly reduced polymorphism in the young sites, suggesting strong directional positive selection (Tables S16 and S17).

In order to test whether this phenomenon of selection-driven binding evolution is CTCF specific or more general, we applied the same population genetic analysis to the available comparative data for Twist [8]. Consistent with the higher binding conservation level, we observed a stronger purifying selection signal and weaker positive selection signal in Twist binding sites than in CTCF binding sites (Figure S20). Interestingly, we found a similar pattern of a higher positive selection signature in the diverged binding sites than the conserved sites for Twist (Figure S20).

### CTCF Binding Evolution Is Associated with Expression Divergence

Since CTCF participates in transcriptional control through organizing and delineating regulatory domains [10]–[13],[19] and gain of CTCF binding appears to be driven by positive selection, naturally we wondered if there were any detectable effects on gene expression. We measured mRNA transcript abundances for WPP samples in *D. melanogaster*, *D. simulans*, and *D. yakuba* using RNA-seq (Table S1C, Figure S18) and estimated the interspecies expression change for every orthologous gene pair between *D. melanogaster/D. simulans* and *D. melanogaster/D. yakuba* through a generalized linear model framework, cataloging the evolutionary status of each gene as either “stable” or “diverged” (Materials and Methods) thereafter.

**Figure 4:**
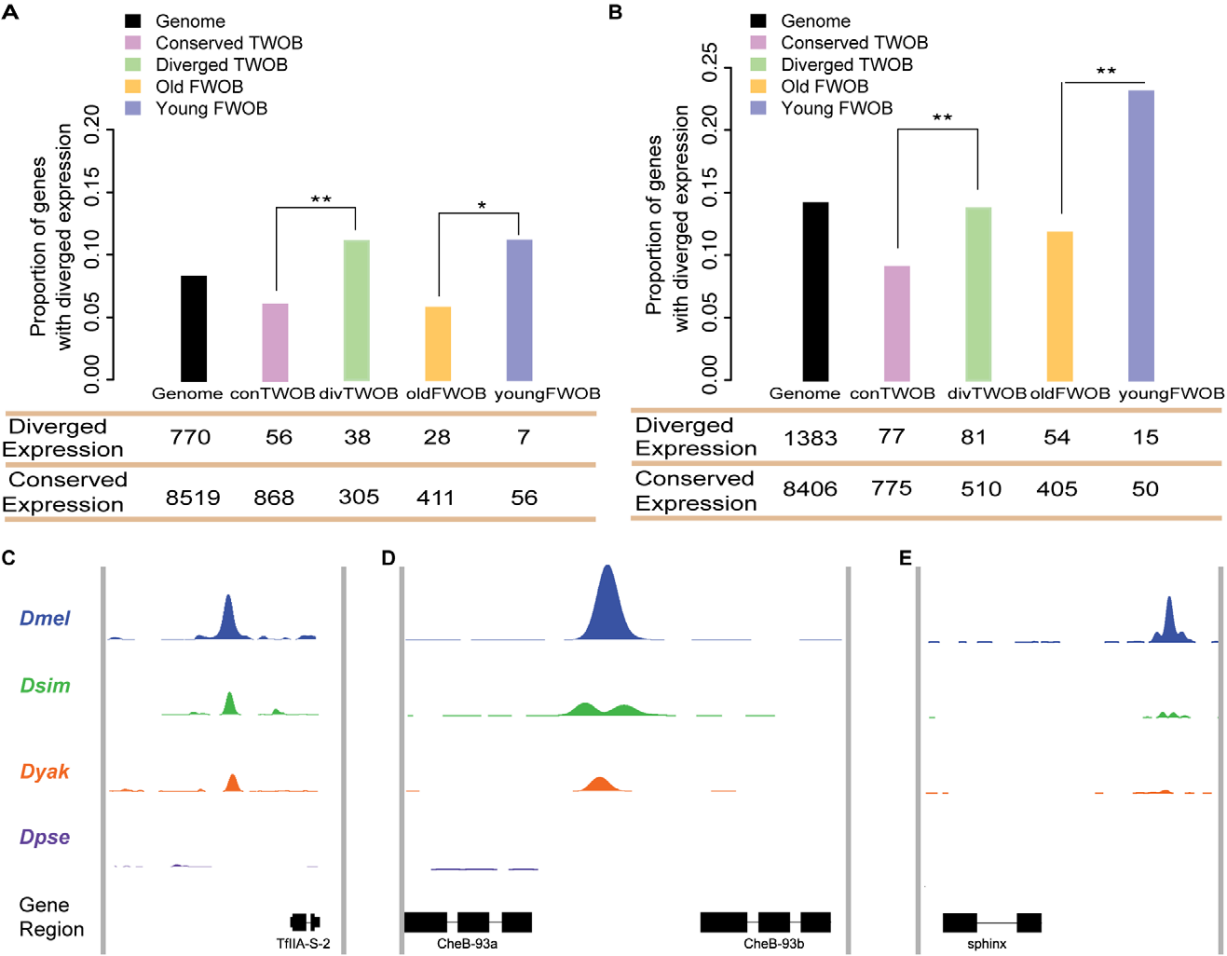
Functional consequences of CTCF binding evolution. (A–B) CTCF binding evolution is associated with gene expression evolution. The bar plots show the proportion of genes with diverged expression between (A) *D. melanogaster/D. simulans* and (B) *D. melanogaster/D. yakuba* comparisons associated with different groups of CTCF binding sites: Genome-wide (black), Conserved TWOB (pink), Diverged TWOB (green), Old FWOB (orange), and Young FWOB (light purple). The table below each bar plot shows the number of genes with diverged and conserved gene expression in the corresponding comparisons and associated with the corresponding CTCF binding sites. For each groups of CTCF binding sites, the associated genes are the union of the nearest gene to each binding site. The evolutionary status of gene expression (conserved or diverged) is determined using triplicate WPP mRNA-seq data through a generalized linear regression framework. Label abbreviations are the same as described in Figure 3. Significance levels: * *p*<0.05; **p<0.01; one-sided Fisher's exact test. (C–E) CTCF binding evolution is correlated with new gene origination. The four colored wiggle tracks in each of the plots show the ChIP CDP enrichment scores of the four species (*D. melanogaster*, blue; *D. simulans*, green; *D. yakuba*, orange; *D. pseudoobscura*, purple) across different genomic regions. CTCF binding peaks are observed in *D. melanogaster*, *D. simulans*, and *D. yakuba* at flanking genomic regions of newly evolved genes *TFII-A-S2* (C) and *CheB93a* (D). The two genes both originated after the split of the *melanogaster* group with the *pseudoobscura* group. CTCF binding peak is only observed in the *D. melanogaster* genome in the flanking genomic regions of *D. melanogaster* lineage-specific gene *sphinx* (E).

We focused on the nearest genes to the *D. melanogaster* TWOB sites and grouped them into genes near conserved TWOB sites and genes near diverged TWOB sites. Since diverged TWOBs resulted from either binding gain in *D. melanogaster* or binding loss in *D. simulans* or *D. yakuba*, regulation of these genes by CTCF might have been altered. Consistent with this hypothesis, the proportion of genes with diverged expression near diverged TWOB sites is significantly greater than near conserved TWOB sites (Fisher's exact test, *p*<0.01; Figure 4A,B). We obtained a similar result when comparing between genes near young FWOB sites and genes near old FWOB sites (Fisher's exact test, *p*<0.05; Figure 4A,B). Moreover, the proportions of genes with diverged expression near conserved TWOB and near old FWOB sites are smaller than the genome-wide average (Fisher's exact test, *p*<0.05; Figure 4A,B). Such correlation is also observed when using microarray data for inferring gene expression divergence (Figure S14) as well as when using high-sequence coverage sites (Figure S15). These observations indicate that CTCF binding evolution impacts gene expression evolution, which previously has been shown to evolve rapidly and to be shaped by selection in these species at the WPP stage [51],[52].

Selection on gene expression can lead to adaptive evolutionary signatures in *cis-*regulatory elements. Indeed, in *Drosophila*, adaptive gene expression has been linked to adaptive *cis-*DNA evolution [53]. We thus hypothesized that the stronger positive selection signature observed in the diverged TWOBs might stem from the sites being associated with diverged expression that has more directly been subject to natural selection. We calculated and compared α values for two additional subgroups of TWOB sites: diverged TWOBs near genes with divergent expression and conserved TWOBs near genes with conserved expression. Consistent with our hypothesis, we observed a larger difference in α values between these two subgroups than between all conserved and diverged TWOBs (Figures S16 and S17).

### CTCF Binding Evolution Is Correlated with the Origin of New Genes

CTCF binding sites in *Drosophila* have been associated with syntenic break points, consistent with their role in delineating the regulatory architecture of genes [13]. We wished to determine whether CTCF binding evolution correlates with any other genome structural evolution. New genes are defined as genes recently originated in a clade, and they provide the opportunity to add new functions to a genome [54]. We found that among 42 young genes that are essential for *Drosophila melanogaster* survival [41], eight show the origin of new CTCF binding sites within 5 kb flanking regions. All eight show phylogenetic correspondence between the appearance of newly evolved CTCF binding sites and the appearance of the associated new gene. Seven new genes exhibit a new CTCF binding site near their 3′ end. For example, *TFII-A-S2* (CG11639) [41] is a newly originated gene through gene duplication in the *melanogaster* subgroup, and a CTCF binding site is observed near its 3′ end in *D. melanogaster*, *D. simulans*, and *D. yakuba* but not in *D. pseudoobscura* (Figure 4C). A similar example is the gene *CheB93a* (CG15503) [41], which originated before the split of *melanogaste*r subgroup from *D. ananasae* as a tandem duplicate of its parental gene *CheB93b* (CG31438) (Figure 4D). We also found a CTCF binding site near the 3′ end of the RNA gene *sphinx* (CR34154) [55], which originated in the *D. melanogaster* branch and is implicated in courtship behavior of male flies [56]. The association of newly evolved essential genes with newly evolved CTCF binding sites is highly significant compared to old essential genes with conserved CTCF binding sites (Kolmogorov-Smirnov test, *p*<1e-6; Figure S19).

How are new binding sites generated? Point mutations are observed in numerous case studies linking *cis*-regulatory sequence change to phenotypic consequences [3] and therefore are considered as the main source of binding site evolution in many theoretical works [57]–[59]. In a mammalian CTCF comparative study, Schmidt et al. presented compelling evidence that CTCF binding sites are driven by retrotransposon expansions, especially in the rodent lineages [9]. We investigated whether TEs might also be associated with CTCF binding site evolution in *Drosophila*. For all the CTCF binding sites identified in *D. melanogaster*, only slightly more than 1% (27 out of 2,267) overlap with annotated TEs [60]. This rate is extremely low compared to rodent species in which around 20% of CTCF binding sits are contained within SINE elements [9], indicating that it is not the primary mode of generating binding site diversity in *Drosophila*. However, for the *D. melanogaster* lineage-specific binding sites, approximately 6% (15 out of 261) of these sites overlap with a TE, resulting in a significant excess of new binding sites overlapping with TEs (Fisher's exact test, *p*<0.0001; Table S18). Thus it is possible that a minority of newly arisen CTCF binding sites have resulted from TE insertions, but the majority of new binding sites are likely originating through mutation selection processes at the nucleotide level.

## Discussion

Ever since King and Wilson proposed the importance of gene regulation for phenotypic variation [2], evolution of *cis*-regulatory elements has been under intensive investigation with an emphasis on enhancers [61],[62] and transcription factor binding sites [4]–[8]. Insulator elements are a special class of *cis-*elements implicated in many fundamental biological processes including transcriptional regulation [14],[15]. Despite their functional importance, the origin and evolution of insulator complexes remained largely uncharted [63],[64]. Only very recently was the first comparative ChIP-seq study on CTCF in mammalian species published [9]. Here, we presented a formal evolutionary genetic analysis of CTCF-related insulator elements in multiple *Drosophila* species.

We found that CTCF binding is highly evolutionarily dynamic, with about 70% of binding events diverged between *D. melanogaster* and *D. pseudoobscura*. This high level of evolutionary divergence is consistent with a recent mammalian study, in which the CTCF binding conservation between human and mouse was estimated to be around 30% [9]. While in mammalian species, CTCF binding profiles are more conserved than tissue-specific transcription factors [7],[9]; in *Drosophila* species, we observe higher binding divergence of CTCF than the developmental transcription factor Twist [8]. In fact, the high degree of binding divergence observed in liver-specific transcription factor CEBPA and HNF4A has led to a proposal of neutral drift underlying binding evolution [7]. However, the population genetic analysis of binding divergence of both the Twist data [8] and our CTCF data indicates that both purifying and positive selection are active forces in CTCF binding evolution. Although previous studies on *Drosophila* noncoding DNA [46],[65] and DNA foot-printing-derived TFBS sequences [66] have suggested the role of positive selection, here we present the first genome-wide evidence in support of positive selection using protein-binding-associated DNA mapped in vivo.

Our observation that young binding sites exhibit a signature of positive selection mimics the pattern observed with young genes [41], indicating that the origination of new binding sites is driven by positive selection. Further, the association between CTCF binding divergence and gene expression divergence indicates that change in CTCF binding has functional consequence. The fact that CTCF binding origination in multiple species coincided with new gene appearance also reinforces this functional view of binding change. The binding changes of this insulator protein may well result in regulatory rewiring through structurally redefining regulatory domains. We predict that this might be a universal mechanism in *cis*-regulatory evolution since CTCF protein is highly conserved across the metazoans [64]. Indeed, in mammalian species, lineage-specific CTCF binding sites are observed to demarcate both chromatin and gene expression domains [9]. Consistent also with the functional relevance of evolutionary changes in CTCF binding profiles, we observed that old and conserved CTCF binding sites are subject to stronger purifying selection and that expression levels of genes near these conserved sites are less likely to diverge. Together these observations indicate that functional constraints maintain conserved binding. This meshes well with the study on Twist [8], in which He et al. found that the most developmentally important genes in early embryo development have the most conserved Twist binding. In summary, we have provided evidence that the evolution of CTCF binding in *Drosophila* species is adaptive.

## Materials and Methods

### Genomic Data Production

The sequenced strains of *D. melanogaster*, *D. simulans*, *D. yakuba*, and *D. pseudoobscura* were maintained at room temperature (18–20°C). Whole animal white prepupa (WPP) for both ChIP-seq and expression profiling experiments were collected strictly within a 15-min time interval to ensure developmentally synchronized samples across species.

Triplicate CTCF ChIP-seq experiments in different species were carried out using a previously published [23] and verified CTCF antibody (Figure S1) according to the standard *Drosophila* modEncode ChIP protocol (www.modencode.org) and Illumina sequencing library preparation protocol. Illumina sequencing data were generated at the High-Throughput Genomic Analysis Core (HGAC) at the Institute for Genomics and Systems Biology.

RNA samples were isolated using Trizol, and the integrity of these samples were checked using an Agilent Bioanalyzer. Transcript levels of *D. melanogaster*, *D. simulans*, and *D. yakuba* WPP samples were measured by single-end mRNA-seq performed in triplicate. Additional sets of quadruplicate expression profiling of *D. melanogaster* and *D. simulans* WPP samples were performed using custom-designed high-density 105K Agilent Gene Expression Arrays. All the genomic data are deposited at GEO under accession number GSE 24449.

### Peak Calling

Sequence reads were mapped back to the genome with the ELAND algorithm using the following Flybase reference genome versions: *D. melanogaster* r5.3, *D. simulans* r1.2, *D. yakuba* r1.2, and *D. pseudoobscura* r2.2. Any reads with more than two mismatches or more than two “N”s were filtered out; only uniquely mapped reads were used in our later analyses. The raw data wiggle files were generated by counting the number of times each coordinate was sequenced. We used the Affymetrix Integrated Genome Browser (IGB) as well as the Broad Institute Integrative Genomics Viewer (IGV) [67] to visualize the raw data and generate snapshots at various genomic positions, as shown in Figure S2.

We modified the peak calling software QuEST [39] to incorporate triplicate data for CTCF binding peak identification. Briefly, QuEST (version 2.0) was used to generate the CDP (Compiled Density Profile) scores for each paired ChIP-input samples. We then normalized the CDP scores by multiplying corresponding ratio scores generated according to sequencing depth, and for each species, we calculated the mean CDP enrichment score (defined as the mean CDP score of ChIP samples minus mean CDP score of input samples) and mean CDP fold enrichment score (defined as the ratio of mean CDP of ChIP samples over mean CDP score of input samples). We performed a permutation-simulation procedure to empirically find the threshold values for the mean CDP enrichment score. We first permutated the experimental label (“ChIP” or “input”) of the CDP scores and then randomly sampled 10,000 coordinates to obtain their mean CDP enrichment score; we repeated the process 100 times and built a “Null” distribution for mean CDP enrichment scores. The 99th percentile of the positive values of the distribution is taken as our threshold for peak calling, which ensures an FDR<1%. We performed peak segmentation using the threshold in a way similar to TAS (Tiling Analysis Software, Affymetrix). We identified regions with at least 50 bp above the threshold and merged neighboring regions if the distance in between is less than 100 bp. We then filtered out peak regions for which the summit coordinate had a mean CDP fold enrichment score of less than 2. We have also calculated, for each identified peak region, the q value (Poison *p* value after multiple testing correction) associated with read number enrichment between ChIP and input samples for each species using raw read count data, and all q values<0.001.

The summit peak coordinates of each identified peak regions were used to infer genomic positions of all the binding events. We designated a CTCF binding event/site as “intronic” or “exonic” if the summit coordinate is within boundaries of an annotated intron or exon, respectively. The remaining binding events/sites were then categorized into “promoter” or “intergenic” groups based on the distance of the peak summit coordinate to the nearest gene transcription start site (TSS): if the distance is <1 kb, we labeled it as “promoter”; otherwise, “ intergenic.”

### Motif Analyses

Species-specific motifs were de novo generated by running MEME [68] on binding site DNA sequences (i.e., 201 bp sequence surrounding the summit coordinate) using default parameters except for setting a motif length of 9 bp. We have used both the top 2,000 and total binding site sequences to run MEME and obtained similar species-specific motifs. FIMO [69] were used to search for motif occurrences in DNA sequences, and a Perl script was written to parse the FIMO result to get percentages of motif containing at various *p* value thresholds and to find the best motif and each individual motif in each binding site.

### Binding Divergence Analyses

#### 1. Direct comparison of identified binding regions in each species

We first mapped all the non–*D. melanogaster* species binding regions into the *D. melanogaster* genome using LiftOver [70], with all default parameters except a match of 0.5; then, we counted the percentage of *D. melanogaster* binding regions overlapping with each of the non–*D. melanogaster* LiftOver binding regions as pair-wise conservation rate. We also performed the reciprocal procedure by mapping *D. melanogaster* binding regions to each of the non–*D. melanogaster* genomes and estimated the pair-wise binding conservation rate as the percentage of each non–*D. melanogaster* binding region that overlaps with the LiftOver *D. melanogaster* binding regions.

#### 2. The *D. melanogaster*–centric quantitative analysis pipeline

We developed a *D. melanogaster*–centric quantitative analysis pipeline to partition read count data variation within and between species and to directly identify conserved and diverged peaks between each pair of the non–*D. melanogaster* species and *D. melanogaster* (*D. simulans*/*D. melanogaster*, *D. yakuba/D. melanogaster*, *D. pseudoobscura/D. melanogaster*). Briefly, we translated all 36 bp uniquely mapped sequence reads in non–*D. melanogaster* (i.e., *D. simulans*, *D. yakuba*, and *D. pseudoobscura*) species into *D. melanogaster* genome using LiftOver [70] with all default parameters, except a match of 0.5. We then generated CDP scores using the LiftOver sequence reads for each non–*D. melanogaster* species using QuEST (version 2.0). For each species pair considered, the CDP scores from each sample were then normalized by multiplying a normalizing value calculated as follows:
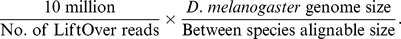



An ANOVA-like linear categorical model described below was then applied to the normalized CDP scores at each coordinate to obtain species-specific ChIP enrichment score estimates, difference of species-specific ChIP enrichment score estimates (the interaction term), and their associated *p* values.
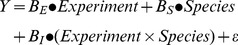
In the model, Y is defined as the observed sequence data, by inputting the normalized CDP scores transformed from sequence read count data of the two species at a specific genomic coordinate. *Experiment* here is a categorical variable (dummy variable) indicating the CDP score source as ChIP or input; *Species* is the other categorical variable, indicating the species source of the CDP score (either *D. melanogaster* or *non*–*D. melanogaster*); *Experiment×Species* is the interaction term between experiment types and species types. *B_E_*, *B_s_*, and *B_I_* are the associated coefficients with the variables to be estimated, and *ε* is the residual error term.

We then smoothed these tracks of ChIP enrichment score estimates as well as the −log10 transformed *p* values for each chromosome by averaging 100 bp sliding windows with each step moving 1 bp. We first identified candidate regions of ChIP enrichments in both species. We then directly identified *D. melanogaster*–specific binding sites, non–*D. melanogaster*–specific binding sites, and shared binding sites. For a binding peak to be identified as shared, it must satisfy the following two requirements: (1) the ChIP enrichment scores in both species are above the threshold and (2) the *p* values associated with ChIP input comparison are significant (−log10 transformed *p* value>20). We took a conservative approach in identifying diverged binding sites. For a binding peak to be identified as *D. melanogaster*–specific, it must satisfy the following three conditions: (1) the ChIP enrichment scores in *D. melanogaster* are above the chosen threshold (see below and Table S5), but in the non–*D. melanogaster* species, the score must be below the threshold; (2) the *p* value associated with the ChIP/input comparison must be significant; and (3) the *p* value associated with the species-specific ChIP effect must be significant. We similarly identified the non–*D. melanogaster*–specific binding sites. We have used a set of different thresholds (0.35, 0.4, 0.5, and 1.0; also see Tables S5 and S6) to identify the diverged and conserved binding sites. The results presented in the main text are based on the threshold 0.4, which is empirically determined in the permutation-simulation procedure for *D. melanogaster* data as described in the section “Peak Calling.” The reason to use this threshold are (1) it is empirically determined and (2) all the non–*D. melanogaster* data have been translated and normalized to be comparable to the *D. melanogaster* data. We applied the other thresholds to test the robustness of observed patterns under looser criteria (0.35) and more stringent criteria (0.5 and 1.0).

Using data simulation, we estimated the False Positive Rate (the rate of identifying conserved binding sites as diverged) of the linear model as 0.35%. The simulation was performed by pooling all the *D. melanogaster* ChIP sample sequence reads together and randomly sampling the same number of reads for each ChIP replicate from the pool to build a simulated ChIP sequence read data set. Similarly, we obtained a simulated input data set. We performed our analysis pipeline with the *D. melanogaster* data and the simulated data. Ideally, we would identify all the binding sites as shared between *D. melanogaster* and the simulated data but found 0.35% of them are identified as diverged.

As an alternative method, we also estimated the overall error rate (False Positive Rate plus False Negative Rate) for misidentifying the pair-wise evolutionary status of *D. melanogaster* binding sites of the whole analysis pipeline as <2% using a set of 100 randomly sampled CTCF binding sites that were manually curated as a “gold standard.” Briefly, we curated 100 random *D. melanogaster* binding peaks by manually inspecting the raw data wiggle file. We then looked at the corresponding othologous regions as well as 2 kb flanking region of the orthologous sequences in each non–*D. melanogaster* species. If we identified any peak using this method, we defined the *D. melanogaster* binding peak as shared, and otherwise, not shared. The percentage of discrepancy between human eye curation of raw count data and our analysis pipeline are taken as the overall error rate. From the pipeline with the empirical thresholds, we identified 2,267 binding sites for *D. melanogaster*, which shows >95% overlap with the binding sites identified previously using triplicate data. We estimated pair-wise binding divergence as the percentage of *D. melanogaster* binding sites that is not shared with the other species.

#### 3. The He et al. method

We followed the method as described in [8]. Briefly, we randomly picked two out of our three replicates for each species to match the structure of analysis He et al. performed. Since there are more input reads than ChIP reads in our data, we performed random sampling of input reads to match the number of reads in the paired ChIP samples. This is important to gain an accurate estimate of FDR with software MACS [71]. We then applied MACS (version 3.2) to identify binding peaks with the D. *melanogaster* sequence read data as well as the non–*D. melanogaster* LiftOver sequence read data. We took the set of binding peaks with a *p* value<10^−21.8^ (same *p* value as He et al. used) in one *D. melanogaster* replicate as the reference binding sites and compared it to all binding sites identified in other species with *p* value cutoff 10^−5^. In order to assess the False Negative Rate, we have also generated two pseudo-ChIP replicates by randomly sampling input sequence reads and performed the same procedure as for other species data.

### Population Genetics Analyses

We downloaded the pre-assembled genome sequences of 37 North American RAL lines from the Drosophila Population Genome Project (www.dpgp.org; Release 1, 50 genome) and filtered out any nucleotide with Phred score <30 as “N.” Combining that data and the *D. melanogaster* reference genome sequence, we generated the polymorphism data for various sets of genomic sites. We included two different types of CTCF-related genomic sites in our analyses: CTCF-201 bp sites and CTCF-motif sites. The CTCF-201 bp sites comprised all the 201 bp flanking sequences centered at the *D. melanogaster* peak coordinate identified in our linear categorical model. The CTCF-motif sites comprised all 9 bp motif sequences found by FIMO at a *p* value = 0.01 within each CTCF-201 bp site concatenated together. The different genomic reference sites were generated by random simulation. For neutral reference, we used the synonymous sites of the nearest genes to the binding sites as well as a set of small intron sequences. The small intron sequences are the 8^th^–30^th^ nucleotides of introns <65 bp as described in [49], and any of these introns overlapping with known EST were filtered out.

We calculated Tajima's D values [44] for different sets of noncoding sites using DnaSP 5.0 batch mode [72] and used Polymorphorama [65] for synonymous and nonsynonymous sites of the nearest genes.

For α estimation, corresponding orthologous DNA sequences in out-group species *D. simulans* and *D. yakuba* were used. Orthologous coding sequences of genes were obtained according to the Flybase (www.flybase.org) annotation. Orthologous sequences for noncoding sites were generated using UCSC pair-wise genome alignment [73]. Sequence alignments were performed with ClustalW2 [74]. The number of polymorphic and divergent sites for noncoding sequences as well as synonymous sites of nearest genes [45],[46] were obtained using a Perl script implementing the PopGen module of BioPerl, which yielded the same result as DnaSP5.0. By taking binding-associated DNA as “nonsynonymous” sites, we estimated the shared α with a 95% confidence interval using DoFE 2.0 [47].

We used the multilocus HKA test [50] C code implemented by Jude Hey (http://genfaculty.rutgers.edu/hey/software#HKA) to perform the HKA tests for the following three comparisons: (1) young CTCF-201 sites versus old CTCF-201 sites; (2) young CTCF-201 sites versus neutral small intron sites; and (3) old CTCF-201 sites versus neutral small intron sites. The sum of deviations is calculated by summing up across all loci, and the *p* values are obtained from 1,000 times of coalescence simulations.

### Expression Data Analyses

For microarray data, probe intensities were extracted using Feature Extraction Software (Agilent). All arrays passed the manufacture's QC and our additional QC processes, with high linear correlation between probe intensities and actual concentration of Spike-in RNAs (linear regression slope ≈1 and *R*-squared >0.95) and high correlation between duplicated probes (Pearson's correlation *r*>0.98). Any probes flagged by FE were treated as missing data. Background subtraction (“normexp” function in “marray” package), log2 transformation, and quantile normalization were performed for each species-specific array set using Bioconductor packages. We took the advantage of the fixed amount of starting Spike-In RNA species in our sample prep experiments and performed the between-species normalization as follows: for each species, we regressed log2 transformed expression measurements of Spike-In probes to the log2 transformed actual RNA concentrations to obtain a regression line; we then subtracted the value of *y*-axis intercept from each probes. We pooled all probes for each pair of orthologs and applied a linear mixture model as follows and categorized the expression level of the gene as “diverged” or “stable” according to the *p* value associated with the estimated between-species expression difference. Correction for multiple testing was performed in a FDR approach [75],[76] using R package “qvalue.”
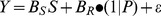
We input *Y* as the normalized log2 microarray intensity measurements of a given pair of orthologs between species; *S* here is a categorical variable indicating the different species (*D. melanogaster* or *D. simulans*); *P* here is a numerical variable indicating the number of different probes for the genes in microarray design; (1|*P*) here indicates the random effects of different probes. *B_S_* and *B_R_* are the coefficients to be estimated. *ε* is the residual error term.

### RNA-seq Data Analyses

For RNA-seq data, we used Bowtie [77] to map the Illumina sequence reads to the genome as well as the annotated exon–exon conjunctions. The number of mapped reads for each gene in different species is counted the same way as described in [78]. Reproducibility between replicates was assessed by calculating the Spearman's rank correlation coefficient of RPM (reads per million) values. For each species pair, we pooled the read count data for orthologous genes together, performed upper-quantile normalization [79], and filtered out genes with <5 reads mapped as “NA.” The genes with divergent expression between species were then called through a generalized linear model framework as described in [80] with a multiple testing corrected *p* value<0.01 and a log2 fold between species difference >2.

### Association Between Essential Genes and CTCF Binding Sites

We used a set of 42 *D. melanogaster* new essential genes (genes originated in *Drosophila* within 25 Myr) as described in [41] and a set of 2,003 old essential genes (genes originated more than 40 Myr ago) for our analysis. The list of old essential genes is a union of two sets: first, a set of 86 old essential genes identified in an RNAi screen as in [41]; second, a set of 1,948 genes with lethal allele phenotypes reported in previous mutagenesis screen studies obtained from the *Drosophila* Interaction Database (DroID) [81]. For the new essential gene set, we calculated the proportion of genes that have phylogenetically congruent CTCF binding sites within flanking regions of different length. A CTCF binding site is described as phylogenetically congruent to a gene if and only if the binding site distributes in the exactly same branches on the phylogeny as the gene. For the old essential gene set, we performed 1,000 times of random sampling; each time we randomly picked 42 genes and performed the same procedure as described for the new essential gene and calculated the mean proportion of old genes that have phylogenetically congruent CTCF binding sites within various flanking regions.

### Overlapping with TEs

We downloaded the annotated TEs in *D. melanogaster* from Flybase and calculated the overlap between the 201 bp flanking region of each group of *D. melanogaster* binding sites with the annotated TEs.

## Supporting Information

Figure S1 Verification of antibody. (A) Alignment of CTCF protein C terminus sequences in the four species. The CTCF_C rabbit antibody used in this article was generated using the *D. melanogaster* CTCF protein C terminus sequence as antigen. The C terminus parts of CTCF protein are identical in the *melanogaster* subgroup species. While there are three amino acid changes between *D. melanogaster* and *D. pseudoobscura*, two of them are similar amino acid changes (in blue) and only one is a different amino acid change (in red). (B) Western Blot of CTCF-C antibody used for the ChIP-seq experiments with *D. pseudoobscura* white pre-pupae extracts at two different volumes. The size of detected band is consistent with the predicted 91.31 kD molecular weight for *D. pseudoobscura* CTCF protein.
(PDF)

Figure S2 CTCF binding profiles at the Bithorax complex region in *D. melanogaster* genome. Previous reported canonical CTCF binding sites in the *Bithorax complex* region are recapitulated in every biological replicate in our ChIP-seq data. From top to bottom, the heights of the wiggle files denote the absolute values of raw data sequence depth for every 10 bp bin calculated using only the uniquely mapped Solexa reads for each of the three ChIP samples—*D. mel* ChIP1, *D. mel* ChIP2, and *D. mel* ChIP3—and their corresponding reference samples—*D. mel* input1, *D. mel* input2, and *D. mel* input3. The seventh panel shows the boundaries of previously identified insulator elements (in sky blue) in this region.
(PDF)

Figure S3 Illustration of the modified QuEST peak calling procedure.
(PDF)

Figure S4 Spearman's rank correlation between ChIP-seq replicates. The Spearman's correlation coefficients (rank order correlation) were calculated with CDP scores (compiled density profile, a QuEST transformation of the sequence depth data for peak calling) in the 500 bp flanking region around the peak summit coordinate for each individual binding peak between any two replicates. The box plots show the overall distribution of Spearman's correlation coefficients for summarized overall combinations of replicate pairs identified in (A) *D. melanogaster*, (B) *D. simulans*, (C) *D. yakuba*, and (D) *D. pseudoobscura*.
(PDF)

Figure S5 Sequence alignments of CTCF protein DNA binding domain (DBD) in the four species. The 11 yellow colored blocks represent the 11 predicted C2H2 zinc finger domains using online domain finding software provided by the Pfam database. Different color depicts different types of amino acids compared to the consensus ones: amino acids that are identical to the consensus (in black); amino acids that are different but with similar properties to the consensus (in blue); and amino acids that are different and have different properties from the consensus (in red).
(PDF)

Figure S6 Illustration of the *D. melanogaster*–centric quantitative analysis pipeline.
(PDF)

Figure S7 Evolutionary groups of CTCF binding events in *D. melanogaster* group. Top panel, representative dynamic binding profiles across the three *D. melanogaster* group species illustrating examples of the seven mutually exclusive binding statuses. The heights of the binding curve denote the ChIP CDP enrichment score estimated from our analysis pipeline (Figure S6). The *y*-axes in the three binding curves for each evolutionary group are at the same scale. In the lower table, the first row contains the Boolean conservation score for each evolutionary status, where 1 depicts the existence of the binding event and 0 depicts the absence of binding event; second and third rows, number of binding events falling into each evolutionary group for all possible binding events and FWOBs (four-way orthologous binding).
(PDF)

Figure S8 Sequence conservation of CTCF binding sites. (A) Distributions of median PhastCons scores for CTCF binding sites. The box plots show the distribution of median PhastCons scores for the conserved and diverged 201 bp sites summarized over all three pair-wise comparisons. (B) Percentage of sequence identity for CTCF binding sites. The box plots show the distribution of percentages of sequence identity in the TWOB 201 bp sites summarized over all three pair-wise comparisons. The percentages of sequence identity are calculated using the pair-wise sequence alignments of the 201 bp flanking sequences of the summit coordinates.
(PDF)

Figure S9 Mean Tajima's D for CTCF-201 sites. Mean Tajima's D values were calculated using 37 *D. melanogaster* North American strains' polymorphism data for various groups of CTCF-201 sites. The center of each circle depicts the mean value, with the error bar indicating 2 standard deviations. The out-group species used here is *D. simulans*. Label abbreviations: Syn/Nonsyn, synonymous/nonsynonymous site of the nearest genes; inter, randomly sampled 201 bp intergenic regions; 3UTR, randomly sampled 201 bp 3′UTR regions; 5UTR, randomly sampled 201 bp 5′UTR regions; TWOB, CTCF-201 bp sites associated with two-way orthologous binding events between *D. melanogaster* and the out-group; conserved TWOB, sites associated with conserved two-way orthologous binding; diverged TWOB, sites associated with diverged two-way orthologous binding; FWOB, sites associated with four-way orthologous binding; Young FWOB, sites associated with those FWOB with inferred evolutionary age <2.5 Myr; Old FWOB, sites associated with those FWOB with inferred evolutionary age >6 Myr.
(PDF)

Figure S10 Mean Tajima's D for CTCF-motif and CTCF-201 high-sequence coverage sites. Mean Tajima's D values for different groups of (A) CTCF-motif and (B) CTCF-201 sites after filtering out sites with input sequence coverage <0.5. The center of each circle depicts the mean value, with error bars indicating 2 standard deviations. The out-group species used here is *D. simulans.* Label abbreviations are the same as for Figure S9.
(PDF)

Figure S11 Shared proportion of adaptation in CTCF-201 bp sites. Shared α values estimated for various groups of CTCF-201 bp sites through the extended MK test framework, with (A) *D. simulans* and (B) *D. yakuba* as out-group species. The center of each circle in the plot depicts the α value estimated, with error bars indicating the 95% confidence interval. The label abbreviations are the same as for Figure S10.
(PDF)

Figure S12 Shared proportion of adaptation in CTCF-motif sites. Shared α values estimated for various groups of CTCF-motif sites through the extended MK test framework with *D. simulans* as an out-group species. The center of each circle in the plot depicts the α value estimated, with error bars indicating the 95% confidence interval. The label abbreviations are the same as for Figure 3.
(PDF)

Figure S13 Shared proportion of adaptation in CTCF-motifs high-sequence coverage sites. Shared α values estimated for various groups of CTCF-motif sites after filtering out sites with input sequence coverage <0.5 through the extended MK test framework, with *D. yakuba* as the out-group species. The center of each circle in the plot depicts the α value estimated, with error bars indicating the 95% confidence interval. The label abbreviations are the same as for Figure 3.
(PDF)

Figure S14 CTCF binding evolution associated with gene expression evolution inferred from microarray data. The bar plots show the proportion of genes with diverged expression between *D. melanogaster*/*D. simulans* associated with different groups of CTCF binding sites: Genome-wide (black), Conserved TWOB (pink), Diverged TWOB (green), Old FWOB (orange), and Young FWOB (light purple). The table below each bar plot shows the number of genes with diverged and conserved gene expression in the corresponding comparisons and associated with the corresponding CTCF binding sites. For each groups of CTCF binding sites, the associated genes are the union of the nearest gene to each binding site. The evolutionary status of gene expression (conserved or diverged) is determined using quadruplicate expression profiling with custom-designed species-specific Agilent 105K microarrays. The label abbreviations are the same as for Figure 3. Significance levels: * *p*<0.05; **p<0.01, one-sided Fisher's exact test.
(PDF)

Figure S15 CTCF binding evolution associated with gene expression evolution inferred from RNA-seq data at high-sequence coverage sites. The bar plots show the proportion of genes with diverged expression between (A) *D. melanogaster/D. simulans* and (B) *D. melanogaster/D. yakuba* comparisons associated with different groups of CTCF binding sites after filtering out sites with input coverage <0.5. All labels are the same as in Figure 4A and 4B.
(PDF)

Figure S16 Shared proportion of adaptation in different groups of CTCF TWOB sites. Shared α values estimated for various groups of (A) CTCF-motif sites and (B) CTCF-201 sites through the extended MK test framework using *D. yakuba* as an out-group. The center of each circle in the plot depicts the α value estimated, with error bars indicating the 95% confidence interval. The label abbreviations: TWOB, Two-Way Orthologous Binding sites identified between *D. melanogaster* and the outgroup species; diverged TWOB, diverged Two-Way Orthologous Binding sites; conserved TWOB, conserved Two-Way Orthologous Binding sites; conserved TWOB with conserved expression, the subset of conserved Two-Way Orthologous Binding sites for which the expression level of their nearest gene are evolutionarily conserved; diverged TWOB with diverged expression, the subset of diverged Two-Way Orthologous Binding sites for which the expression level of their nearest gene are evolutionarily diverged.
(PDF)

Figure S17 Shared proportion of adaptation in different groups of CTCF TWOB high-sequence coverage sites. Shared α values estimated for various groups of (A) CTCF-motif sites and (B) CTCF-201 sites through the extended MK test framework using *D. yakuba* as an out-group. The sites used here for α estimation are those sites with input sequence coverage >0.5. All labels and abbreviations are the same as in Figure S16.
(PDF)

Figure S18 Reproducibility of RNA-seq data. The scatter plot shows the high correlation between gene RPM (number of reads per million) values from two *D. melanogaster* WPP biological samples. The estimated Spearman's rank order correlation is 0.96.
(PDF)

Figure S19 Association between essential genes and CTCF binding events. The red dotted line shows the cumulative proportions of 42 new essential genes (originated less than 25 Myr ago) with phylogenetically congruent CTCF binding sites within flanking regions of various lengths. The blue dotted line shows the cumulative average proportions of randomly sampled 42 old essential genes (originated more than 40 Myr ago) with phylogenetically congruent CTCF binding sites obtained from 1,000 simulations. A CTCF binding site is described as phylogenetically congruent to a gene if and only if the binding event appears in the exactly same branches as the gene on the evolutionary tree. The difference between the two cumulative lines is significant, *p*<1e-6, Komogorov Smirnov test.
(PDF)

Figure S20 Selection signatures in Twist-201 bp sites. (A) Mean Tajima's D values for Twist-201 bp sites. The center of each circle depicts the mean value, with error bars indicating 2 standard deviations. The out-group species used here is *D. yakuba.* (B) Shared proportion of adaptation (alpha) estimated for Twist-201 bp sites using *D. yakuba* as the out-group. The center of each circle in the plot depicts the α value estimated, with error bars indicating the 95% confidence interval. The mean Tajima's D values as well as alpha values for Twist-201 bp sites are plotted together with CTCF-201 bp sites (as labeled in the figure). TWOB, diverged TWOB, and conserved TWOB for Twist and for CTCF are defined the same way as in Figure 3. The TWOB, diverged TWOB, and conserved TWOB Twist binding sites were identified by applying our analysis method to the Twist comparative data.
(PDF)

Table S1 Summary of sequence reads. (A) Number of Solexa sequence reads for ChIP-seq experiments. (B) Number of LiftOver reads of non–*D. melanogaster* species. (C) Number of Solexa sequencing reads for RNA-seq experiments.
(PDF)

Table S2 Ka/Ks ratio for CTCF gene in *Drosophila* species.
(PDF)

Table S3 CTCF binding site motif enrichment in each species.
(PDF)

Table S4 Pearson's correlation coefficients between ChIP replicates.
(PDF)

Table S5 Diverged and conserved CTCF binding events.
(PDF)

Table S6 Diverged and conserved CTCF binding events at high-sequence coverage sites.
(PDF)

Table S7 CTCF binding divergence estimated by direct comparison.
(PDF)

Table S8 CTCF binding divergence estimated using He et al. method.
(PDF)

Table S9 Twist binding divergence estimated using our pipeline.
(PDF)

Table S10 Summary of Twist binding divergence estimated using different methods.
(PDF)

Table S11 Genomic distribution of different evolutionary groups of CTCF binding events.
(PDF)

Table S12 Parsimonious age dating of *D. melanogaster* CTCF binding events.
(PDF)

Table S13 Evolutionary groups of CTCF binding events at high-sequence coverage sites.
(PDF)

Table S14 Number of fixed and polymorphic mutations in CTCF-associated DNA sequences.
(PDF)

Table S15 Number of fixed and polymorphic mutations in CTCF-associated DNA sequences at high-sequence coverage sites.
(PDF)

Table S16 HKA test for old and young sites.
(PDF)

Table S17 HKA test for old and young high-sequence coverage sites.
(PDF)

Table S18 Overlapping of CTCF binding sites with TE.
(PDF)

## Abbreviations

ANOVA: analysis of variance
ChIP: chromatin immunoprecipitation
CTCF: CCCTC binding factor
FWOB: four way orthologous binding
Myr: million years
TE: transposable element
TWOB: two way orthologous binding

## References

[1] Carroll SB (2008) Evo-devo and an expanding evolutionary synthesis: a genetic theory of morphological evolution. Cell 134: 25–36. 18614008 10.1016/j.cell.2008.06.030

[2] King MC, Wilson AC (1975) Evolution at two levels in humans and chimpanzees. Science 188: 107–116. 1090005 10.1126/science.1090005

[3] Wray GA (2007) The evolutionary significance of cis-regulatory mutations. Nat Rev Genet 8: 206–216. 17304246 10.1038/nrg2063

[4] Borneman AR, Gianoulis TA, Zhang ZD, Yu H, Rozowsky J, et al (2007) Divergence of transcription factor binding sites across related yeast species. Science 317: 815–819. 17690298 10.1126/science.1140748

[5] Bradley RK, Li XY, Trapnell C, Davidson S, Pachter L, et al (2010) Binding site turnover produces pervasive quantitative changes in transcription factor binding between closely related Drosophila species. PLoS Biol 8: e1000343 doi:10.1371/journal.pbio.1000343. 20351773 10.1371/journal.pbio.1000343 PMC2843597

[6] Odom DT, Dowell RD, Jacobsen ES, Gordon W, Danford TW, et al (2007) Tissue-specific transcriptional regulation has diverged significantly between human and mouse. Nat Genet 39: 730–732. 17529977 10.1038/ng2047 PMC3797512

[7] Schmidt D, Wilson MD, Ballester B, Schwalie PC, Brown GD, et al (2010) Five-vertebrate ChIP-seq reveals the evolutionary dynamics of transcription factor binding. Science 328: 1036–1040. 20378774 10.1126/science.1186176 PMC3008766

[8] He Q, Bardet AF, Patton B, Purvis J, Johnston J, et al (2011) High conservation of transcription factor binding and evidence for combinatorial regulation across six Drosophila species. Nat Genet 43: 414–420. 21478888 10.1038/ng.808

[9] Schmidt D, Schwalie PC, Wilson MD, Ballester B, Goncalves A, et al (2012) Waves of retrotransposon expansion remodel genome organization and CTCF binding in multiple mammalian lineages. Cell 148: 335–348. 22244452 10.1016/j.cell.2011.11.058 PMC3368268

[10] Geyer PK (1997) The role of insulator elements in defining domains of gene expression. Curr Opin Genet Dev 7: 242–248. 9115431 10.1016/s0959-437x(97)80134-7

[11] Wallace JA, Felsenfeld G (2007) We gather together: insulators and genome organization. Curr Opin Genet Dev 17: 400–407. 17913488 10.1016/j.gde.2007.08.005 PMC2215060

[12] Phillips JE, Corces VG (2009) CTCF: master weaver of the genome. Cell 137: 1194–1211. 19563753 10.1016/j.cell.2009.06.001 PMC3040116

[13] Negre N, Brown CD, Shah PK, Kheradpour P, Morrison CA, et al (2010) A comprehensive map of insulator elements for the Drosophila genome. PLoS Genet 6: e1000814 doi:10.1371/journal.pgen.1000814. 20084099 10.1371/journal.pgen.1000814 PMC2797089

[14] Gaszner M, Felsenfeld G (2006) Insulators: exploiting transcriptional and epigenetic mechanisms. Nat Rev Genet 7: 703–713. 16909129 10.1038/nrg1925

[15] Valenzuela L, Kamakaka RT (2006) Chromatin insulators. Annu Rev Genet 40: 107–138. 16953792 10.1146/annurev.genet.39.073003.113546

[16] Gerasimova TI, Corces VG (2001) Chromatin insulators and boundaries: effects on transcription and nuclear organization. Annu Rev Genet 35: 193–208. 11700282 10.1146/annurev.genet.35.102401.090349

[17] Guelen L, Pagie L, Brasset E, Meuleman W, Faza MB, et al (2008) Domain organization of human chromosomes revealed by mapping of nuclear lamina interactions. Nature 453: 948–951. 18463634 10.1038/nature06947

[18] Cuddapah S, Jothi R, Schones DE, Roh TY, Cui K, et al (2009) Global analysis of the insulator binding protein CTCF in chromatin barrier regions reveals demarcation of active and repressive domains. Genome Res 19: 24–32. 19056695 10.1101/gr.082800.108 PMC2612964

[19] Handoko L, Xu H, Li G, Ngan CY, Chew E, et al (2011) CTCF-mediated functional chromatin interactome in pluripotent cells. Nat Genet 43: 630–638. 21685913 10.1038/ng.857 PMC3436933

[20] Kim TH, Abdullaev ZK, Smith AD, Ching KA, Loukinov DI, et al (2007) Analysis of the vertebrate insulator protein CTCF-binding sites in the human genome. Cell 128: 1231–1245. 17382889 10.1016/j.cell.2006.12.048 PMC2572726

[21] Smith ST, Wickramasinghe P, Olson A, Loukinov D, Lin L, et al (2009) Genome wide ChIP-chip analyses reveal important roles for CTCF in Drosophila genome organization. Dev Biol 328: 518–528. 19210964 10.1016/j.ydbio.2008.12.039 PMC6620017

[22] Bushey AM, Ramos E, Corces VG (2009) Three subclasses of a Drosophila insulator show distinct and cell type-specific genomic distributions. Genes Dev 23: 1338–1350. 19443682 10.1101/gad.1798209 PMC2701583

[23] Holohan EE, Kwong C, Adryan B, Bartkuhn M, Herold M, et al (2007) CTCF genomic binding sites in Drosophila and the organisation of the bithorax complex. PLoS Genet 3: e112 doi:10.1371/journal.pgen.0030112. 17616980 10.1371/journal.pgen.0030112 PMC1904468

[24] Jiang N, Emberly E, Cuvier O, Hart CM (2009) Genome-wide mapping of boundary element-associated factor (BEAF) binding sites in Drosophila melanogaster links BEAF to transcription. Mol Cell Biol 29: 3556–3568. 19380483 10.1128/MCB.01748-08 PMC2698748

[25] Moon H, Filippova G, Loukinov D, Pugacheva E, Chen Q, et al (2005) CTCF is conserved from Drosophila to humans and confers enhancer blocking of the Fab-8 insulator. EMBO Rep 6: 165–170. 15678159 10.1038/sj.embor.7400334 PMC1299244

[26] Murrell A, Heeson S, Reik W (2004) Interaction between differentially methylated regions partitions the imprinted genes Igf2 and H19 into parent-specific chromatin loops. Nat Genet 36: 889–893. 15273689 10.1038/ng1402

[27] Bell AC, Felsenfeld G (2000) Methylation of a CTCF-dependent boundary controls imprinted expression of the Igf2 gene. Nature 405: 482–485. 10839546 10.1038/35013100

[28] Filippova GN, Cheng MK, Moore JM, Truong JP, Hu YJ, et al (2005) Boundaries between chromosomal domains of X inactivation and escape bind CTCF and lack CpG methylation during early development. Dev Cell 8: 31–42. 15669143 10.1016/j.devcel.2004.10.018

[29] Fu VX, Dobosy JR, Desotelle JA, Almassi N, Ewald JA, et al (2008) Aging and cancer-related loss of insulin-like growth factor 2 imprinting in the mouse and human prostate. Cancer Res 68: 6797–6802. 18701505 10.1158/0008-5472.CAN-08-1714 PMC4237281

[30] Kong A, Steinthorsdottir V, Masson G, Thorleifsson G, Sulem P, et al (2009) Parental origin of sequence variants associated with complex diseases. Nature 462: 868–874. 20016592 10.1038/nature08625 PMC3746295

[31] Demars J, Shmela ME, Rossignol S, Okabe J, Netchine I, et al (2010) Analysis of the IGF2/H19 imprinting control region uncovers new genetic defects, including mutations of OCT-binding sequences, in patients with 11p15 fetal growth disorders. Hum Mol Genet 19: 803–814. 20007505 10.1093/hmg/ddp549

[32] McDaniell R, Lee BK, Song L, Liu Z, Boyle AP, et al (2010) Heritable individual-specific and allele-specific chromatin signatures in humans. Science 328: 235–239. 20299549 10.1126/science.1184655 PMC2929018

[33] Karch F, Galloni M, Sipos L, Gausz J, Gyurkovics H, et al (1994) Mcp and Fab-7: molecular analysis of putative boundaries of cis-regulatory domains in the bithorax complex of Drosophila melanogaster. Nucleic Acids Res 22: 3138–3146. 7915032 10.1093/nar/22.15.3138 PMC310287

[34] Mohan M, Bartkuhn M, Herold M, Philippen A, Heinl N, et al (2007) The Drosophila insulator proteins CTCF and CP190 link enhancer blocking to body patterning. EMBO J 26: 4203–4214. 17805343 10.1038/sj.emboj.7601851 PMC2230845

[35] Barges S, Mihaly J, Galloni M, Hagstrom K, Muller M, et al (2000) The Fab-8 boundary defines the distal limit of the bithorax complex iab-7 domain and insulates iab-7 from initiation elements and a PRE in the adjacent iab-8 domain. Development 127: 779–790. 10648236 10.1242/dev.127.4.779

[36] Mihaly J, Barges S, Sipos L, Maeda R, Cleard F, et al (2006) Dissecting the regulatory landscape of the Abd-B gene of the bithorax complex. Development 133: 2983–2993. 16818450 10.1242/dev.02451

[37] Powell JR (1997). Progress and prospects in evolutionary biology: the Drosophila model: Oxford University Press. pp. xiv, 562 p.

[38] White KP, Rifkin SA, Hurban P, Hogness DS (1999) Microarray analysis of Drosophila development during metamorphosis. Science 286: 2179–2184. 10591654 10.1126/science.286.5447.2179

[39] Valouev A, Johnson DS, Sundquist A, Medina C, Anton E, et al (2008) Genome-wide analysis of transcription factor binding sites based on ChIP-Seq data. Nat Methods 5: 829–834. 19160518 10.1038/nmeth.1246 PMC2917543

[40] Clark AG, Eisen MB, Smith DR, Bergman CM, Oliver B, et al (2007) Evolution of genes and genomes on the Drosophila phylogeny. Nature 450: 203–218. 17994087 10.1038/nature06341

[41] Chen S, Zhang YE, Long M (2010) New genes in Drosophila quickly become essential. Science 330: 1682–1685. 21164016 10.1126/science.1196380 PMC7211344

[42] Zhang YE, Vibranovski MD, Krinsky BH, Long M (2010) Age-dependent chromosomal distribution of male-biased genes in Drosophila. Genome Res 20: 1526–1533. 20798392 10.1101/gr.107334.110 PMC2963816

[43] Siepel A, Haussler D (2004) Combining phylogenetic and hidden Markov models in biosequence analysis. J Comput Biol 11: 413–428. 15285899 10.1089/1066527041410472

[44] Tajima F (1989) Statistical method for testing the neutral mutation hypothesis by DNA polymorphism. Genetics 123: 585–595. 2513255 10.1093/genetics/123.3.585 PMC1203831

[45] McDonald JH, Kreitman M (1991) Adaptive protein evolution at the Adh locus in Drosophila. Nature 351: 652–654. 1904993 10.1038/351652a0

[46] Andolfatto P (2005) Adaptive evolution of non-coding DNA in Drosophila. Nature 437: 1149–1152. 16237443 10.1038/nature04107

[47] Bierne N, Eyre-Walker A (2004) The genomic rate of adaptive amino acid substitution in Drosophila. Mol Biol Evol 21: 1350–1360. 15044594 10.1093/molbev/msh134

[48] Powell JR, Moriyama EN (1997) Evolution of codon usage bias in Drosophila. Proc Natl Acad Sci U S A 94: 7784–7790. 9223264 10.1073/pnas.94.15.7784 PMC33704

[49] Parsch J, Novozhilov S, Saminadin-Peter SS, Wong KM, Andolfatto P (2010) On the utility of short intron sequences as a reference for the detection of positive and negative selection in Drosophila. Mol Biol Evol 27: 1226–1234. 20150340 10.1093/molbev/msq046 PMC2877998

[50] Hudson RR, Kreitman M, Aguade M (1987) A test of neutral molecular evolution based on nucleotide data. Genetics 116: 153–159. 3110004 10.1093/genetics/116.1.153 PMC1203113

[51] Rifkin SA, Kim J, White KP (2003) Evolution of gene expression in the Drosophila melanogaster subgroup. Nat Genet 33: 138–144. 12548287 10.1038/ng1086

[52] Rifkin SA, Houle D, Kim J, White KP (2005) A mutation accumulation assay reveals a broad capacity for rapid evolution of gene expression. Nature 438: 220–223. 16281035 10.1038/nature04114

[53] Holloway AK, Lawniczak MK, Mezey JG, Begun DJ, Jones CD (2007) Adaptive gene expression divergence inferred from population genomics. PLoS Genet 3: 2007–2013 doi:10.1371/journal.pgen.0030187. 17967066 10.1371/journal.pgen.0030187 PMC2042001

[54] Long M, Betran E, Thornton K, Wang W (2003) The origin of new genes: glimpses from the young and old. Nat Rev Genet 4: 865–875. 14634634 10.1038/nrg1204

[55] Wang W, Brunet FG, Nevo E, Long M (2002) Origin of sphinx, a young chimeric RNA gene in Drosophila melanogaster. Proc Natl Acad Sci U S A 99: 4448–4453. 11904380 10.1073/pnas.072066399 PMC123668

[56] Dai H, Chen Y, Chen S, Mao Q, Kennedy D, et al (2008) The evolution of courtship behaviors through the origination of a new gene in Drosophila. Proc Natl Acad Sci U S A 105: 7478–7483. 18508971 10.1073/pnas.0800693105 PMC2396706

[57] Carter AJ, Wagner GP (2002) Evolution of functionally conserved enhancers can be accelerated in large populations: a population-genetic model. Proc Biol Sci 269: 953–960. 12028779 10.1098/rspb.2002.1968 PMC1690979

[58] Stone JR, Wray GA (2001) Rapid evolution of cis-regulatory sequences via local point mutations. Mol Biol Evol 18: 1764–1770. 11504856 10.1093/oxfordjournals.molbev.a003964

[59] MacArthur S, Brookfield JF (2004) Expected rates and modes of evolution of enhancer sequences. Mol Biol Evol 21: 1064–1073. 15014138 10.1093/molbev/msh105

[60] Kaminker JS, Bergman CM, Kronmiller B, Carlson J, Svirskas R, et al (2002) The transposable elements of the Drosophila melanogaster euchromatin: a genomics perspective. Genome Biol 3: RESEARCH0084. 12537573 10.1186/gb-2002-3-12-research0084 PMC151186

[61] Ludwig MZ, Bergman C, Patel NH, Kreitman M (2000) Evidence for stabilizing selection in a eukaryotic enhancer element. Nature 403: 564–567. 10676967 10.1038/35000615

[62] Hare EE, Peterson BK, Iyer VN, Meier R, Eisen MB (2008) Sepsid even-skipped enhancers are functionally conserved in Drosophila despite lack of sequence conservation. PLoS Genet 4: e1000106 doi:10.1371/journal.pgen.1000106. 18584029 10.1371/journal.pgen.1000106 PMC2430619

[63] Raab JR, Kamakaka RT (2010) Insulators and promoters: closer than we think. Nat Rev Genet 11: 439–446. 20442713 10.1038/nrg2765 PMC3477808

[64] Heger P, Marin B, Schierenberg E (2009) Loss of the insulator protein CTCF during nematode evolution. BMC Mol Biol 10: 84. 19712444 10.1186/1471-2199-10-84 PMC2749850

[65] Haddrill PR, Bachtrog D, Andolfatto P (2008) Positive and negative selection on noncoding DNA in Drosophila simulans. Mol Biol Evol 25: 1825–1834. 18515263 10.1093/molbev/msn125 PMC2734132

[66] He BZ, Holloway AK, Maerkl SJ, Kreitman M (2011) Does positive selection drive transcription factor binding site turnover? A test with Drosophila cis-regulatory modules. PLoS Genet 7: e1002053 doi:10.1371/journal.pgen.1002053. 21572512 10.1371/journal.pgen.1002053 PMC3084208

[67] Robinson JT, Thorvaldsdottir H, Winckler W, Guttman M, Lander ES, et al (2011) Integrative genomics viewer. Nat Biotechnol 29: 24–26. 21221095 10.1038/nbt.1754 PMC3346182

[68] Bailey TL, Elkan. C (1994) Fitting a mixture model by expectation maximization to discover motifs in biopolymers. Proceedings of the Second International Conference on Intelligent Systems for Molecular Biology. Menlo Park, California: AAAI Press, pp. 28–36. 7584402

[69] Grant CE, Bailey TL, Noble WS (2011) FIMO: scanning for occurrences of a given motif. Bioinformatics 27: 1017–1018. 21330290 10.1093/bioinformatics/btr064 PMC3065696

[70] Kuhn RM, Karolchik D, Zweig AS, Wang T, Smith KE, et al (2009) The UCSC Genome Browser Database: update 2009. Nucleic Acids Res 37: D755–D761. 18996895 10.1093/nar/gkn875 PMC2686463

[71] Zhang Y, Liu T, Meyer CA, Eeckhoute J, Johnson DS, et al (2008) Model-based analysis of ChIP-Seq (MACS). Genome Biol 9: R137. 18798982 10.1186/gb-2008-9-9-r137 PMC2592715

[72] Librado P, Rozas J (2009) DnaSP v5: a software for comprehensive analysis of DNA polymorphism data. Bioinformatics 25: 1451–1452. 19346325 10.1093/bioinformatics/btp187

[73] Chiaromonte F, Yap VB, Miller W (2002) Scoring pairwise genomic sequence alignments. Pac Symp Biocomput 115–126. 11928468 10.1142/9789812799623_0012

[74] Larkin MA, Blackshields G, Brown NP, Chenna R, McGettigan PA, et al (2007) Clustal W and Clustal X version 2.0. Bioinformatics 23: 2947–2948. 17846036 10.1093/bioinformatics/btm404

[75] Storey JD (2002) A direct approach to false discovery rates. Journal of the Royal Statistical Society Series B-Statistical Methodology 64: 479–498.

[76] Storey JD, Tibshirani R (2003) Statistical significance for genomewide studies. Proc Natl Acad Sci U S A 100: 9440–9445. 12883005 10.1073/pnas.1530509100 PMC170937

[77] Langmead B, Trapnell C, Pop M, Salzberg SL (2009) Ultrafast and memory-efficient alignment of short DNA sequences to the human genome. Genome Biol 10: R25. 19261174 10.1186/gb-2009-10-3-r25 PMC2690996

[78] Mortazavi A, Williams BA, McCue K, Schaeffer L, Wold B (2008) Mapping and quantifying mammalian transcriptomes by RNA-Seq. Nat Methods 5: 621–628. 18516045 10.1038/nmeth.1226 PMC13303166

[79] Bullard JH, Purdom E, Hansen KD, Dudoit S (2010) Evaluation of statistical methods for normalization and differential expression in mRNA-Seq experiments. BMC Bioinformatics 11: 94. 20167110 10.1186/1471-2105-11-94 PMC2838869

[80] Marioni JC, Mason CE, Mane SM, Stephens M, Gilad Y (2008) RNA-seq: an assessment of technical reproducibility and comparison with gene expression arrays. Genome Res 18: 1509–1517. 18550803 10.1101/gr.079558.108 PMC2527709

[81] Murali T, Pacifico S, Yu J, Guest S, Roberts GG 3rd, et al (2011) DroID 2011: a comprehensive, integrated resource for protein, transcription factor, RNA and gene interactions for Drosophila. Nucleic Acids Res 39: D736–D743. 21036869 10.1093/nar/gkq1092 PMC3013689

