# Supplementary material for: Adaptive Evolution and the Birth of CTCF Binding Sites in the Drosophila Genome
Source: PLoS Biol. 2012 Nov 6;10(11):e1001420. doi: 10.1371/journal.pbio.1001420 (PMC3491045; doi:10.1371/journal.pbio.1001420)

Figure S1

A

D.mel C terminus	VDGASQEFLQLMDMIEQDS
D.sim C terminus	VDGASQEFLQLMDMIEQDS
D.yak C terminus	VDGASQEFLQLMDMIEQDS
D.pse C terminus	MDGTSQEFLQLMDMIEQDA

B

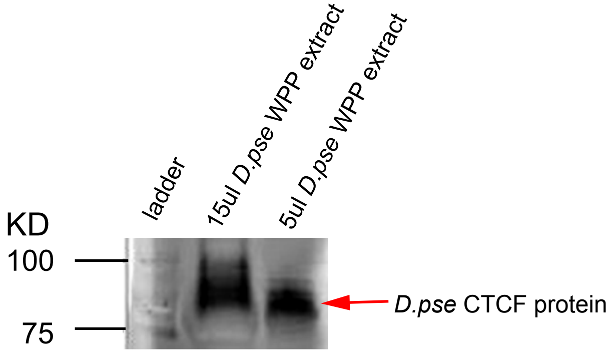

Supplement: Figure S1 — Verification of antibody. (A) Alignment of CTCF protein C terminus sequences in the four species. The CTCF_C rabbit antibody used in this article was generated using the D. melanogaster CTCF protein C terminus sequence as antigen. The C terminus parts of CTCF protein are identical in the melanogaster subgroup species. While there are three amino acid changes between D. melanogaster and D. pseudoobscura, two of them are similar amino acid changes (in blue) and only one is a different amino acid change (in red). (B) Western Blot of CTCF-C antibody used for the ChIP-seq experiments with D. pseudoobscura white pre-pupae extracts at two different volumes. The size of detected band is consistent with the predicted 91.31 kD molecular weight for D. pseudoobscura CTCF protein. (PDF) [file pbio.1001420.s001.pdf]
