# Supplementary figures and images for: Adaptive Evolution and the Birth of CTCF Binding Sites in the Drosophila Genome
Source: PLoS Biol. 2012 Nov 6;10(11):e1001420. doi: 10.1371/journal.pbio.1001420 (PMC3491045; doi:10.1371/journal.pbio.1001420)

Figure S2

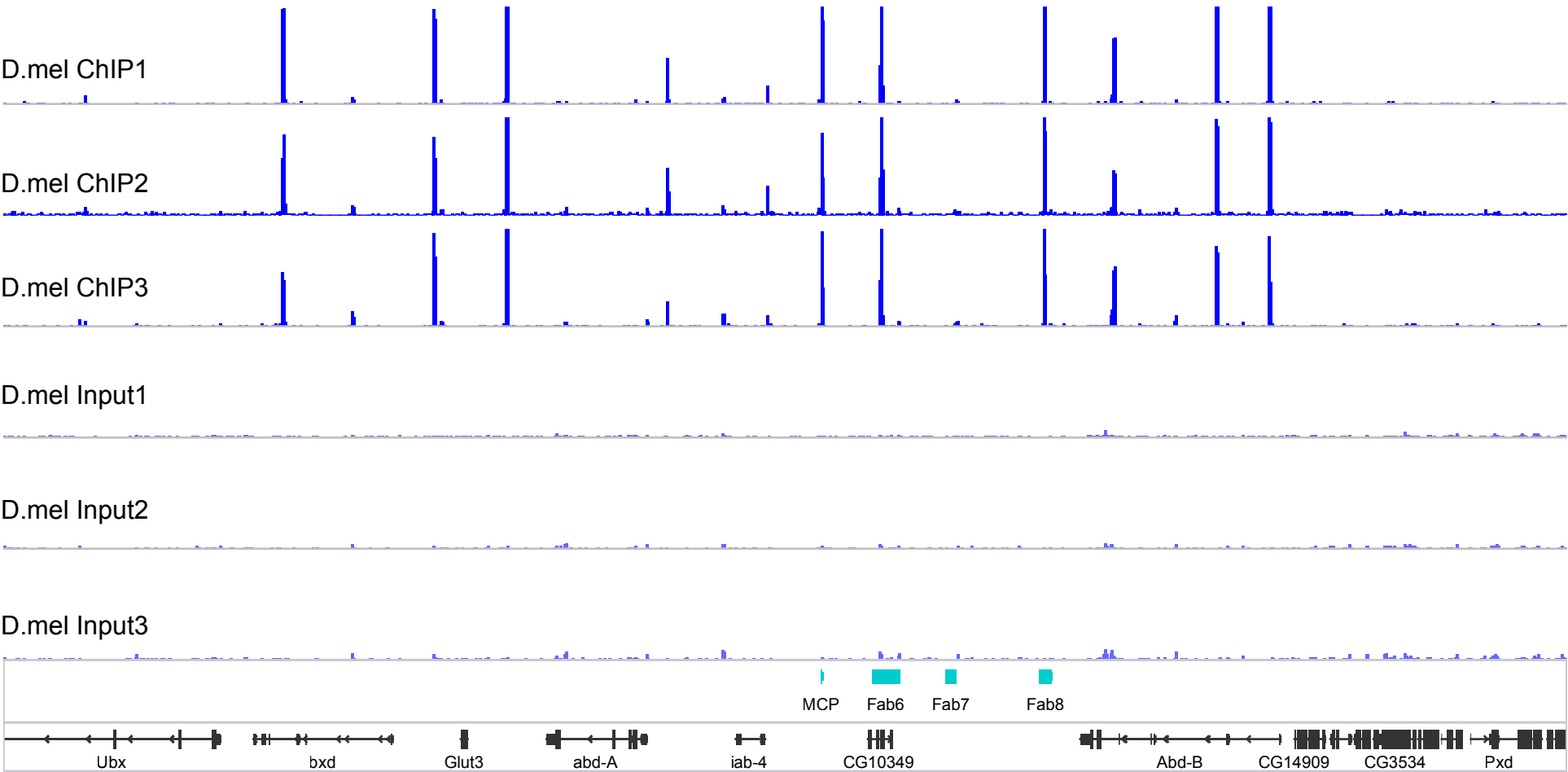

Supplement: Figure S2 — CTCF binding profiles at the Bithorax complex region in D. melanogaster genome. Previous reported canonical CTCF binding sites in the Bithorax complex region are recapitulated in every biological replicate in our ChIP-seq data. From top to bottom, the heights of the wiggle files denote the absolute values of raw data sequence depth for every 10 bp bin calculated using only the uniquely mapped Solexa reads for each of the three ChIP samples—D. mel ChIP1, D. mel ChIP2, and D. mel ChIP3—and their corresponding reference samples—D. mel input1, D. mel input2, and D. mel input3. The seventh panel shows the boundaries of previously identified insulator elements (in sky blue) in this region. (PDF) [file pbio.1001420.s002.pdf]

Figure S3

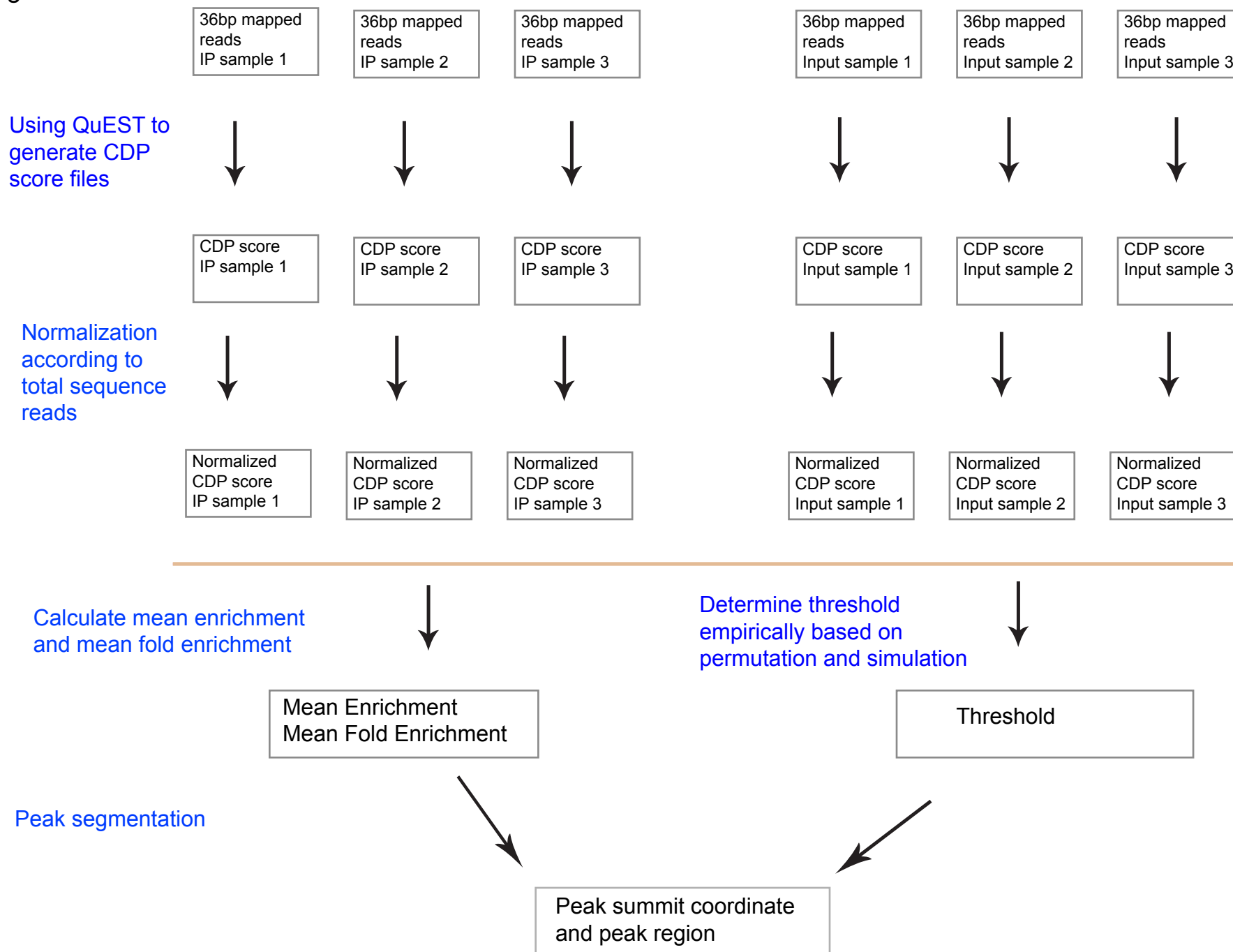

Supplement: Figure S3 — Illustration of the modified QuEST peak calling procedure. (PDF) [file pbio.1001420.s003.pdf]

Figure S6

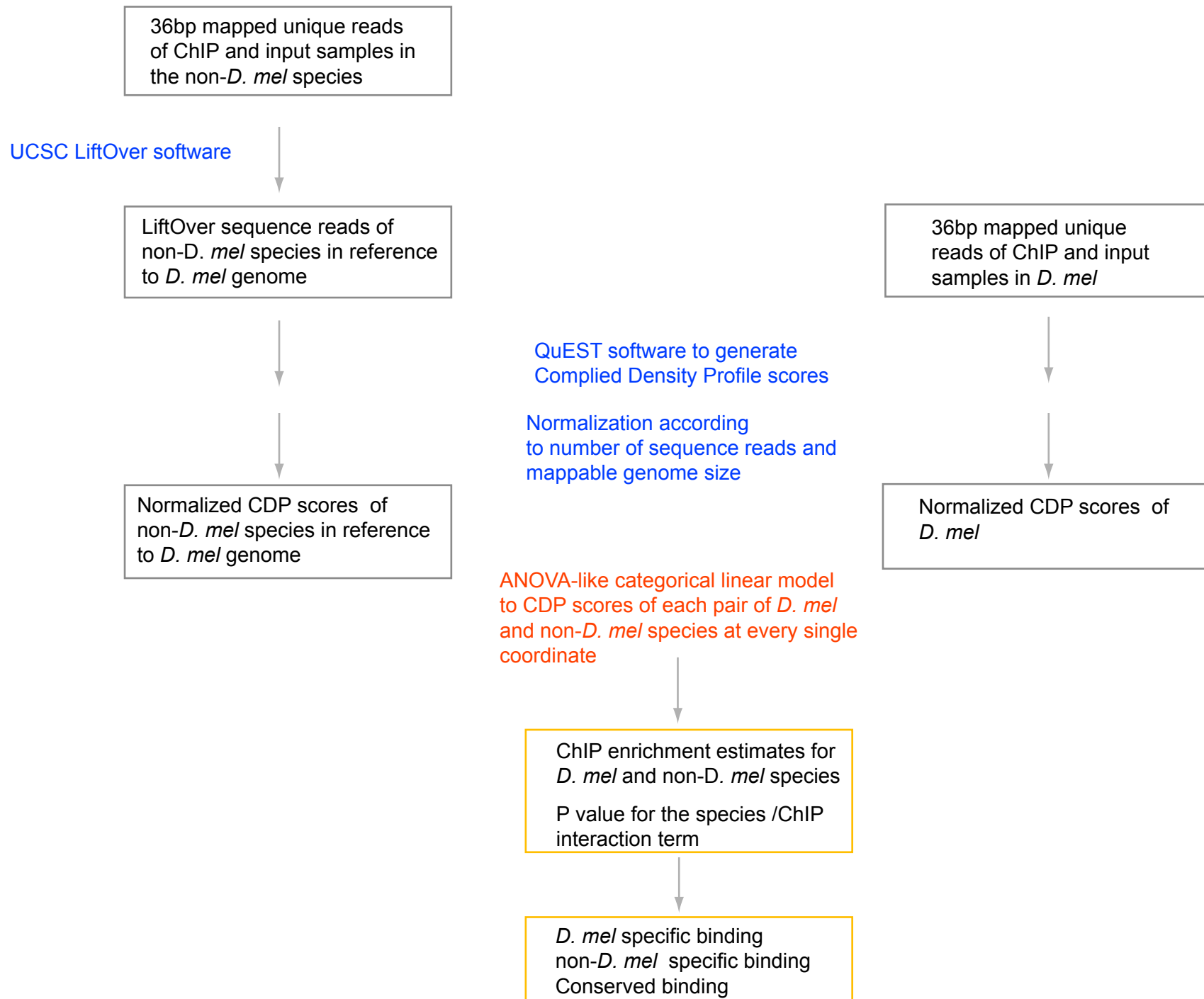

Supplement: Figure S6 — Illustration of the D. melanogaster–centric quantitative analysis pipeline. (PDF) [file pbio.1001420.s006.pdf]

Figure S7

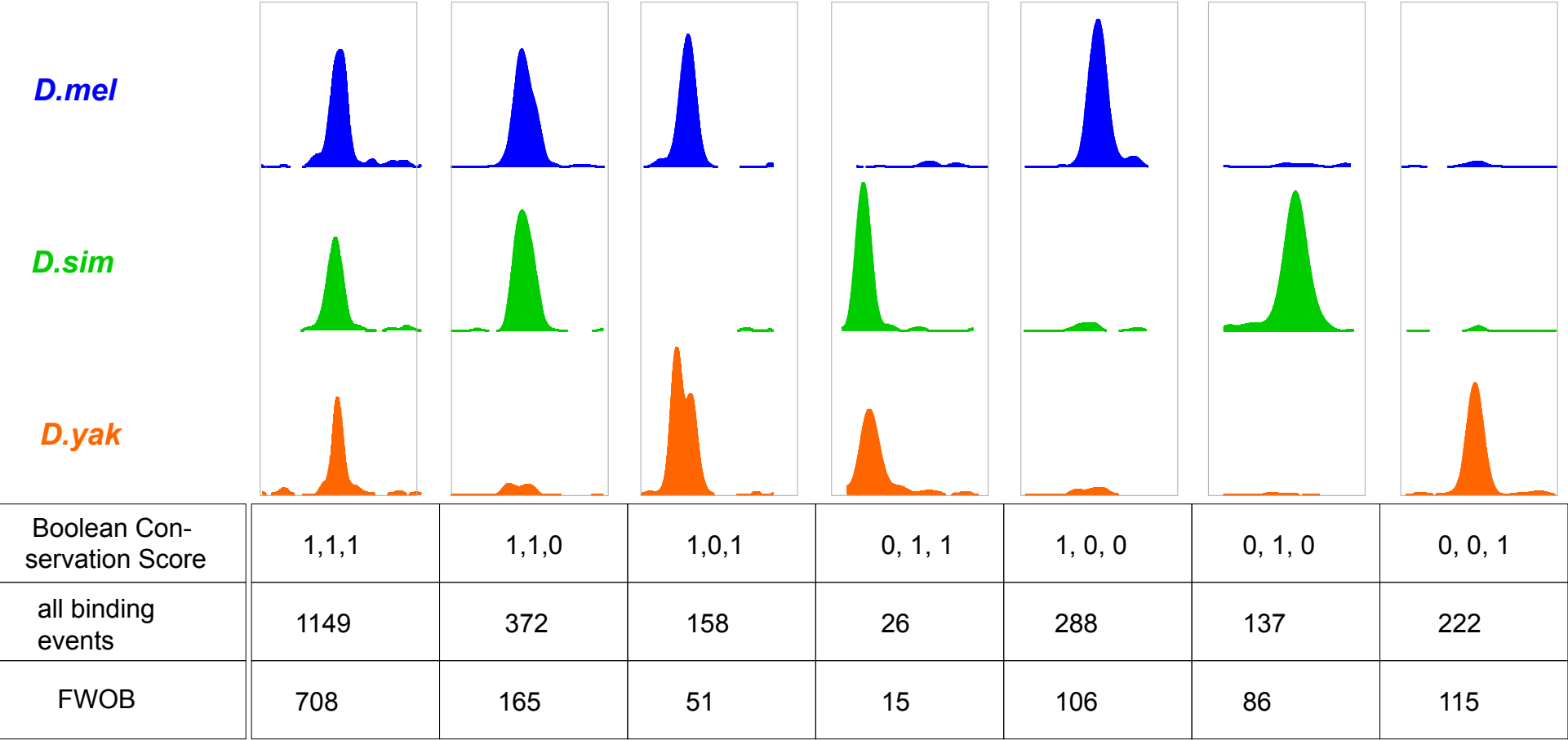

Supplement: Figure S7 — Evolutionary groups of CTCF binding events in D. melanogaster group. Top panel, representative dynamic binding profiles across the three D. melanogaster group species illustrating examples of the seven mutually exclusive binding statuses. The heights of the binding curve denote the ChIP CDP enrichment score estimated from our analysis pipeline (Figure S6). The y-axes in the three binding curves for each evolutionary group are at the same scale. In the lower table, the first row contains the Boolean conservation score for each evolutionary status, where 1 depicts the existence of the binding event and 0 depicts the absence of binding event; second and third rows, number of binding events falling into each evolutionary group for all possible binding events and FWOBs (four-way orthologous binding). (PDF) [file pbio.1001420.s007.pdf]

Figure S8

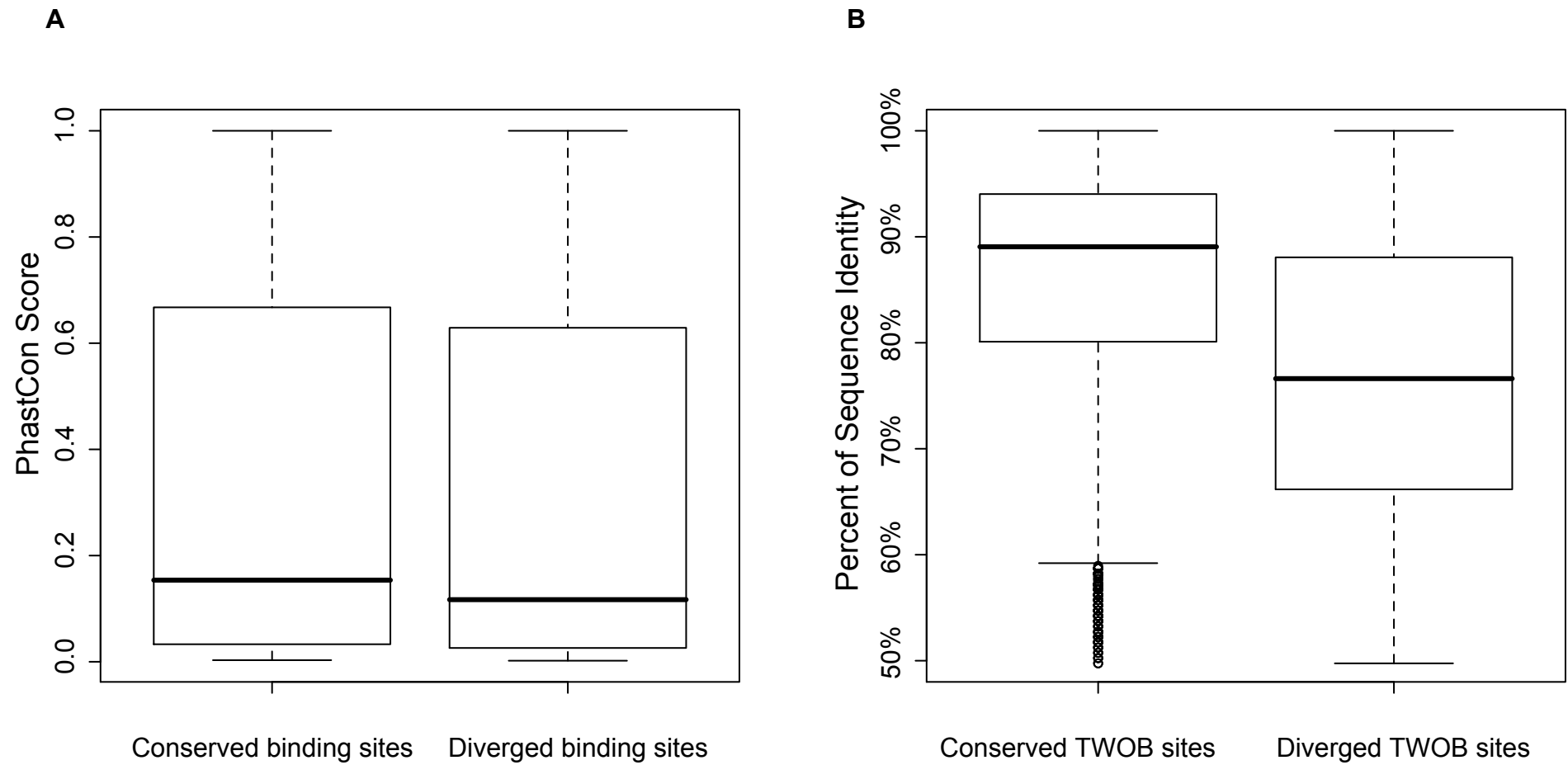

Supplement: Figure S8 — Sequence conservation of CTCF binding sites. (A) Distributions of median PhastCons scores for CTCF binding sites. The box plots show the distribution of median PhastCons scores for the conserved and diverged 201 bp sites summarized over all three pair-wise comparisons. (B) Percentage of sequence identity for CTCF binding sites. The box plots show the distribution of percentages of sequence identity in the TWOB 201 bp sites summarized over all three pair-wise comparisons. The percentages of sequence identity are calculated using the pair-wise sequence alignments of the 201 bp flanking sequences of the summit coordinates. (PDF) [file pbio.1001420.s008.pdf]

Figure S9

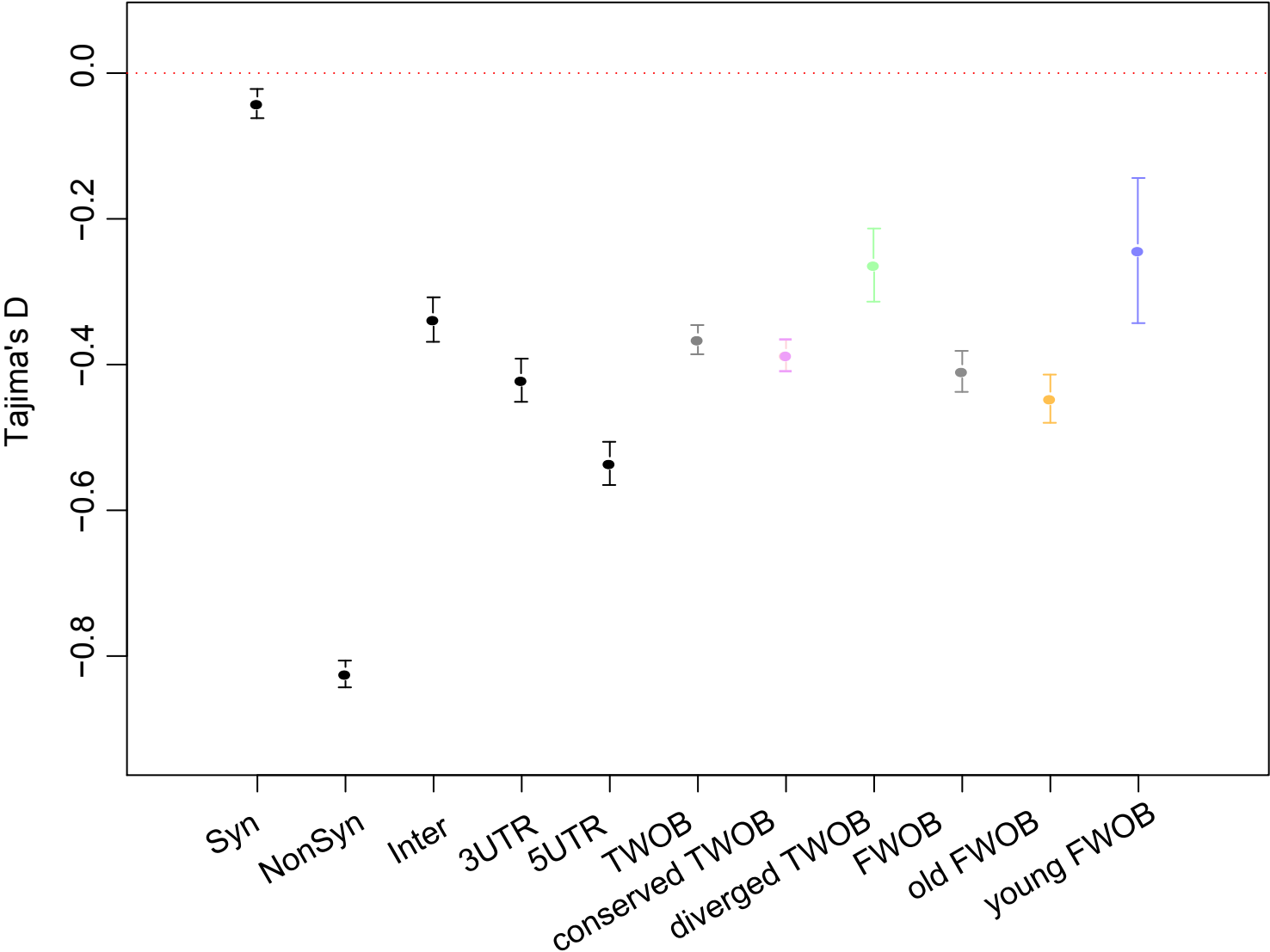

Supplement: Figure S9 — Mean Tajima's D for CTCF-201 sites. Mean Tajima's D values were calculated using 37 D. melanogaster North American strains' polymorphism data for various groups of CTCF-201 sites. The center of each circle depicts the mean value, with the error bar indicating 2 standard deviations. The out-group species used here is D. simulans. Label abbreviations: Syn/Nonsyn, synonymous/nonsynonymous site of the nearest genes; inter, randomly sampled 201 bp intergenic regions; 3UTR, randomly sampled 201 bp 3′UTR regions; 5UTR, randomly sampled 201 bp 5′UTR regions; TWOB, CTCF-201 bp sites associated with two-way orthologous binding events between D. melanogaster and the out-group; conserved TWOB, sites associated with conserved two-way orthologous binding; diverged TWOB, sites associated with diverged two-way orthologous binding; FWOB, sites associated with four-way orthologous binding; Young FWOB, sites associated with those FWOB with inferred evolutionary age <2.5 Myr; Old FWOB, sites associated with those FWOB with inferred evolutionary age >6 Myr. (PDF) [file pbio.1001420.s009.pdf]

Figure S10

**A**

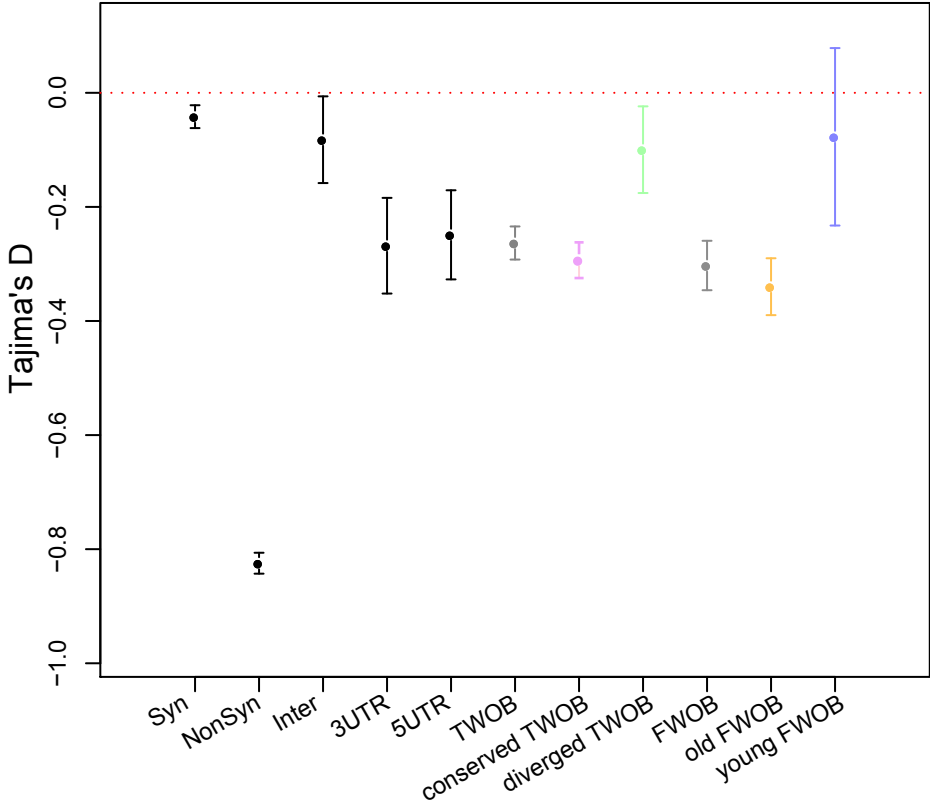

**B**

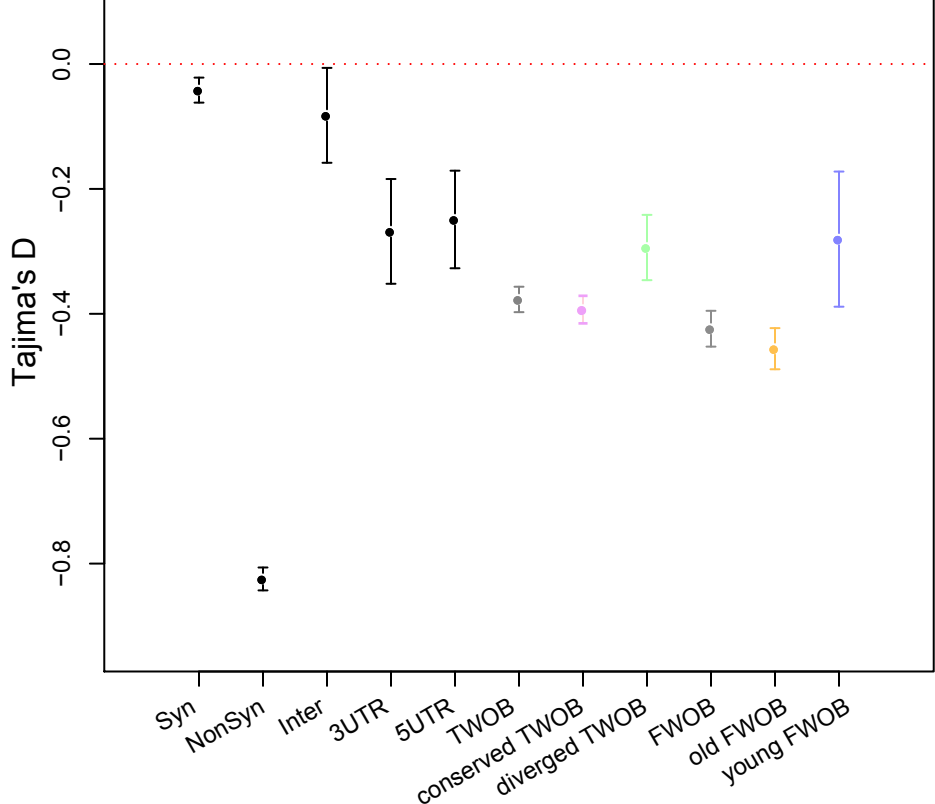

Supplement: Figure S10 — Mean Tajima's D for CTCF-motif and CTCF-201 high-sequence coverage sites. Mean Tajima's D values for different groups of (A) CTCF-motif and (B) CTCF-201 sites after filtering out sites with input sequence coverage <0.5. The center of each circle depicts the mean value, with error bars indicating 2 standard deviations. The out-group species used here is D. simulans. Label abbreviations are the same as for Figure S9. (PDF) [file pbio.1001420.s010.pdf]

Figure S11

A

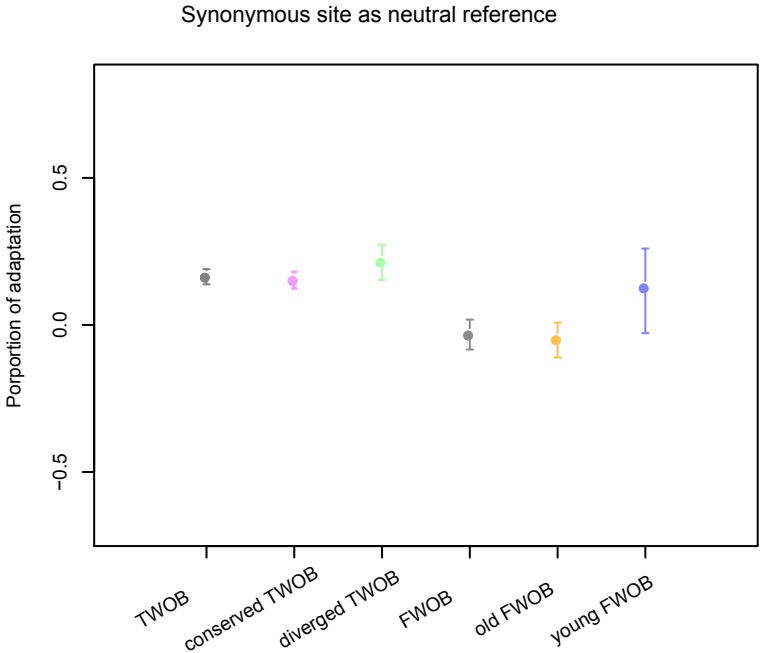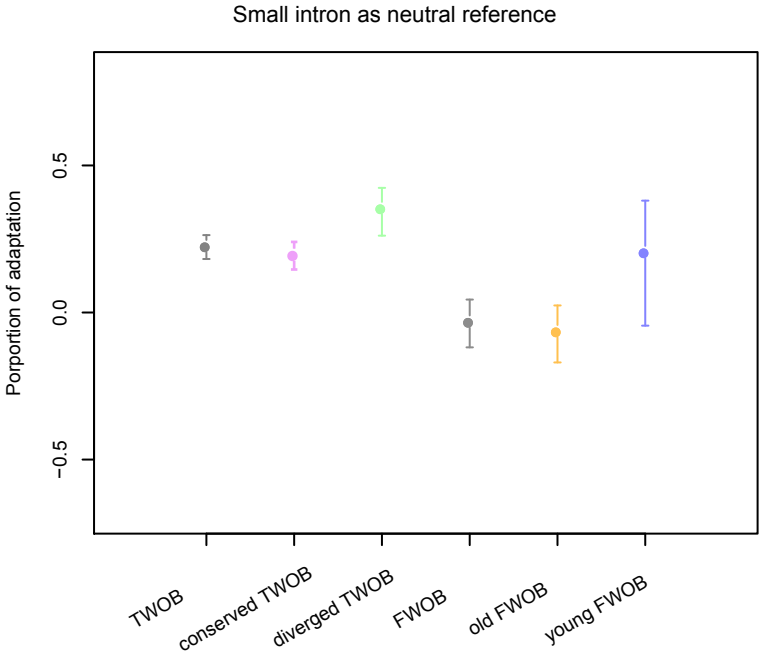

B

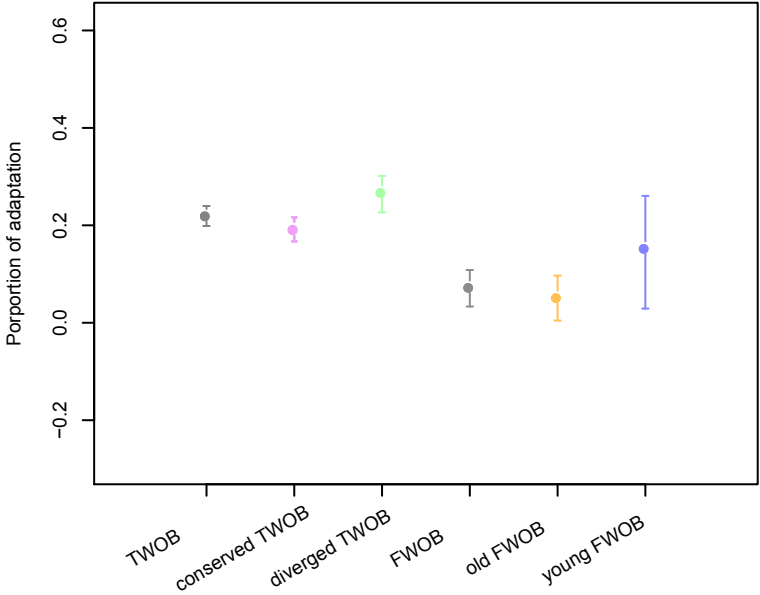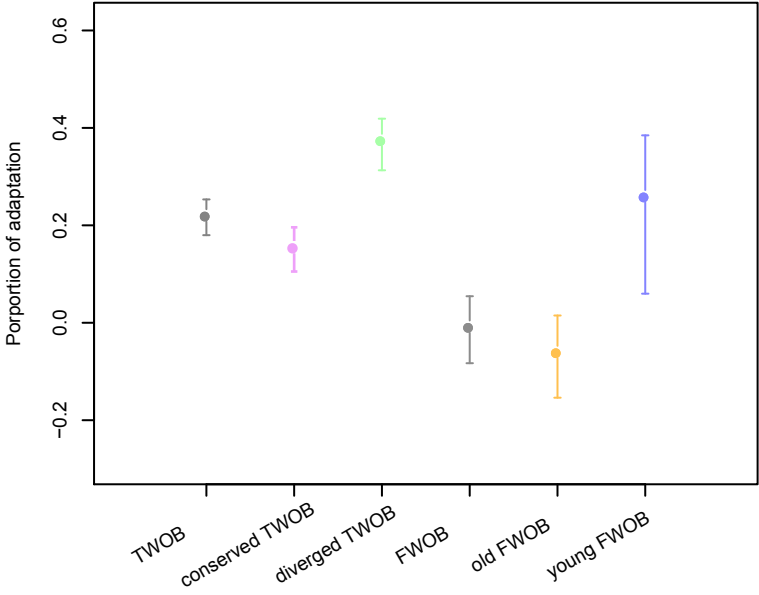

Supplement: Figure S11 — Shared proportion of adaptation in CTCF-201 bp sites. Shared α values estimated for various groups of CTCF-201 bp sites through the extended MK test framework, with (A) D. simulans and (B) D. yakuba as out-group species. The center of each circle in the plot depicts the α value estimated, with error bars indicating the 95% confidence interval. The label abbreviations are the same as for Figure S10. (PDF) [file pbio.1001420.s011.pdf]

Figure S12

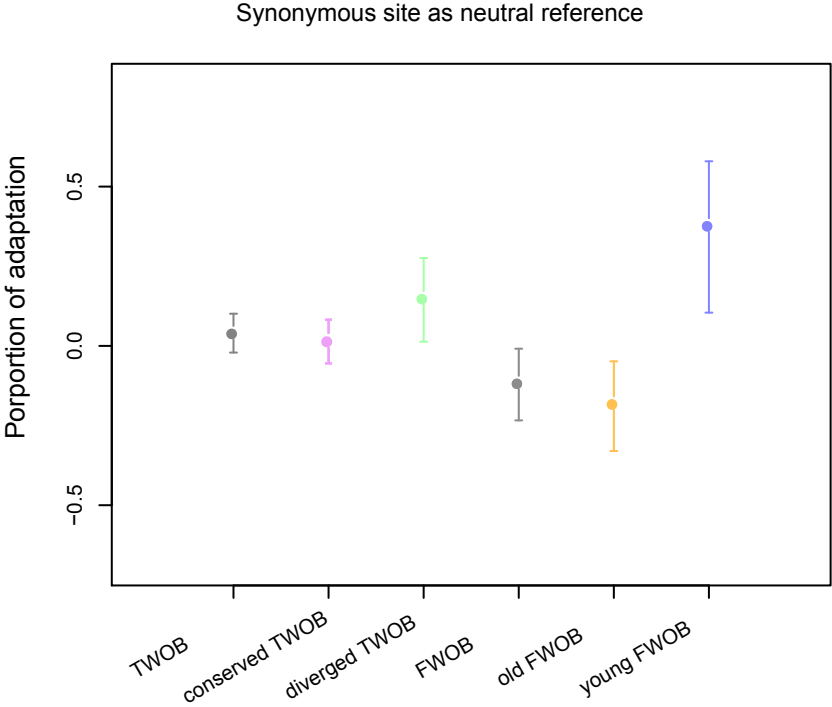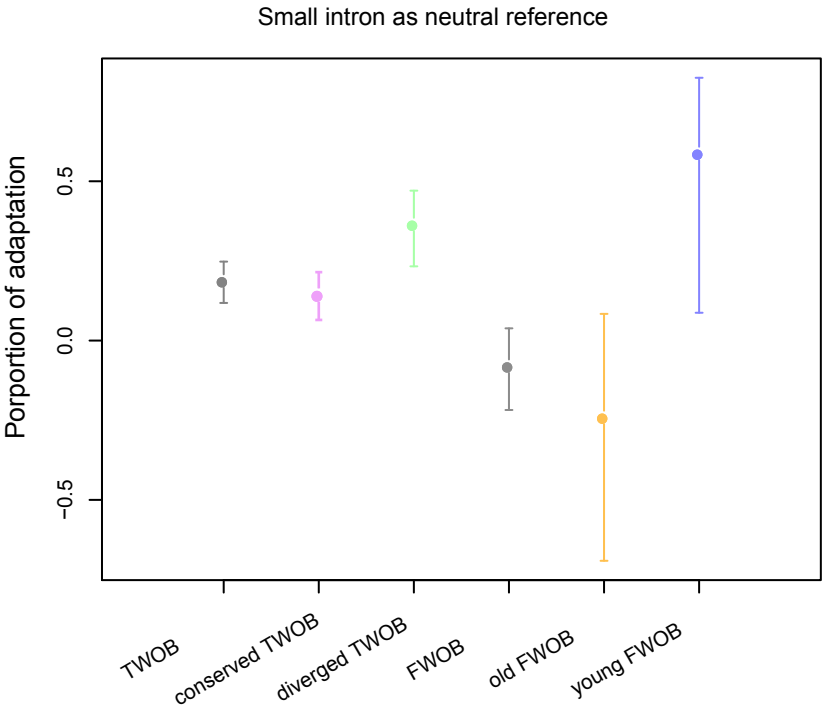

Supplement: Figure S12 — Shared proportion of adaptation in CTCF-motif sites. Shared α values estimated for various groups of CTCF-motif sites through the extended MK test framework with D. simulans as an out-group species. The center of each circle in the plot depicts the α value estimated, with error bars indicating the 95% confidence interval. The label abbreviations are the same as for Figure 3. (PDF) [file pbio.1001420.s012.pdf]

Figure S13

**A**

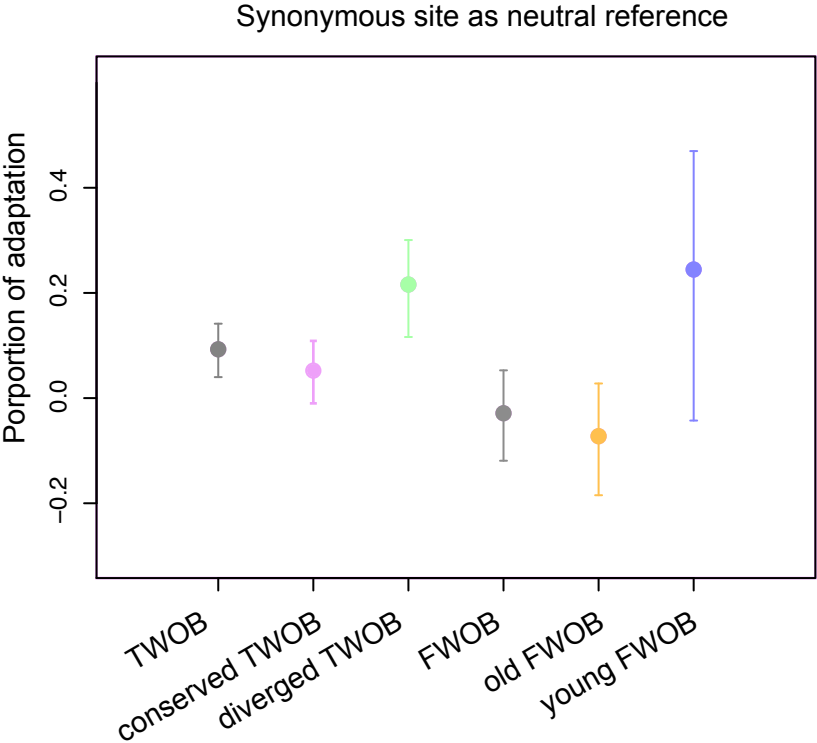

**B**

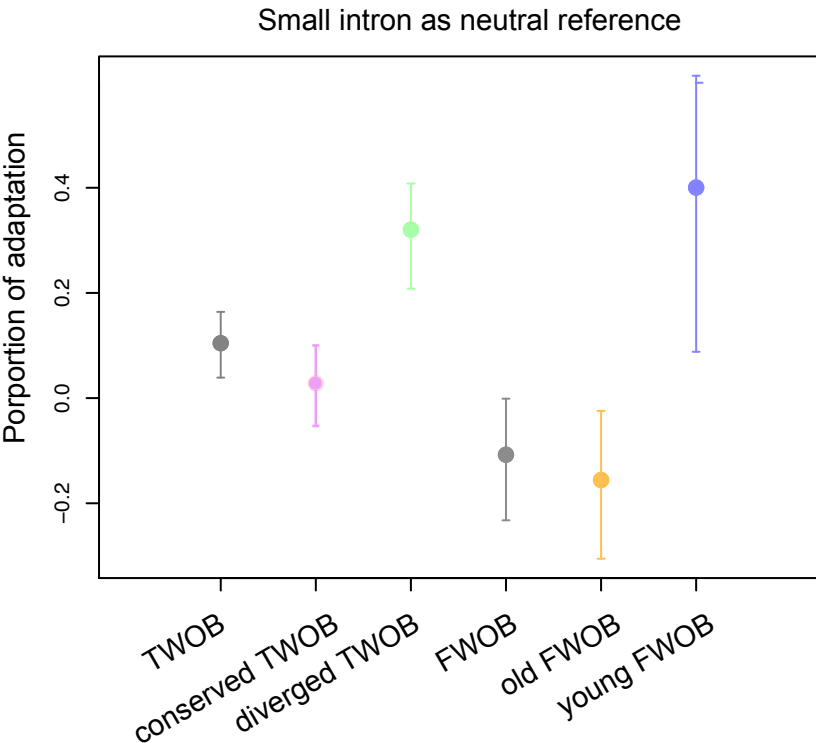

Supplement: Figure S13 — Shared proportion of adaptation in CTCF-motifs high-sequence coverage sites. Shared α values estimated for various groups of CTCF-motif sites after filtering out sites with input sequence coverage <0.5 through the extended MK test framework, with D. yakuba as the out-group species. The center of each circle in the plot depicts the α value estimated, with error bars indicating the 95% confidence interval. The label abbreviations are the same as for Figure 3. (PDF) [file pbio.1001420.s013.pdf]

Figure S14

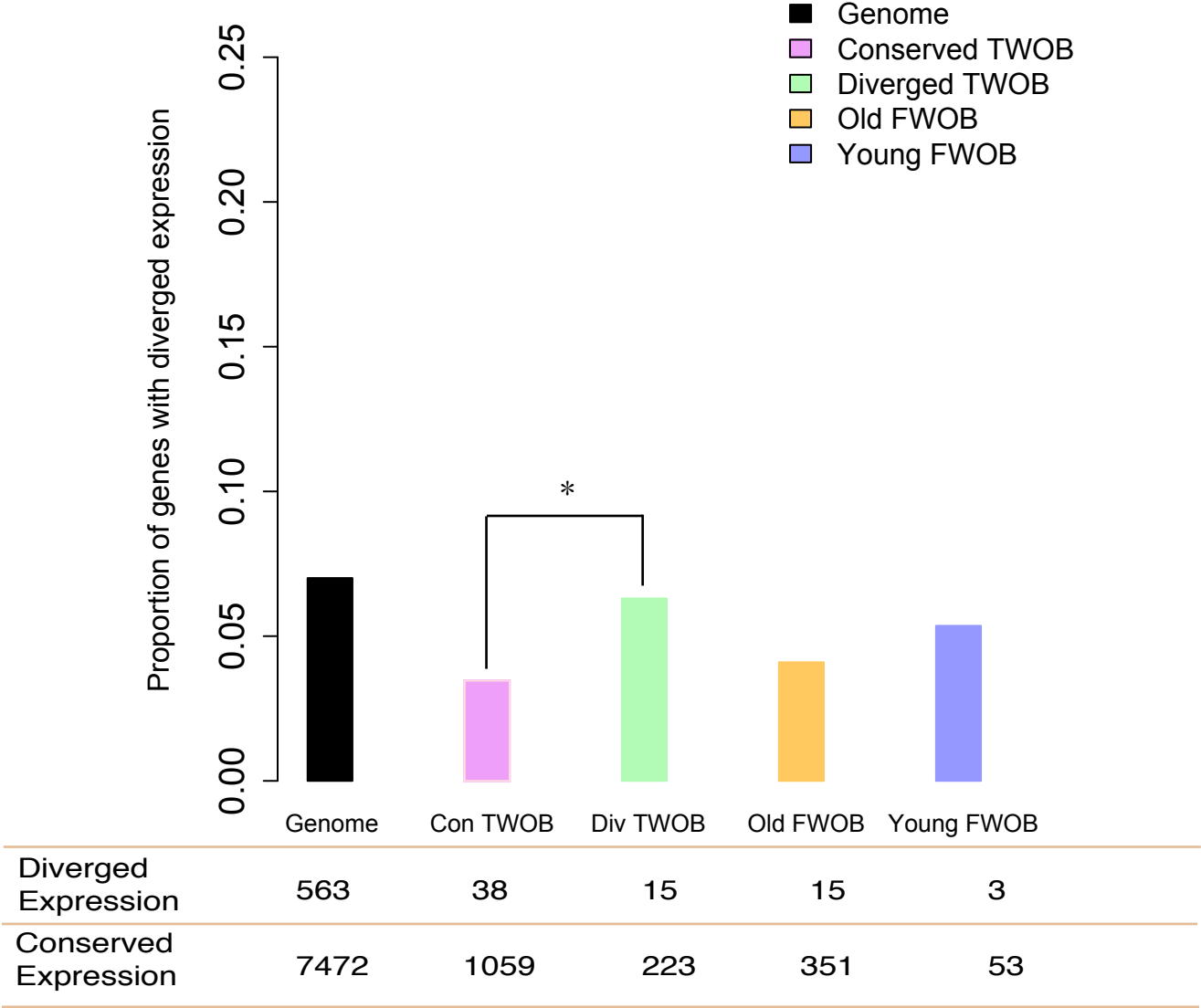

Supplement: Figure S14 — CTCF binding evolution associated with gene expression evolution inferred from microarray data. The bar plots show the proportion of genes with diverged expression between D. melanogaster/D. simulans associated with different groups of CTCF binding sites: Genome-wide (black), Conserved TWOB (pink), Diverged TWOB (green), Old FWOB (orange), and Young FWOB (light purple). The table below each bar plot shows the number of genes with diverged and conserved gene expression in the corresponding comparisons and associated with the corresponding CTCF binding sites. For each groups of CTCF binding sites, the associated genes are the union of the nearest gene to each binding site. The evolutionary status of gene expression (conserved or diverged) is determined using quadruplicate expression profiling with custom-designed species-specific Agilent 105K microarrays. The label abbreviations are the same as for Figure 3. Significance levels: * p<0.05; **p<0.01, one-sided Fisher's exact test. (PDF) [file pbio.1001420.s014.pdf]

Figure S15

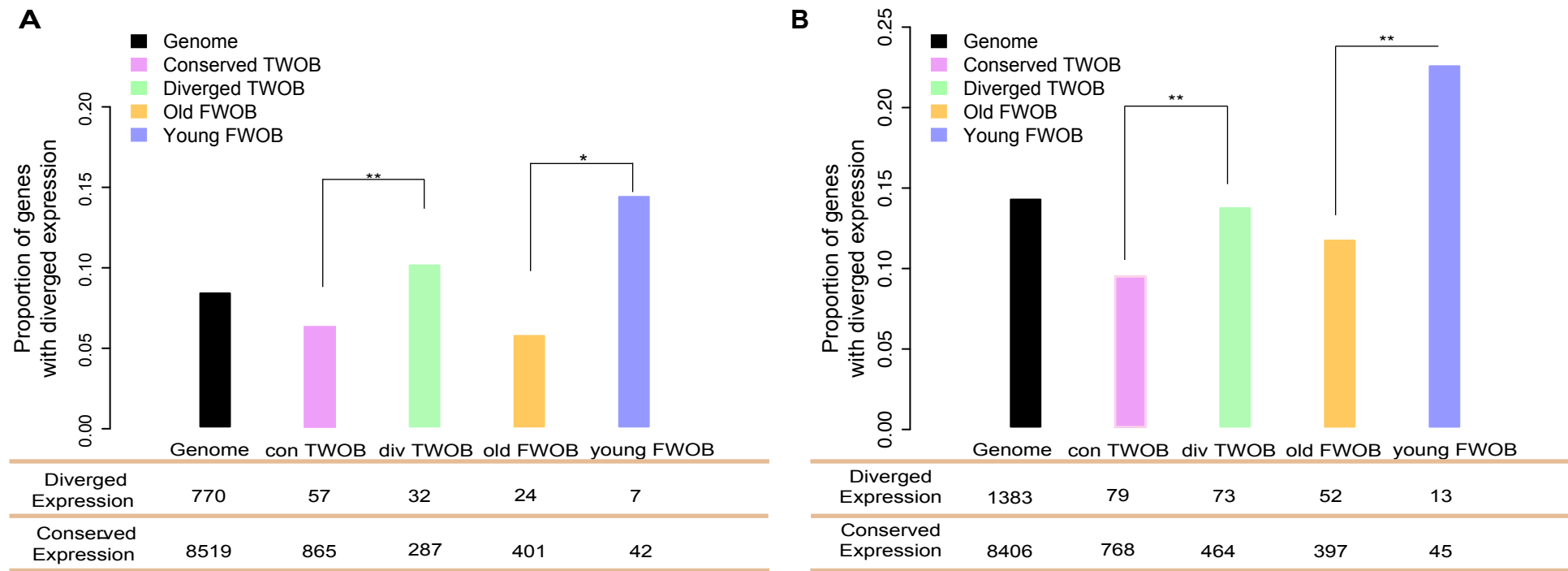

Supplement: Figure S15 — CTCF binding evolution associated with gene expression evolution inferred from RNA-seq data at high-sequence coverage sites. The bar plots show the proportion of genes with diverged expression between (A) D. melanogaster/D. simulans and (B) D. melanogaster/D. yakuba comparisons associated with different groups of CTCF binding sites after filtering out sites with input coverage <0.5. All labels are the same as in Figure 4A and 4B. (PDF) [file pbio.1001420.s015.pdf]

Figure S16

A

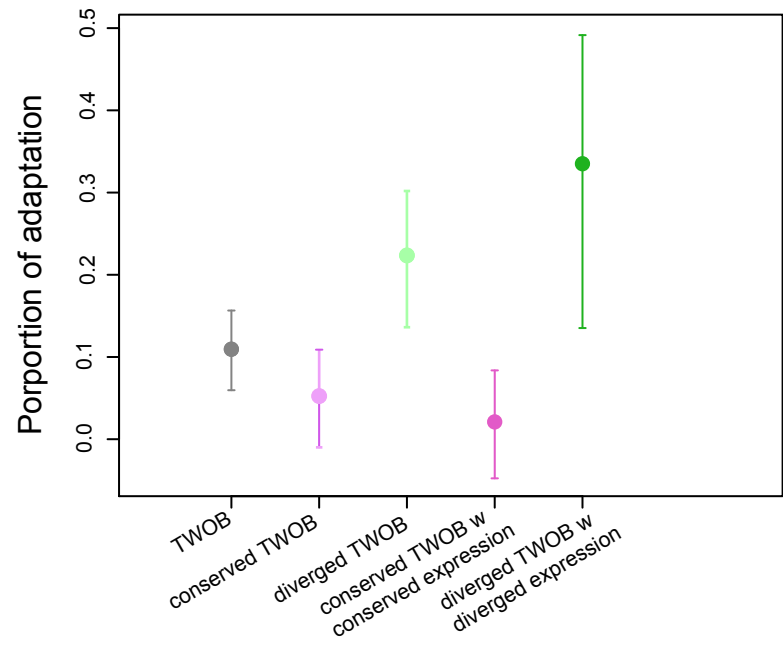

B

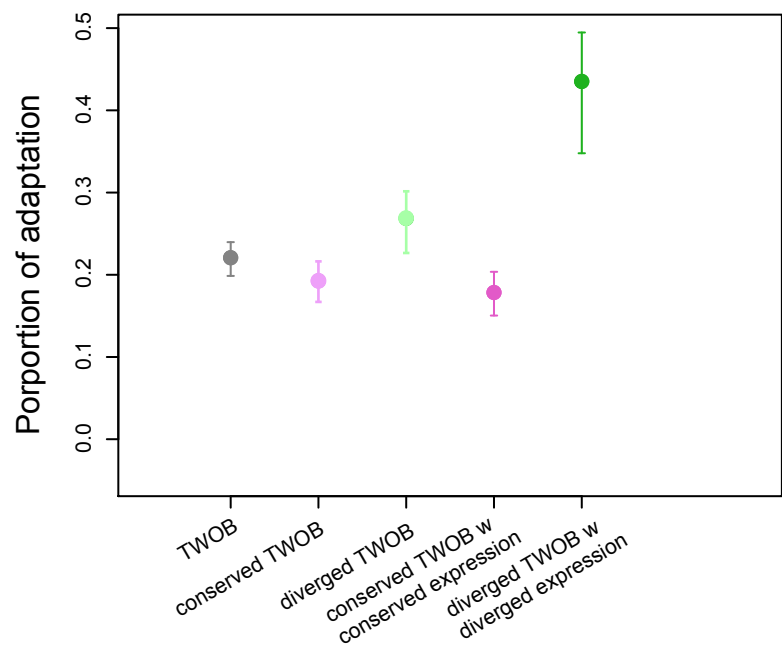

Supplement: Figure S16 — Shared proportion of adaptation in different groups of CTCF TWOB sites. Shared α values estimated for various groups of (A) CTCF-motif sites and (B) CTCF-201 sites through the extended MK test framework using D. yakuba as an out-group. The center of each circle in the plot depicts the α value estimated, with error bars indicating the 95% confidence interval. The label abbreviations: TWOB, Two-Way Orthologous Binding sites identified between D. melanogaster and the outgroup species; diverged TWOB, diverged Two-Way Orthologous Binding sites; conserved TWOB, conserved Two-Way Orthologous Binding sites; conserved TWOB with conserved expression, the subset of conserved Two-Way Orthologous Binding sites for which the expression level of their nearest gene are evolutionarily conserved; diverged TWOB with diverged expression, the subset of diverged Two-Way Orthologous Binding sites for which the expression level of their nearest gene are evolutionarily diverged. (PDF) [file pbio.1001420.s016.pdf]

Figure S17

A

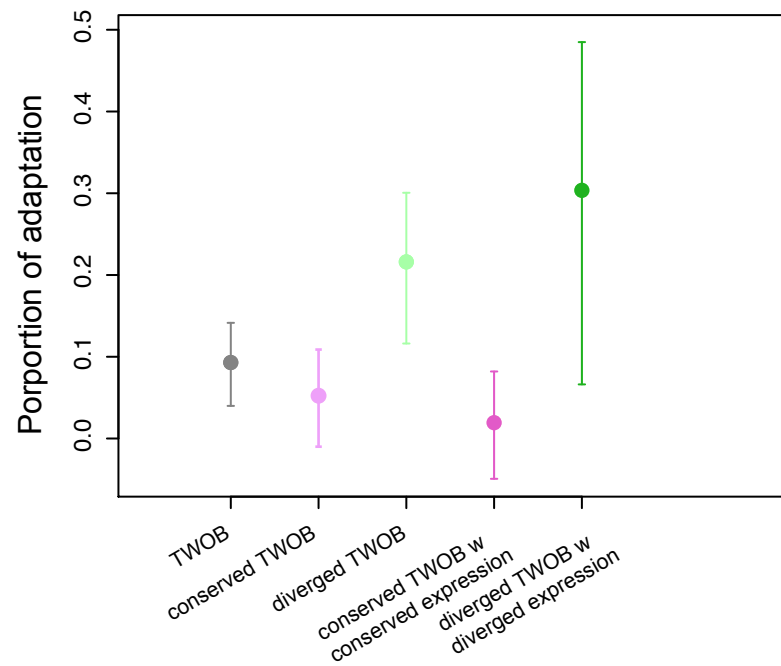

B

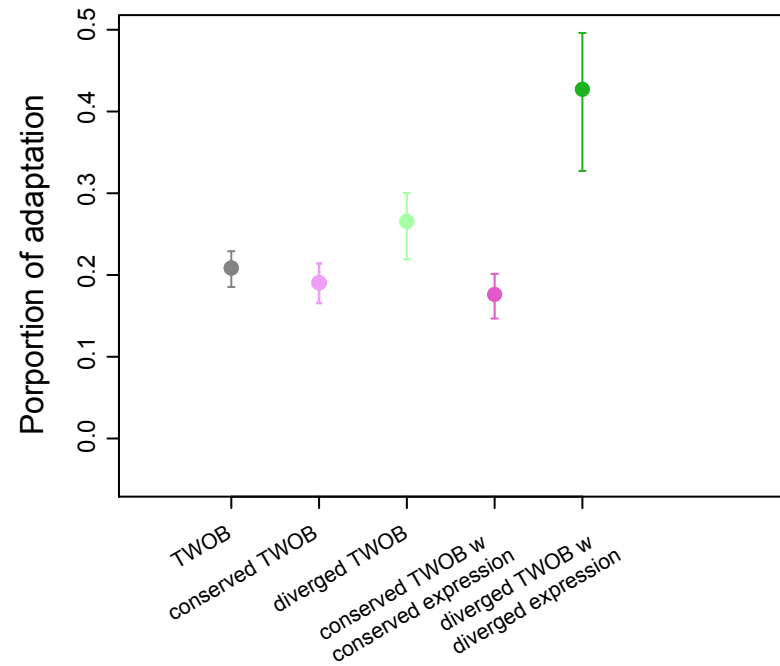

Supplement: Figure S17 — Shared proportion of adaptation in different groups of CTCF TWOB high-sequence coverage sites. Shared α values estimated for various groups of (A) CTCF-motif sites and (B) CTCF-201 sites through the extended MK test framework using D. yakuba as an out-group. The sites used here for α estimation are those sites with input sequence coverage >0.5. All labels and abbreviations are the same as in Figure S16. (PDF) [file pbio.1001420.s017.pdf]

Figure S18

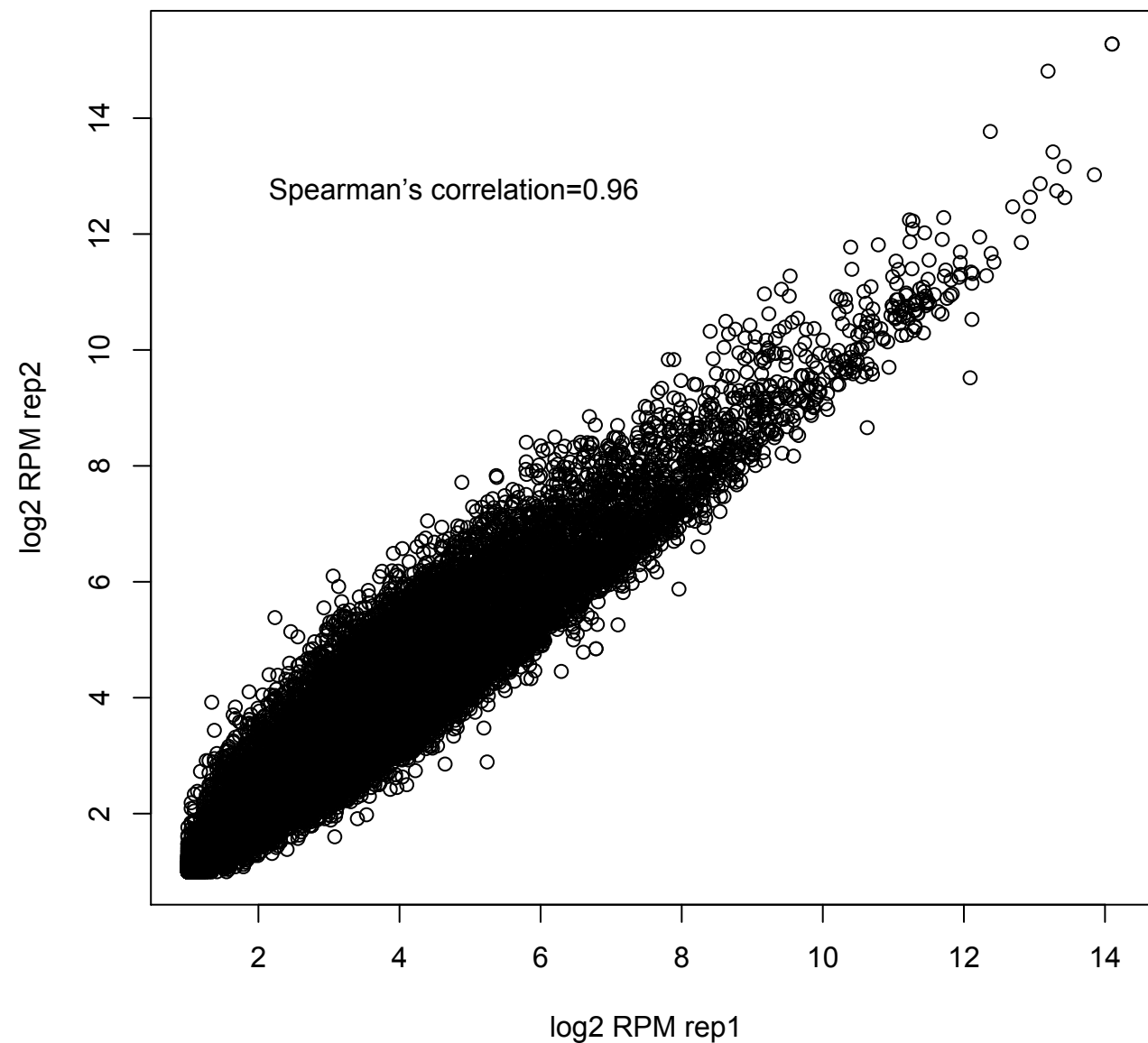

Supplement: Figure S18 — Reproducibility of RNA-seq data. The scatter plot shows the high correlation between gene RPM (number of reads per million) values from two D. melanogaster WPP biological samples. The estimated Spearman's rank order correlation is 0.96. (PDF) [file pbio.1001420.s018.pdf]

Figure S19

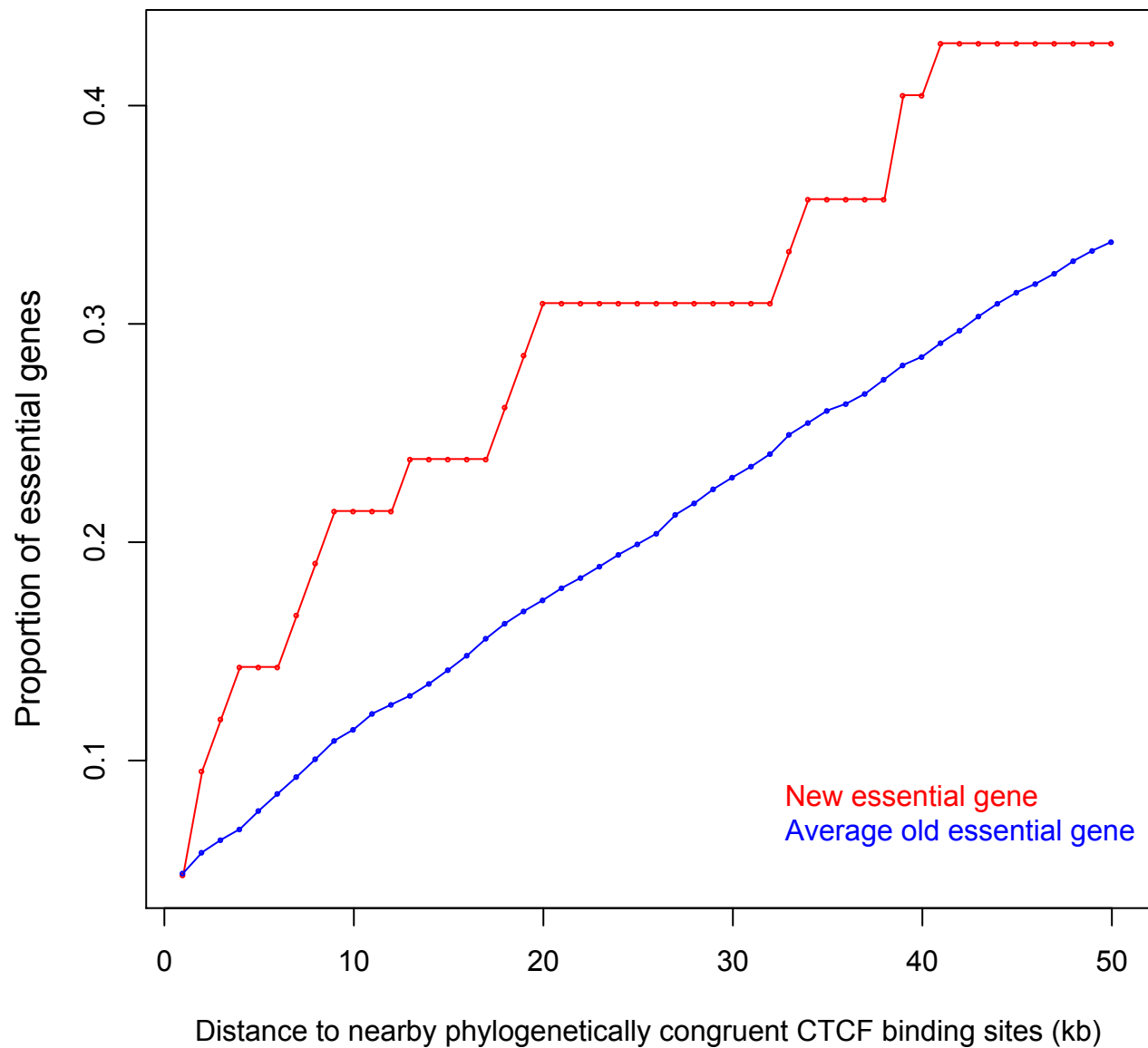

Supplement: Figure S19 — Association between essential genes and CTCF binding events. The red dotted line shows the cumulative proportions of 42 new essential genes (originated less than 25 Myr ago) with phylogenetically congruent CTCF binding sites within flanking regions of various lengths. The blue dotted line shows the cumulative average proportions of randomly sampled 42 old essential genes (originated more than 40 Myr ago) with phylogenetically congruent CTCF binding sites obtained from 1,000 simulations. A CTCF binding site is described as phylogenetically congruent to a gene if and only if the binding event appears in the exactly same branches as the gene on the evolutionary tree. The difference between the two cumulative lines is significant, p<1e-6, Komogorov Smirnov test. (PDF) [file pbio.1001420.s019.pdf]

Figure S20

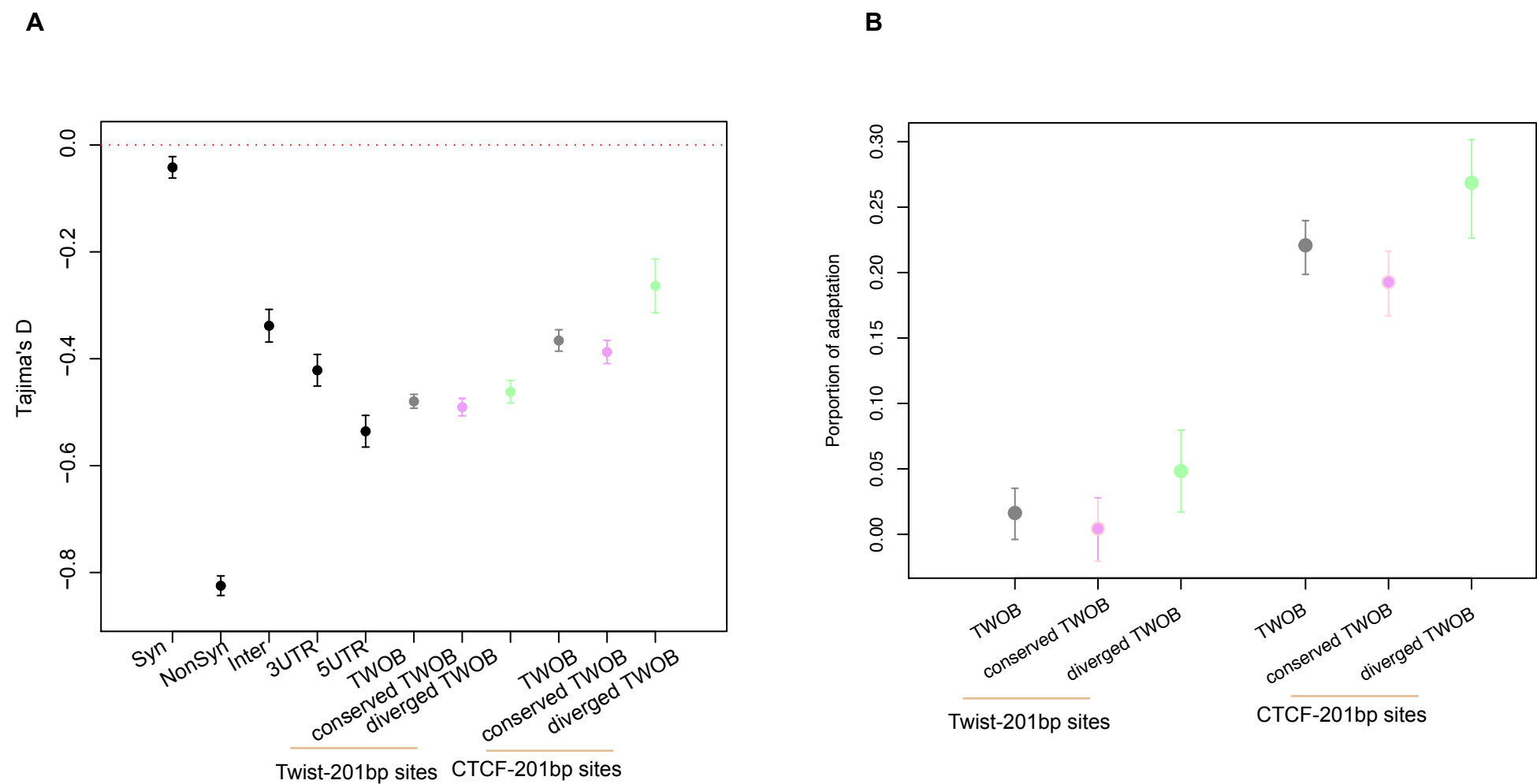

Supplement: Figure S20 — Selection signatures in Twist-201 bp sites. (A) Mean Tajima's D values for Twist-201 bp sites. The center of each circle depicts the mean value, with error bars indicating 2 standard deviations. The out-group species used here is D. yakuba. (B) Shared proportion of adaptation (alpha) estimated for Twist-201 bp sites using D. yakuba as the out-group. The center of each circle in the plot depicts the α value estimated, with error bars indicating the 95% confidence interval. The mean Tajima's D values as well as alpha values for Twist-201 bp sites are plotted together with CTCF-201 bp sites (as labeled in the figure). TWOB, diverged TWOB, and conserved TWOB for Twist and for CTCF are defined the same way as in Figure 3. The TWOB, diverged TWOB, and conserved TWOB Twist binding sites were identified by applying our analysis method to the Twist comparative data. (PDF) [file pbio.1001420.s020.pdf]
