# Supplementary material for: Adaptive Evolution and the Birth of CTCF Binding Sites in the Drosophila Genome
Source: PLoS Biol. 2012 Nov 6;10(11):e1001420. doi: 10.1371/journal.pbio.1001420 (PMC3491045; doi:10.1371/journal.pbio.1001420)
Supplement: Figure S4 — Spearman's rank correlation between ChIP-seq replicates. The Spearman's correlation coefficients (rank order correlation) were calculated with CDP scores (compiled density profile, a QuEST transformation of the sequence depth data for peak calling) in the 500 bp flanking region around the peak summit coordinate for each individual binding peak between any two replicates. The box plots show the overall distribution of Spearman's correlation coefficients for summarized overall combinations of replicate pairs identified in (A) D. melanogaster, (B) D. simulans, (C) D. yakuba, and (D) D. pseudoobscura. (PDF) [file pbio.1001420.s004.pdf]

Figure S4

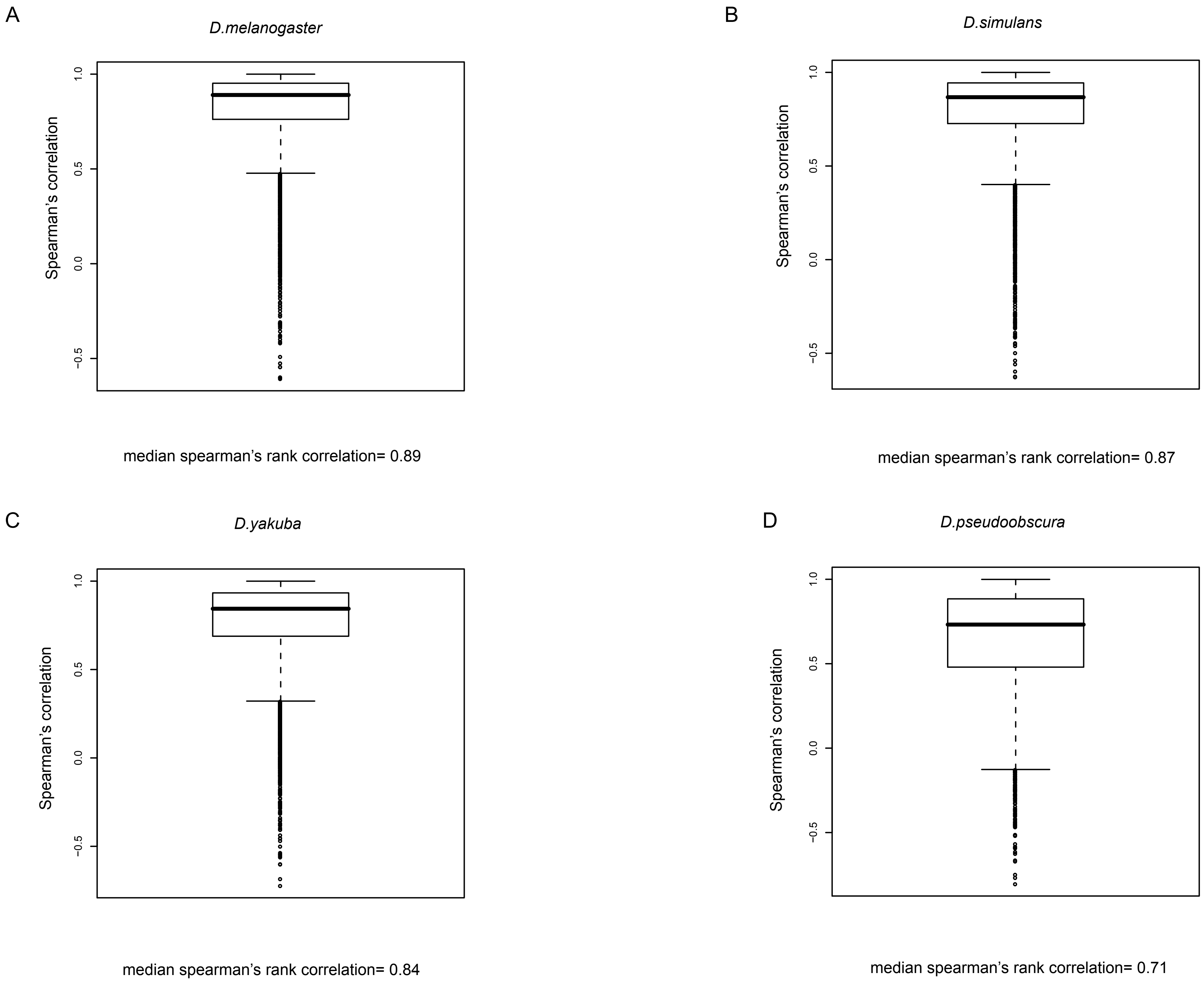
A
D.melanogaster
Spearman's correlation
1.0
0.5
0.0
-0.5
median spearman's rank correlation= 0.89
B
D.simulans
Spearman's correlation
1.0
0.5
0.0
-0.5
median spearman's rank correlation= 0.87
C
D.yakuba
Spearman's correlation
1.0
0.5
0.0
-0.5
median spearman's rank correlation= 0.84
D
D.pseudoobscura
Spearman's correlation
1.0
0.5
0.0
-0.5
median spearman's rank correlation= 0.71
