# Supplementary material for: Adaptive Evolution and the Birth of CTCF Binding Sites in the Drosophila Genome
Source: PLoS Biol. 2012 Nov 6;10(11):e1001420. doi: 10.1371/journal.pbio.1001420 (PMC3491045; doi:10.1371/journal.pbio.1001420)
Supplement: Figure S5 — Sequence alignments of CTCF protein DNA binding domain (DBD) in the four species. The 11 yellow colored blocks represent the 11 predicted C2H2 zinc finger domains using online domain finding software provided by the Pfam database. Different color depicts different types of amino acids compared to the consensus ones: amino acids that are identical to the consensus (in black); amino acids that are different but with similar properties to the consensus (in blue); and amino acids that are different and have different properties from the consensus (in red). (PDF) [file pbio.1001420.s005.pdf]

Figure S5

D.mel CTCF DBD	YSCPHCPYTASKKFLITRHSRSHDVEPSFKCSICERSFRSNVGLQNHINTHMGNKPHKCKLCESAFTTSGELVRHTRYKHTKEKPHKCTECTYASVELTK
D.sim CTCF DBD	YSCPHCPYTASKKFLITRHSRSHDVEPSFKCSICERSFRSNVGLQNHINTHMGNKPHKCKLCESAFTTSGELVRHTRYKHTKEKPHKCTECTYASVELTK
D.yak CTCF DBD	YSCPHCPYTANKKFLITRHSRSHDVEPSFKCSICERSFRSNVGLQNHVNTHMGKKPHKCKLCESAFTTSGELVRHTRYKHTKEKPHKCTECTYASVELTK
D.pse CTCF DBD	FNCSQCAYTTNKKFLITRHNKTHETDFSYKCSICDRGFKSNVGLVNHVNTHLGNKPHKCKHCESAFVTSGELIRHTRYKHTKEKPHKCTECSYASVELTK

D.mel CTCF DBD	LRRHMTCHTGERPYQCPHCTYASQDMFKLKRHMVIHTGEKKYQCDICKSRFTQSNSLKAHKLIHSVVDKPVFQCNYCPTTCGRKADLRVHIKHMHTSDVP
D.sim CTCF DBD	LRRHMTCHTGERPYQCPHCTYASQDMFKLKRHMVIHTGEKKYQCDICKSRFTQSNSLKAHKLIHSVVDKPVFQCNYCPTTCGRKADLRVHIKHMHTSDVP
D.yak CTCF DBD	LRRHMTCHTGERPYQCPHCTYASQDMFKLKRHMVIHTGEKKYQCDICKSRFTQSNSLKAHKLIHSVVDKPVFQCNFCPTTCGRKADLRIHIKHMHTSDVP
D.pse CTCF DBD	LRRHMTCHTGERPYQCPHCTYASQDMFKLKRHLVVHTGEKRYQCDICKSRFTQSNSLKAHKLIHSVVDKPVFQCSHCPTTCGRKADLRLHVMHMHTADKP

D.mel CTCF DBD	MTCRRCGQQLPDRYQYKLHVKSHEGEKCYSCKLCSYASVTQRHLASHMLIHLDEKPFHCDQCPQAFRQRQLLRRHMNLVHNEEYQPPEPREKLHKCPSCP
D.sim CTCF DBD	ITCRRCGQQLPDRYQYKLHVKSHEGEKCYSCKLCSYASVTQRHLASHMLIHLDEKPFHCDQCPQAFRQRQLLRRHMNLVHNEEYQPPEPREKLHKCPSCP
D.yak CTCF DBD	ITCRRCGQQLPDRYQYKLHVKSHEGEKCYSCKLCSYASVTQRHLASHMLIHLDEKPFHCEQCPQAFRQRQLLRRHMNLVHNEEYQPPAPRAKLHKCPSCP
D.pse CTCF DBD	IPCKRCGQNLPDRYQYKLHIKTHEGERCYRCKLCEYASVSQRHLDSHMLVHLDAKPFKCEVCPQAFRQRQLLRRHMNLVHNEEYTRPEPREKMHSCPECP

D.mel CTCF DBD	REFTHKGNLMRHMETH
D.sim CTCF DBD	REFTHKGNLMRHMETH
D.yak CTCF DBD	REFTHKGNLMRHMEIH
D.pse CTCF DBD	RIFTHKGNLIRHMEIH
