# Supplementary material for: Adaptive Evolution and the Birth of CTCF Binding Sites in the Drosophila Genome
Source: PLoS Biol. 2012 Nov 6;10(11):e1001420. doi: 10.1371/journal.pbio.1001420 (PMC3491045; doi:10.1371/journal.pbio.1001420)
Supplement: Table S1 — Summary of sequence reads. (A) Number of Solexa sequence reads for ChIP-seq experiments. (B) Number of LiftOver reads of non–D. melanogaster species. (C) Number of Solexa sequencing reads for RNA-seq experiments. (PDF) [file pbio.1001420.s021.pdf]

Supporting Tables

Table S1: Summary of Sequence Reads

Table S1A: Number of Solexa sequence reads for ChIP-seq experiments

Species	Sample	No. of unique reads (in million)	Genome Coverage
D.melanogaster	CTCF1	5.59	1.68
D.melanogaster	CTCF2	5.74	1.72
D.melanogaster	CTCF3	4.44	1.33
D.melanogaster	Input1	7.24	2.17
D.melanogaster	Input2	6.57	1.97
D.melanogaster	Input3	7.3	2.19
D.simulans	CTCF1	5.13	1.68
D.simulans	CTCF2	2.96	0.97
D.simulans	CTCF3	6.82	2.24
D.simulans	Input1	6.26	2.05
D.simulans	Input2	6.26	2.05
D.simulans	Input3	9.41	3.09
D.yakuba	CTCF1	4.38	1.32
D.yakuba	CTCF2	6.32	1.90
D.yakuba	CTCF3	5.75	1.73
D.yakuba	Input1	7.55	2.27
D.yakuba	Input2	7.55	2.27
D.yakuba	Input3	7.55	2.27
D.pseudoobscura	CTCF1	6.11	1.73
D.pseudoobscura	CTCF2	7.59	2.15
D.pseudoobscura	CTCF3	7.48	2.12
D.pseudoobscura	Input1	7.06	2.00
D.pseudoobscura	Input2	7.06	2.00
D.pseudoobscura	Input3	8.37	2.37

Note: Column 3 "No. of unique reads" summarizes the number of sequence reads that are uniquely mapped to certain genome position allowing at most two mismatches. Column 4 "Genome Coverage" are calculated as the read length (36) multiplies the number of unique reads divided by the number of total nucleotides in each sequenced genome .

Table S1B: Number of LiftOver reads of non-*D.melanogaster* species

Species	**Sample**	**No. of LiftOver reads (in million)**
D.simulans	CTCF1	5.02
D.simulans	CTCF2	2.89
D.simulans	CTCF3	6.71
D.simulans	input1	6.1
D.simulans	input2	6.1
D.simulans	input3	9.26
D.yakuba	CTCF1	4.1
D.yakuba	CTCF2	5.94
D.yakuba	CTCF3	5.3
D.yakuba	input1	7.03
D.yakuba	input2	7.03
D.yakuba	input3	7.03
D.pseudoobscura	CTCF1	4.75
D.pseudoobscura	CTCF2	5.62
D.pseudoobscura	CTCF3	5.11
D.pseudoobscura	input1	5.68
D.pseudoobscura	input2	5.68
D.pseudoobscura	input3	6.81

Table S1C: Number of Solexa Sequencing reads for RNA-seq experiments

**Species**	**Sample**	**Number of unique reads mapped to Flybase annotated gene coding regions (in million)**
D.melanogaster	WPP1	15.53
D.melanogaster	WPP2	16.77
D.melanogaster	WPP3	12.58
D.simulans	WPP1	15.98
D.simulans	WPP2	11.75
D.simulans	WPP3	9.25
D.yakuba	WPP1	14.80
D.yakuba	WPP2	13.37
D.yakuba	WPP3	12.60
