# Supplementary material for: Adaptive Evolution and the Birth of CTCF Binding Sites in the Drosophila Genome
Source: PLoS Biol. 2012 Nov 6;10(11):e1001420. doi: 10.1371/journal.pbio.1001420 (PMC3491045; doi:10.1371/journal.pbio.1001420)
Supplement: Table S2 — Ka/Ks ratio for CTCF gene in Drosophila species. (PDF) [file pbio.1001420.s022.pdf]

Table S2: Ka/Ks ratio for CTCF gene in *Drosophila* species

D.melanogaster		0.123	0.106	0.137
D.simulans	0.013		0.133	0.151
D.yakuba	0.044	0.058		0.147
D.pseudoobscura	0.072	0.080	0.094	

Upper right: Ka/Ks ratio for the whole gene (in green).
Lower left: Ka/Ks ratio for the predicted DNA binding domain (in pink).
