# Supplementary material for: Adaptive Evolution and the Birth of CTCF Binding Sites in the Drosophila Genome
Source: PLoS Biol. 2012 Nov 6;10(11):e1001420. doi: 10.1371/journal.pbio.1001420 (PMC3491045; doi:10.1371/journal.pbio.1001420)
Supplement: Table S3 — CTCF binding site motif enrichment in each species. (PDF) [file pbio.1001420.s023.pdf]

Table S3: CTCF Binding site motif enrichment in each species

Species	Motif searching Pvalue cutoff	ChIP enriched sites	Simulated sites		Fold enrichment (X)	Z test	
			mean	s.d.		Z statistic	pvalue
D.mel	1.00E-02	98.90%	96.10%	0.0059	1.03	15.138	<0.0001
	5.00E-03	92.00%	81.70%	0.0073	1.126	44.712	<0.0001
	1.00E-03	65.00%	31.50%	0.0169	2.063	62.729	<0.0001
	5.00E-04	56.20%	19.00%	0.0114	2.951	103.021	<0.0001
	1.00E-04	42.70%	4.30%	0.006	9.873	200.774	<0.0001
D.sim	1.00E-02	98.70%	87.50%	0.0052	1.128	68.84	<0.0001
	5.00E-03	93.60%	74.30%	0.0124	1.261	49.525	<0.0001
	1.00E-03	66.30%	30.70%	0.0085	2.158	132.689	<0.0001
	5.00E-04	56.60%	19.10%	0.0084	2.971	142.256	<0.0001
	1.00E-04	40.40%	3.90%	0.0049	10.363	237.959	<0.0001
D.yak	1.00E-02	98.70%	93.00%	0.0045	1.061	40.213	<0.0001
	5.00E-03	92.60%	77.90%	0.0097	1.19	48.31	<0.0001
	1.00E-03	60.60%	29.30%	0.0053	2.07	187.813	<0.0001
	5.00E-04	49.00%	16.20%	0.0052	3.02	197.49	<0.0001
	1.00E-04	34.30%	3.90%	0.0018	8.74	524.615	<0.0001
D.pse	1.00E-02	98.70%	91.90%	0.0046	1.074	46.43	<0.0001
	5.00E-03	92.70%	80.40%	0.0071	1.153	54.352	<0.0001
	1.00E-03	61.10%	35.40%	0.0051	1.728	159.301	<0.0001
	5.00E-04	48.90%	21.20%	0.0085	2.311	103.348	<0.0001
	1.00E-04	32.20%	5.30%	0.0041	6.066	205.104	<0.0001

Note: Column 3 shows the percentages of identified CTCF binding sites in each species containing at least one species-specific motif at various P value cutoffs (shown in Column 2). Column 4 summerizse the mean and standard deviation of the percentages

of randomly simulated sites in each species containing at least one species-specific motif at various P value cutoffs based on 10 simulations. For each simulation, we randomly generated the same number of 201bp sites on each chromosome as identified for the CTCF binding sites in each species, and applied exactly same procedure and parameters for motif searching in these sequences. Column 5 shows the fold enrichment of species-specific motif in ChIP identified sites over the randomly simulated sites.
