# Supplementary material for: Adaptive Evolution and the Birth of CTCF Binding Sites in the Drosophila Genome
Source: PLoS Biol. 2012 Nov 6;10(11):e1001420. doi: 10.1371/journal.pbio.1001420 (PMC3491045; doi:10.1371/journal.pbio.1001420)
Supplement: Table S4 — Pearson's correlation coefficients between ChIP replicates. (PDF) [file pbio.1001420.s024.pdf]

Table S4: Pearson's correlation coefficients between ChIP replicates

Species	rep 1 vs rep2	rep1 vs rep 3	rep 2 vs rep 3	mean
D.melanogaster	0.90	0.95	0.89	0.91
D.simulans	0.98	0.83	0.84	0.88
D.yakuba	0.96	0.86	0.92	0.91
D.pseudoobscura	0.86	0.72	0.85	0.81

Note: The genome wide pearson's correlation coefficients between replicates were calculated using raw sequence read counts generated from each ChIP sequence sample, with no additional data normalization.
