# Supplementary material for: Adaptive Evolution and the Birth of CTCF Binding Sites in the Drosophila Genome
Source: PLoS Biol. 2012 Nov 6;10(11):e1001420. doi: 10.1371/journal.pbio.1001420 (PMC3491045; doi:10.1371/journal.pbio.1001420)
Supplement: Table S5 — Diverged and conserved CTCF binding events. (PDF) [file pbio.1001420.s025.pdf]

Table S5: Diverged and conserved CTCF binding events

Parameter : 0.4

Species A	Species B	All Binding Events						
		Total	Species A binding events				Species B specific	Binding divergence
			All	Species A specific	Shared with Species B	Species A centric binding divergence		
D.mel	*D.sim*	2430	2267	446	1821	19.67%	163	25.06%
D.mel	*D.yak*	2514	2267	660	1607	29.11%	247	36.08%
D.mel	*D.pse*	2382	2267	1679	588	74.06%	115	75.31%

Species A	Species B	Two Way Orthologous Binding Events						
		Total	Species A binding events				Species B specific	Binding divergence
			All	Species A specific	Shared with Species B	Species A centric binding divergence		
D.mel	*D.sim*	2326	2168	376	1792	17.34%	158	22.96%
D.mel	*D.yak*	2454	2214	621	1593	28.05%	240	35.09%
D.mel	*D.pse*	1165	1075	735	340	68.37%	90	70.82%

Species A	Species B	Four Way Orthologous Binding Events						
		Total	Species A binding events				Species B specific	Binding divergence
			All	Species A specific	Shared with Species B	Species A centric binding divergence		
D.mel	*D.sim*	1130	1030	157	873	15.24%	100	22.74%
D.mel	*D.yak*	1159	1030	271	759	26.31%	129	34.51%
D.mel	*D.pse*	1116	1030	701	329	68.06%	86	70.52%

Parameter : 0.35

Species A	Species B	All Binding Events						
		Total	Species A binding events				Species B specific	Binding divergence
			All	Species A specific	Shared with Species B	Species A centric binding divergence		
D.mel	*D.sim*	2713	2544	496	2048	19.50%	169	24.51%
D.mel	*D.yak*	2808	2544	742	1802	29.17%	264	35.83%
D.mel	*D.pse*	2683	2544	1817	727	71.42%	139	72.90%

Species A	Species B	Two Way Orthologous Binding Events						
		Total	Species A binding events				Species B specific	Binding divergence
			All	Species A specific	Shared with Species B	Species A centric binding divergence		
D.mel	*D.sim*	2602	2437	420	2017	17.23%	165	22.48%
D.mel	*D.yak*	2745	2490	701	1789	28.15%	255	34.83%
D.mel	*D.pse*	1334	1222	797	425	65.22%	112	68.14%

Species A	Species B	Four Way Orthologous Binding Events						
		Total	Species A binding events				Species B specific	Binding divergence
			All	Species A specific	Shared with Species B	Species A centric binding divergence		
D.mel	*D.sim*	1280	1176	183	993	15.56%	104	22.42%
D.mel	*D.yak*	1314	1176	316	860	26.87%	138	34.55%
D.mel	*D.pse*	1284	1176	763	413	64.88%	108	67.83%

Parameter : 0.5

Species A	Species B	All Binding Events						
		Total	Species A binding events				Species B specific	Binding divergence
			All	Species A specific	Shared with Species B	Species A centric binding divergence		
D.mel	*D.sim*	2046	1913	389	1524	20.33%	133	25.51%
D.mel	*D.yak*	2143	1913	547	1366	28.59%	230	36.26%
D.mel	*D.pse*	2005	1913	1470	443	76.84%	92	77.91%

Species A	Species B	Two Way Orthologous Binding Events						
		Total	Species A binding events				Species B specific	Binding divergence
			All	Species A specific	Shared with Species B	Species A centric binding divergence		
D.mel	*D.sim*	1953	1824	322	1502	17.65%	129	23.09%
D.mel	*D.yak*	2086	1865	510	1355	27.35%	221	35.04%
D.mel	*D.pse*	959	889	644	245	72.44%	70	74.45%

Species A	Species B	Four Way Orthologous Binding Events						
		Total	Species A binding events				Species B specific	Binding divergence
			All	Species A specific	Shared with Species B	Species A centric binding divergence		
D.mel	*D.sim*	927	850	137	713	16.12%	77	23.09%
D.mel	*D.yak*	971	850	212	638	24.94%	121	34.29%
D.mel	*D.pse*	918	850	614	236	72.24%	68	74.29%

Parameter : 1.0

Species A	Species B	All Binding Events						
		Total	Species A binding events				Species B specific	Binding divergence
			All	Species A specific	Shared with Species B	Species A centric binding divergence		
D.mel	*D.sim*	1250	1169	251	918	21.47%	81	26.56%
D.mel	*D.yak*	1297	1169	350	819	29.94%	128	36.85%
D.mel	*D.pse*	1229	1169	947	212	81.01%	60	81.94%

Species A	Species B	Two Way Orthologous Binding Events						
		Total	Species A binding events				Species B specific	Binding divergence
			All	Species A specific	Shared with Species B	Species A centric binding divergence		
D.mel	*D.sim*	1194	1118	208	910	18.60%	76	23.79%
D.mel	*D.yak*	1262	1142	329	813	28.81%	120	35.58%
D.mel	*D.pse*	565	524	406	118	77.48%	41	79.12%

Species A	Species B	Four Way Orthologous Binding Events						
		Total	Species A binding events				Species B specific	Binding divergence
			All	Species A specific	Shared with Species B	Species A centric binding divergence		
D.mel	*D.sim*	543	499	93	406	18.64%	44	25.23%
D.mel	*D.yak*	558	499	141	358	28.26%	59	35.84%
D.mel	*D.pse*	539	499	385	114	77.15%	40	78.85%

Note: the pairwise binding divergence reported in the maintext are calculated as the percentage of *D. melanogaster* binding events (in the table, Species A) that is diverged from each of the non-*D. melanogaster* species (in the table, Species B) using parameter 0.4. 0.4 is a parameter derived empirically from a permutation-simulation procedure. 0.35 is a less stringent parameter while 0.5 , 1.0 are more strigent parameters. All binding events refer to all possible binding events identified in our pipeline; Two Way orthologous binding events are those binding events in regions where the pair wise DNA sequence identity >50%; Four way orthologous binding events are those binding events in regions where the DNA sequence identity between each pair >50%.
