# Supplementary material for: Adaptive Evolution and the Birth of CTCF Binding Sites in the Drosophila Genome
Source: PLoS Biol. 2012 Nov 6;10(11):e1001420. doi: 10.1371/journal.pbio.1001420 (PMC3491045; doi:10.1371/journal.pbio.1001420)
Supplement: Table S6 — Diverged and conserved CTCF binding events at high-sequence coverage sites. (PDF) [file pbio.1001420.s026.pdf]

Table S6: Diverged and conserved CTCF binding events at high sequence coverage sites

Parameter : 0.4

Species A	Species B	All Binding Events						
		Total	Species A binding events				Species B specific	Binding divergence
			All	Species A specific	Shared with Species B	Species A centric binding divergence		
D.mel	*D.sim*	2261	2107	347	1760	16.47%	154	22.16%
D.mel	*D.yak*	2326	2094	525	1569	25.07%	232	32.55%
D.mel	*D.pse*	1593	1492	1019	473	68.30%	101	70.31%

Species A	Species B	Two Way Orthologous Binding Events						
		Total	Species A binding events				Species B specific	Binding divergence
			All	Species A specific	Shared with Species B	Species A centric binding divergence		
D.mel	*D.sim*	2233	2082	338	1744	16.23%	151	21.90%
D.mel	*D.yak*	2295	2067	509	1558	24.63%	228	32.11%
D.mel	*D.pse*	1058	979	653	326	66.70%	79	69.19%

Species A	Species B	Four Way Orthologous Binding Events						
		Total	Species A binding events				Species B specific	Binding divergence
			All	Species A specific	Shared with Species B	Species A centric binding divergence		
D.mel	*D.sim*	1106	1010	146	864	14.46%	96	21.88%
D.mel	*D.yak*	1115	988	237	751	23.99%	127	32.65%
D.mel	*D.pse*	1014	938	622	316	66.31%	76	68.84%

Parameter : 0.35

Species A	Species B	All Binding Events						
		Total	Species A binding events				Species B specific	Binding divergence
			All	Species A specific	Shared with Species B	Species A centric binding divergence		
D.mel	*D.sim*	2542	2381	394	1987	16.55%	161	21.83%
D.mel	*D.yak*	2610	2362	600	1762	25.40%	248	32.49%
D.mel	*D.pse*	1805	1681	1097	584	65.26%	124	67.65%

Species A	Species B	Two Way Orthologous Binding Events						
		Total	Species A binding events				Species B specific	Binding divergence
			All	Species A specific	Shared with Species B	Species A centric binding divergence		
D.mel	*D.sim*	2506	2348	379	1969	16.14%	158	21.43%
D.mel	*D.yak*	2574	2332	581	1751	24.91%	242	31.97%
D.mel	*D.pse*	1201	1101	697	404	63.31%	100	66.36%

Species A	Species B	Four Way Orthologous Binding Events						
		Total	Species A binding events				Species B specific	Binding divergence
			All	Species A specific	Shared with Species B	Species A centric binding divergence		
D.mel	*D.sim*	1255	1154	169	985	14.64%	101	21.51%
D.mel	*D.yak*	1260	1125	275	850	24.44%	135	32.54%
D.mel	*D.pse*	1156	1059	666	393	62.89%	97	66.00%

Parameter : 0.5

Species A	Species B	All Binding Events						
		Total	Species A binding events				Species B specific	Binding divergence
			All	Species A specific	Shared with Species B	Species A centric binding divergence		
D.mel	*D.sim*	1890	1764	296	1468	16.78%	126	22.33%
D.mel	*D.yak*	1979	1765	434	1331	24.59%	214	32.74%
D.mel	*D.pse*	1327	1246	889	357	71.35%	81	73.10%

Species A	Species B	Two Way Orthologous Binding Events						
		Total	Species A binding events				Species B specific	Binding divergence
			All	Species A specific	Shared with Species B	Species A centric binding divergence		
D.mel	*D.sim*	1865	1742	286	1456	16.42%	123	21.93%
D.mel	*D.yak*	1950	1741	419	1322	24.07%	209	32.21%
D.mel	*D.pse*	869	806	574	232	71.22%	63	73.30%

Species A	Species B	Four Way Orthologous Binding Events						
		Total	Species A binding events				Species B specific	Binding divergence
			All	Species A specific	Shared with Species B	Species A centric binding divergence		
D.mel	*D.sim*	907	832	125	707	15.02%	75	22.05%
D.mel	*D.yak*	936	817	183	634	22.40%	119	32.26%
D.mel	*D.pse*	830	769	545	224	70.87%	61	73.01%

Parameter : 1.0

Species A	Species B	All Binding Events						
		Total	Species A binding events				Species B specific	Binding divergence
			All	Species A specific	Shared with Species B	Species A centric binding divergence		
D.mel	*D.sim*	1158	1158	203	879	17.53%	76	24.09%
D.mel	*D.yak*	1192	1192	281	791	23.57%	120	33.64%
D.mel	*D.pse*	796	796	572	172	71.86%	52	78.39%

Species A	Species B	Two Way Orthologous Binding Events						
		Total	Species A binding events				Species B specific	Binding divergence
			All	Species A specific	Shared with Species B	Species A centric binding divergence		
D.mel	*D.sim*	1140	1069	193	876	18.05%	71	23.16%
D.mel	*D.yak*	1175	1061	274	787	25.82%	114	33.02%
D.mel	*D.pse*	516	481	368	113	76.51%	35	78.10%

Species A	Species B	Four Way Orthologous Binding Events						
		Total	Species A binding events				Species B specific	Binding divergence
			All	Species A specific	Shared with Species B	Species A centric binding divergence		
D.mel	*D.sim*	534	491	89	402	18.13%	43	24.72%
D.mel	*D.yak*	539	481	125	356	25.99%	58	33.95%
D.mel	*D.pse*	491	457	347	110	75.93%	34	77.60%

Note: All items and sub-tables are all in the same formats as in Table S4. The number of conserved and diverged binding events reported here at different parameters are obtained from our analysis pipeline with an additional filtering of input sample sequence coverage at the identified peak sites >0.5. The final results of percentage of binding divergence remain the same as in Table S4
