# Supplementary material for: Adaptive Evolution and the Birth of CTCF Binding Sites in the Drosophila Genome
Source: PLoS Biol. 2012 Nov 6;10(11):e1001420. doi: 10.1371/journal.pbio.1001420 (PMC3491045; doi:10.1371/journal.pbio.1001420)
Supplement: Table S7 — CTCF binding divergence estimated by direct comparison. (PDF) [file pbio.1001420.s027.pdf]

Table S7: CTCF Binding divergence estimated by direct comparison

D. melanogaster centric

Species A	Species B	Total	Conserved with Species B	Diverged with Species B	binding divergence
D.mel	*D.sim*	2182	1424	758	34.74%
D.mel	*D.yak*	2182	1483	699	32.03%
D.mel	*D.pse*	2182	353	1829	83.82%

non-D.melanogaster centric

Species A	Species B	Total	Conserved with Species B	Diverged with Species B	binding divergence
D.sim	*D.mel*	2197	1381	816	37.14%
D.yak	*D.mel*	2993	1497	1496	49.98%
D.pse	*D.mel*	2332	388	1944	83.36%

Note: for the "*D. melanogaster* centric" subtable, we mapped each non-*D. melanogaster* species binding sites back to *D. melanogaster* genome using LiftOver, and calculated the percentage of *D .melagnogaster* binding sites that are not overlapping with any non-*D. melanogaster* LiftOver binding sites for each species pair as estimate of binding divergence. For the " non- *D. melanogaster* centric" subtable, we mapped the *D. melanogaster* binding sites to each non-*D. melanogaster* species genome using LiftOver, and calcualted the percentage of non-*D. melanogaste*r binding sites that are not overlapping with any *D. melanogaster* LiftOver binidng sites for each pair as estimate of binding divergence.
