# Supplementary material for: Adaptive Evolution and the Birth of CTCF Binding Sites in the Drosophila Genome
Source: PLoS Biol. 2012 Nov 6;10(11):e1001420. doi: 10.1371/journal.pbio.1001420 (PMC3491045; doi:10.1371/journal.pbio.1001420)
Supplement: Table S8 — CTCF binding divergence estimated using He et al. method. (PDF) [file pbio.1001420.s028.pdf]

Table S8: CTCF Binding divergence estimated using He *et al.* method

	CTCF		Twist	
	conservation	divergence	conservation	divergence
D.mel rep1	100.00%	0.00%	100.00%	0.00%
D.mel rep2	96.58%	3.42%	98.00%	2.00%
D.sim rep1	79.75%	20.25%	82.00%	18.00%
D.sim rep2	77.40%	22.60%	70.00%	30.00%
D.yak rep1	71.13%	28.87%	81.00%	19.00%
D.yak rep2	70.65%	29.35%	78.00%	22.00%
D.pse rep1	25.24%	74.76%	60.00%	40.00%
D.pse rep2	25.93%	74.07%	58.00%	42.00%
offset 20kb	10.42%	89.58%	13.00%	87.00%
D.mel pseudo1	1.76%	98.24%	NA	NA
*D.me*l pseudo2	18.49%	81.51%	NA	NA

Note: We followed the exact method as described in He *et al.* 2011 for estimating CTCF binding divergence/conservation. In order to match the experimental design of He *et al.*, We have randomly picked two replicates out of our three replicates data to perform the analyses. The *D. mel* pseudo samples are generated by randomly sampling exactly same number of reads as the ChIP samples from the corresponding input samples. The Twist conservation/divergence estimates are obtained from the He *et al.* 2011 paper. From our analyses, it is obvious that the He *et al.* method has a wide range of False Negative Rates (1.76% in one *D. mel* pseudo sample while 18.49% in the other) when applying to different data. Overall speaking, from this comparison, we observe higher CTCF binding divergence than Twist. Consistently, we observe the same pattern in the comparison when applying our methods to the Twist comparative data.
