# Supplementary material for: Adaptive Evolution and the Birth of CTCF Binding Sites in the Drosophila Genome
Source: PLoS Biol. 2012 Nov 6;10(11):e1001420. doi: 10.1371/journal.pbio.1001420 (PMC3491045; doi:10.1371/journal.pbio.1001420)
Supplement: Table S9 — Twist binding divergence estimated using our pipeline. (PDF) [file pbio.1001420.s029.pdf]

Table S9: Twist Binding divergence estimated using our pipeline

All Binding Events						
	Species A binding events					
Total	All	Species A specific	Shared with Species B	Species A centric binding divergence	Species B specific	Binding divergence
5320	5196	2061	3135	39.67%	124	41.07%
5850	5196	1931	3265	37.16%	654	44.19%
6825	5196	3074	2122	59.16%	1629	68.91%

Two Way Orthologous Binding Events						
	Species A binding events					
Total	All	Species A specific	Shared with Species B	Species A centric binding divergence	Species B specific	Binding divergence
5133	5009	1891	3118	37.75%	124	39.26%
5812	5158	1900	3258	36.84%	654	43.94%
5512	4072	2172	1900	53.34%	1440	65.53%

Four Way Orthologous Binding Events						
	Species A binding events					
Total	All	Species A specific	Shared with Species B	Species A centric binding divergence	Species B specific	Binding divergence
3932	3920	1446	2474	36.89%	12	37.08%
3995	3920	1427	2493	36.40%	75	37.60%
5303	3920	2086	1834	53.21%	1383	65.42%

Note: We applied our analysis pipeline to the Twist comparative data. Since there are only two ChIP replicates for each species, the data structure of the Twist comparative data is not ideal for applying our methods as the degree of freedom for ANOVA-like linear categorical model part is too small. We have even lower power in detecting non-*D. melanogaste*r species binding sites in pair wise comparisons.
