# Supplementary material for: Adaptive Evolution and the Birth of CTCF Binding Sites in the Drosophila Genome
Source: PLoS Biol. 2012 Nov 6;10(11):e1001420. doi: 10.1371/journal.pbio.1001420 (PMC3491045; doi:10.1371/journal.pbio.1001420)
Supplement: Table S10 — Summary of Twist binding divergence estimated using different methods. (PDF) [file pbio.1001420.s030.pdf]

Table S10: Summary of Twist Binding divergence estimated using different methods

	Pvalue 10-5	Pvalue 10-10	Pvalue 10-15	All	TWOB	FWOB
D.mel rep1	0	0	0	0	0	0
D.mel rep2	2%	6%	13%			
D.sim rep1	18%	29%	41%	39.7%	37.8%	36.9%
D.sim rep2	3%	52%	68%			
D.yak rep1	19%	28%	36%	37.2%	36.8%	36.4%
D.yak rep2	22%	34%	43%			
D.pse rep1	40%	48%	53%	59.2%	53.3%	53.2%
D.pse rep2	42%	53%	59%			
offset 20kb	87%	93%	95%	NA	NA	NA

Note: the divergence rate in column 2,3,4 are calculated from the conservation rate obtained from He *et al*. paper. These conservation rates were estimated by comparing the binding site sets identified using different P value cutoffs in other species to the high strigent binding site set identified in the *D. melanogaster* reference replicate (here *D. mel* rep1).Column 4,5,6 are the conservation rate estimated by applying our methods to the Twist data. We found that our estimates of conservation rate is generally higher than He *et al.* method, and consistent with the estimate variations as well as False Negative Rate (1- offset estimate) observed using their method between replicates for each species.
