# Supplementary material for: Adaptive Evolution and the Birth of CTCF Binding Sites in the Drosophila Genome
Source: PLoS Biol. 2012 Nov 6;10(11):e1001420. doi: 10.1371/journal.pbio.1001420 (PMC3491045; doi:10.1371/journal.pbio.1001420)
Supplement: Table S11 — Genomic distribution of different evolutionary groups of CTCF binding events. (PDF) [file pbio.1001420.s031.pdf]

Table S11: Genomic distribution of different evolutionary groups of CTCF binding events

Boolean conservation score	Genomic position			
	promoter	intergenic	intronic	exnoic
(1,1,1,1)	131	76	56	22
(1,1,1,0)	188	89	117	27
(1,1,0,1)	9	4	7	1
(1,0,1,1)	0	3	2	0
(1,1,0,0)	66	36	35	9
(1,0,1,0)	24	8	10	4
(1,0,0,1)	7	3	3	4
(1,0,0,0)	38	15	29	7
Total	463	234	259	74
