# Supplementary material for: Adaptive Evolution and the Birth of CTCF Binding Sites in the Drosophila Genome
Source: PLoS Biol. 2012 Nov 6;10(11):e1001420. doi: 10.1371/journal.pbio.1001420 (PMC3491045; doi:10.1371/journal.pbio.1001420)
Supplement: Table S12 — Parsimonious age dating of D. melanogaster CTCF binding events. (PDF) [file pbio.1001420.s032.pdf]

Table S12: Parsimonious age dating of *D. melanogaster* CTCF binding events

Boolean Conservation Score	Parsimony Reconstruction of Binding Events Evolutionary History	Age of D.mel binding
(1,1,1,1)	exist in the ancestor of the species	>25 Myr
(1,1,1,0)	exist in the ancestor , one loss event in the branch of *D.pseudoobscura*	>25 Myr
	one gain event after the split of *D.pse* and *melanogaster* group but before the split of *D.yak* and *melanogaste*r subgroup	>6Myr, <25Myr
(1,1,0,1)	exist in ancestor , one loss event in the branch of *D.yak*	>25Myr
(1,0,1,1)	exist in ancestor, one loss event in the branch of *D.sim*	>25Myr
(1,1,0,0)	one gain event after the split of *D.yak* and the *melanogaster* subgroup but before split of *D.sim* and *D.mel*	>2.5Myr , <6Myr
(1,0,1,0)	one gain in the branch of *D.me*l, one gain in the branch of *D.yak*	<2.5Myr
	exist in ancestor, one loss in *D.pse*, one loss in *D.sim*	>25Myr
(1,0,0,1)	one gain in the branch of *D.me*l, one gain in the branch of *D.pse*	<2.5Myr
	exist in ancestor, one loss in *D.yak*, one loss in *D.sim*	>25Myr
(1,0,0,0)	one gain in the branch of *D.mel*	<2.5Myr
