# Supplementary material for: Adaptive Evolution and the Birth of CTCF Binding Sites in the Drosophila Genome
Source: PLoS Biol. 2012 Nov 6;10(11):e1001420. doi: 10.1371/journal.pbio.1001420 (PMC3491045; doi:10.1371/journal.pbio.1001420)
Supplement: Table S13 — Evolutionary groups of CTCF binding events at high-sequence coverage sites. (PDF) [file pbio.1001420.s033.pdf]

Table S13:Evolutionary groups of CTCF binding events at high sequence coverage sites

With all four species

Boolean conservation score	All sites	FWOB sites
(1,1,1,1)	390	271
(1,1,1,0)	594	379
(1,1,0,1)	26	16
(1,1,0,0)	167	106
(1,0,1,1)	5	3
(1,0,0,1)	16	13
(1,0,1,0)	61	37
(1,0,0,0)	103	63
(0,1,1,1)	0	0
(0,1,1,0)	22	12
(0,1,0,1)	1	1
(0,1,0,0)	94	76
(0,0,1,1)	2	2
(0,0,0,1)	112	84
(0,0,1,0)	152	105

With three *D.melanogster* group species

Boolean conservation score	All sites	FWOB
(1,1,1)	1368	1346
(1,1,0)	288	278
(1,0,1)	107	103
(1,0,0)	201	195
(0,1,1)	23	15
(0,1,0)	117	74
(0,0,1)	194	105

Note: the number of binding events shown here are obtained after filtering out binding peaks with input sequence coverage <0.5. All notations for the tables are the same as for Figure 2 and Figure S7.
