# Supplementary material for: Adaptive Evolution and the Birth of CTCF Binding Sites in the Drosophila Genome
Source: PLoS Biol. 2012 Nov 6;10(11):e1001420. doi: 10.1371/journal.pbio.1001420 (PMC3491045; doi:10.1371/journal.pbio.1001420)
Supplement: Table S14 — Number of fixed and polymorphic mutations in CTCF-associated DNA sequences. (PDF) [file pbio.1001420.s034.pdf]

Table S14: Number of fixed and polymorphic mutations in CTCF associated DNA sequences

Using *D.simulans* as outgroup

CTCF associated DNA		Fixed	Polymorphism	F:P
	all binding	27465	11435	2.40
	TWOB	26538	11341	2.34
	Conserved TWOB	21110	9411	2.24
201bp region	Diverged TWOB	5428	1930	2.81
	FWOB	8610	4841	1.78
	Old FWOB	5926	3543	1.67
	Young FWOB	965	396	2.44
	all binding	4648	2221	2.09
	TWOB	4588	2214	2.07
	Conserved TWOB	3713	1865	1.99
motif region	Diverged TWOB	875	349	2.51
	FWOB	1619	1005	1.61
	Old FWOB	1132	743	1.52
	Young FWOB	170	57	2.98
Neutral control	Syn sites of genes	67149	36385	1.85

Using *D.yakuba* as outgroup

CTCF Associated DNA		Fixed	Polymorphism	F:P
	all binding	59994	11480	5.23
	TWOB	57187	11035	5.18
	Conserved TWOB	40576	8441	4.81
201bp region	Diverged TWOB	18090	2929	6.18
	FWOB	19106	4787	3.99
	Old FWOB	13361	3504	3.81
	Young FWOB	1976	388	5.09
	all binding	10219	2241	4.56
	TWOB	9792	2160	4.53
	Conserved TWOB	7024	1694	4.15
motif region	Diverged TWOB	3067	538	5.70
	FWOB	3638	966	3.77
	Old FWOB	2554	736	3.47
	Young FWOB	332	56	5.93
Neutral control	Syn sites of genes	182759	46702	3.91

Note: From the "F:P" values , we observe that the strength of positive selection in the CTCF-201bp sites is stronger than in CTCF-motif sites. There are two factors contributing to the increasing strength in the 201bp sites than the motif sites: 1) we used the position weight matrix of the 9bp core motif to find motif matches in the binding region. While the core motif contains most information in the 13~15bp enriched motif for *Drosophila* CTCF, the sequence itself is also more constrained; the greater number of fixed sites could come from the other less constrained motif sequences. 2) In *Drosophila*, different insulator proteins have a genome wide trend to

cluster in similar genomic regions, the higher positive selection strength in the binding region could result from selection on other insulator protein binding sites.
