# Supplementary material for: Adaptive Evolution and the Birth of CTCF Binding Sites in the Drosophila Genome
Source: PLoS Biol. 2012 Nov 6;10(11):e1001420. doi: 10.1371/journal.pbio.1001420 (PMC3491045; doi:10.1371/journal.pbio.1001420)
Supplement: Table S15 — Number of fixed and polymorphic mutations in CTCF-associated DNA sequences at high-sequence coverage sites. (PDF) [file pbio.1001420.s035.pdf]

Table S15: Number of fixed and polymorphic mutations in CTCF associated DNA sequences at high sequence coverage sites

Using *D.simulans* as outgroup

CTCF associated DNA		Fixed	Polymorphism	F:P
	all binding	27465	11435	2.40
	TWOB	24732	10831	2.28
	Conserved TWOB	20219	9072	2.23
201bp region	Diverged TWOB	4513	1759	2.57
	FWOB	7862	4560	1.72
	Old FWOB	5657	3424	1.65
	Young FWOB	823	356	2.31
	all binding	4648	2221	2.09
	TWOB	4347	2141	2.03
	Conserved TWOB	3608	1823	1.98
motif region	Diverged TWOB	875	349	2.51
	FWOB	1492	952	1.57
	Old FWOB	1088	721	1.51
	Young FWOB	140	51	2.75
Neutral control	Syn sites of genes	67149	36385	1.85

Using *D.yakuba* as outgroup

CTCF Associated DNA		Fixed	Polymorphism	F:P
	all binding	59994	11480	5.23
	TWOB	53512	10641	5.03
	Conserved TWOB	39417	8235	4.79
201bp region	Diverged TWOB	14095	2406	5.86
	FWOB	17765	4510	3.94
	Old FWOB	12832	3386	3.79
	Young FWOB	1678	352	4.77
	all binding	10219	2241	4.56
	TWOB	9261	2109	4.39
	Conserved TWOB	6833	1665	4.10
motif region	Diverged TWOB	2428	444	5.47
	FWOB	3414	948	3.60
	Old FWOB	2471	717	3.45
	Young FWOB	288	51	5.65
Neutral control	Syn sites of genes	182759	46702	3.91
