# Supplementary material for: Adaptive Evolution and the Birth of CTCF Binding Sites in the Drosophila Genome
Source: PLoS Biol. 2012 Nov 6;10(11):e1001420. doi: 10.1371/journal.pbio.1001420 (PMC3491045; doi:10.1371/journal.pbio.1001420)
Supplement: Table S16 — HKA test for old and young sites. (PDF) [file pbio.1001420.s036.pdf]

Table S16:HKA test for Old and Young sites

Species	Groups compared	Polymorphism		Divergence		Sum of Deviations (X^2)	P value
		Obseved	Expected	Observed	Expected		
D.mel/Dsim	Old	3496	3395.9	5684	5784.1		
	Young	391	491.0	925	825.0	1036.4	P<0.001
	Old	3496	3491.4	5684	5688.6		
	Small intron	1145	1148.1	1973	1969.9	1156.9	0.15< P<0.16
	Young	391	405.8	925	910.2		
	Small intron	135	120.3	273	287.7	172.4	P<0.001
D.mel/D.yak	Old	3504	3413.3	12784	12874.7		
	Young	388	478.7	1872	1781.3	1114.2	P<0.001
	Old	3504	3510.6	12784	12777.4		
	Small intron	1108	1101.8	4283	4289.2	1324.7	0.04<P<0.05
	Young	388	417.5	1872	1842.5		
	Small intron	145	115.6	510	539.4	202.5	P<0.01

Note: the observed and expected number of polymorphic and divergence sites are summarized over all sites withing each group, while the value of sum of deviation (X^2) is calculated by adding values of deviation for each loci together. The P value is calculated based on 1000 iterations of colacence simulations. When compared to Old sites and neutral small introns, the group of Young sites show significant reduced polymorphism, suggesting directional positive selection on them.
