# Supplementary material for: Adaptive Evolution and the Birth of CTCF Binding Sites in the Drosophila Genome
Source: PLoS Biol. 2012 Nov 6;10(11):e1001420. doi: 10.1371/journal.pbio.1001420 (PMC3491045; doi:10.1371/journal.pbio.1001420)
Supplement: Table S17 — HKA test for old and young high-sequence coverage sites. (PDF) [file pbio.1001420.s037.pdf]

Table S17:HKA test for Old and Young high sequence coverage sites

Species	Groups compared	Polymorphism		Divergence		Sum of Deviations (X^2)	P value
		Obseved	Expected	Observed	Expected		
D.mel/Dsim	Old	3377	3289.8	5416	5503.2		
	Young	351	438.6	810	722.4	996.5	P<0.001
	Old	3377	3395.1	5416	5397.9		
	Small intron	1147	1128.8	1886	1904.2	1124.6	0.15<P<0.19
	Young	351	370.9	810	790.1		
	Small intron	124	104.1	215	234.9	147.3	P<0.02
D.mel/D.yak	Old	3386	3310.1	12256	12332.0		
	Young	352	428.2	1647	1570.8	1071.0	P<0.001
	Old	3386	3407.8	12256	12234.2		
	Small intron	1099	1076.7	4115	4137.3	1334.2	0.03<P<0.04
	Young	352	404.1	1647	1594.9		
	Small intron	116	110.9	469	474.2	204.1	P<0.01

Note: the observed and expected number of polymorphic and divergence sites are summarized over all sites withing each group, while the value of sum of deviation (X^2) is calculated by adding values of deviation for each loci together. The P value is calculated based on 1000 interations of colacence simulation.All results shown here are obtained after filtering sites with input sequence coverage <0.5.When compared to Old sites and neutral small introns, the group of Young sites show significant reduced polymorphism, suggesting directional positive selection on them.
