# Supplementary material for: Adaptive Evolution and the Birth of CTCF Binding Sites in the Drosophila Genome
Source: PLoS Biol. 2012 Nov 6;10(11):e1001420. doi: 10.1371/journal.pbio.1001420 (PMC3491045; doi:10.1371/journal.pbio.1001420)
Supplement: Table S18 — Overlapping of CTCF binding sites with TE. (PDF) [file pbio.1001420.s038.pdf]

Table S18: Overlapping of CTCF binding sites with Transposable Elements

	All	*D. mel* lineage specific sites	other sites
Overlapping with TE	27	15	12
Not overlapping with TE	2240	246	1994
Total	2267	261	2006
Percentage of overlapping	1.19%	5.75%	0.60%

Note: We used the CTCF-201bp sites to identify the number of sites overlapping with annotated transposable elements in *Drosophila melanogaste*r genome.
